# The interplay between herbal medicines and gut microbiota in metabolic diseases

**DOI:** 10.3389/fphar.2023.1105405

**Published:** 2023-03-24

**Authors:** Lijie Wang, Xiaoling Gou, Yin Ding, Jingye Liu, Yue Wang, Yaqian Wang, Jing Zhang, Leilei Du, Wei Peng, Gang Fan

**Affiliations:** ^1^ State Key Laboratory of Southwestern Chinese Medicine Resources, School of Pharmacy, Chengdu University of Traditional Chinese Medicine, Chengdu, China; ^2^ School of Ethnic Medicine, Chengdu University of Traditional Chinese Medicine, Chengdu, China

**Keywords:** gut microbiota, metabolic disease, diabetes, obesity, non-alcoholic fatty liver disease, gout, hyperlipidemia, herbal medicine

## Abstract

Globally, metabolic diseases are becoming a major public health problem. Herbal medicines are medicinal materials or preparations derived from plants and are widely used in the treatment of metabolic diseases due to their good curative effects and minimal side effects. Recent studies have shown that gut microbiota plays an important role in the herbal treatment of metabolic diseases. However, the mechanisms involved are still not fully understood. This review provides a timely and comprehensive summary of the interactions between herbal medicines and gut microbiota in metabolic diseases. Mechanisms by which herbal medicines treat metabolic diseases include their effects on the gut microbial composition, the intestinal barrier, inflammation, and microbial metabolites (e.g., short-chain fatty acids and bile acids). Herbal medicines can increase the abundance of beneficial bacteria (e.g., *Akkermansia* and *Blautia*), reduce the abundance of harmful bacteria (e.g., *Escherichia*–*Shigella*), protect the intestinal barrier, and alleviate inflammation. In turn, gut microbes can metabolize herbal compounds and thereby increase their bioavailability and bioactivity, in addition to reducing their toxicity. These findings suggest that the therapeutic effects of herbal medicines on metabolic diseases are closely related to their interactions with the gut microbiota. In addition, some methods, and techniques for studying the bidirectional interaction between herbal medicines and gut microbiota are proposed and discussed. The information presented in this review will help with a better understanding of the therapeutic mechanisms of herbal medicines and the key role of gut microbiota.

## 1 Introduction

Metabolic diseases refer to disorders in the metabolic process of certain substances that are caused by overnutrition, sedentary lifestyles, and the resulting excess adiposity inside the human body (Eckel et al., 2005). Common metabolic diseases include type 2 diabetes mellitus (T2DM), obesity, non-alcoholic fatty liver disease (NAFLD), gout, and hyperlipidemia. T2DM accounts for approximately 90% of all diabetes cases, and its prevalence has dramatically increased worldwide. Obesity is an abnormal accumulation of fat that poses a health risk. It has sharply increased, especially among high-income individuals. NAFLD is one of the most important causes of chronic liver disease. It plays an important role in the development of end-stage liver disease (Younossi et al., 2018). Gout is an independent risk factor for heart failure. With economic development and changes in people’s diets, the prevalence of gout has been on the rise, and it has become a worrying public health problem (Chen et al., 2017). Hyperlipidemia is a systemic metabolic disease that is involved in the occurrence and development of cardiovascular and cerebrovascular diseases. The risk factors for metabolic diseases are traditionally thought to be genetics, unhealthy diets, and sedentary lifestyles. However, recent research reveals that gut microbes and their metabolites are involved in the pathogenesis of metabolic diseases (Wu et al., 2021; Du et al., 2022). Therefore, targeting the gut microbiota provides a new strategy for the treatment of metabolic diseases.

Herbal medicines are plant-derived materials or preparations that play an important role in the treatment of chronic diseases. Recent studies demonstrate that the therapeutic effects of herbal medicines on metabolic diseases are closely related to their interaction with the gut microbiota (Xu et al., 2015; Du et al., 2021; Xu et al., 2021). On the one hand, herbal medicines are able to regulate the composition of the gut microbiota and the levels of gut microbial metabolites to improve diseases (Wei et al., 2018; Xu et al., 2021). On the other hand, gut microbes can transform phytochemicals in herbal medicines to produce specific metabolites with different bioavailability, bioactivity, and toxicity (Chen et al., 2015; Feng et al., 2015). These interactions suggest that the gut microbiota may play a pivotal role in the herbal treatment of metabolic diseases. In this review, we provide a comprehensive overview of the mechanisms by which herbal medicines, including plant materials and herbal preparations, interact with the gut microbiota in the treatment of metabolic diseases. Furthermore, certain methodologies for studying the bidirectional interaction between herbal medicines and gut microbiota are proposed and discussed. This information will contribute to a better understanding of the therapeutic mechanisms of herbal medicines and provide novel insights into the development of targeted therapies for metabolic diseases based on gut microbiota.

## 2 Methods

A literature search was conducted using a number of electronic databases, including the Web of Science, PubMed, ScienceDirect, Google Scholar, and the China National Knowledge Infrastructure. When searching the literature, the following terms were used as keywords: “herbal medicine,” “botanical medicine,” “traditional medicine,” “metabolic disease,” “metabolic syndrome,” “diabetes,” “obesity,” “non-alcoholic fatty liver disease,” “gout,” “hyperlipidemia,” and “gut microbiota.” Following the literature search, the full texts were carefully examined to determine the eligibility for inclusion in this review. Conference abstracts, editorials, and studies with unavailable or incomplete data were excluded. Several online databases (http://mpns.kew.org/mpns-portal/, http://www.theplantlist.org/, or http://www.plantsoftheworldonline.org) were used to validate and standardize the Latin names of the plant species. In addition, the composition of each herbal preparation is presented in detail in Supplementary Table S1 (see Supplementary Tables).

## 3 Results and discussion

### 3.1 The mechanisms by which herbal medicines treat metabolic diseases by regulating the gut microbiota

In the following sections, we categorically describe the mechanisms by which herbal medicines target the gut microbiota to treat five metabolic diseases, including their effects on microbial composition, the intestinal barrier, inflammation, and gut microbiota-derived metabolites. Detailed results are shown in Supplementary Table S2 for T2DM, Supplementary Table S3 for obesity, Supplementary Table S4 for NAFLD, Supplementary Table S5 for gout, and Supplementary Table S6 for hyperlipidemia (see Supplementary Tables).

#### 3.1.1 Regulation of the gut microbiota composition

**FIGURE 1:**
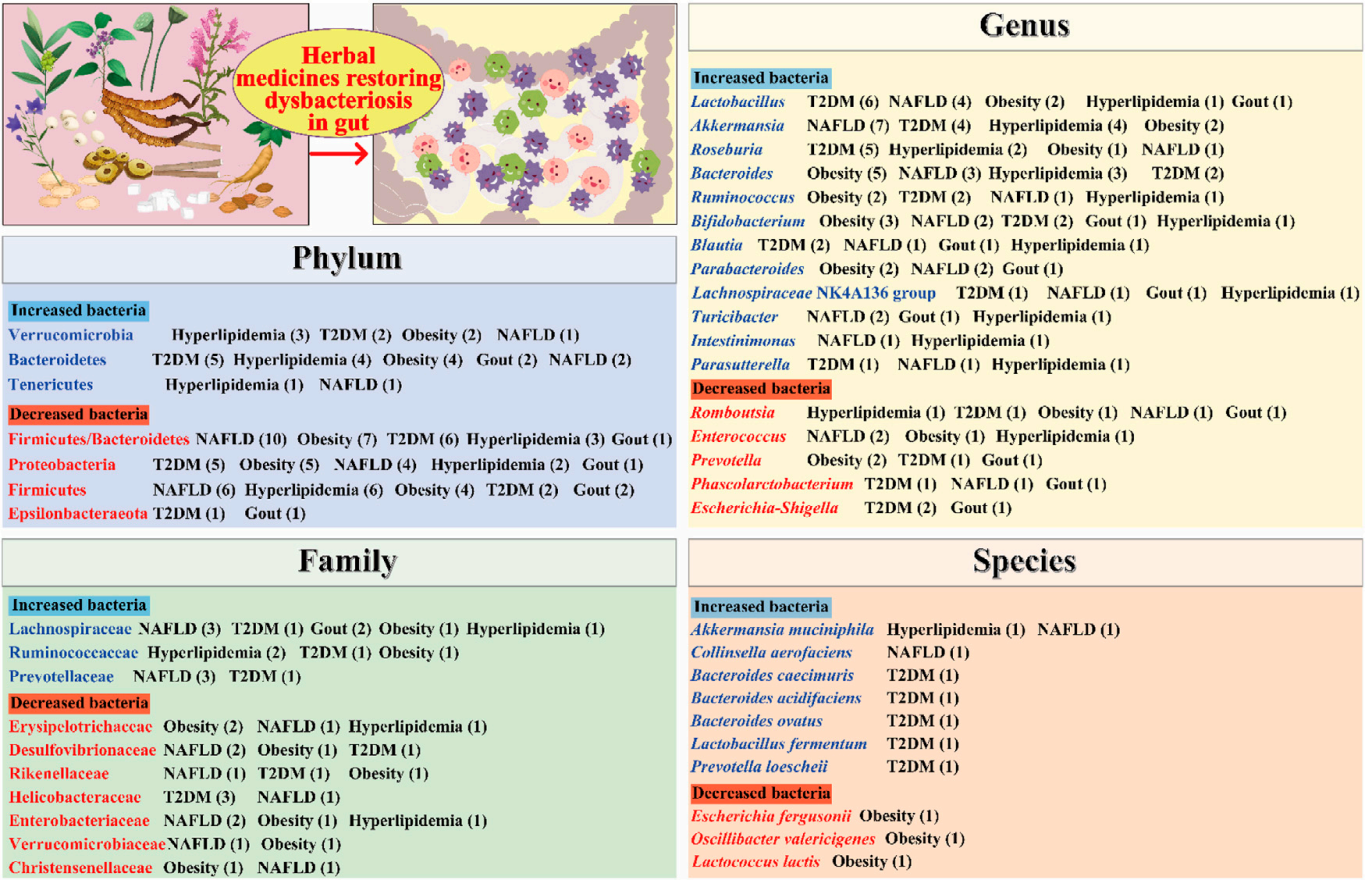
Herbal medicines regulate the composition of the gut microbiota. The numbers in parentheses represent the number of current studies on herbal remedies for the corresponding metabolic diseases. T2DM, type 2 diabetes mellitus; NAFLD, non-alcoholic fatty liver disease.

Homeostasis of the gut microbiota is often disrupted in patients with metabolic diseases. Numerous studies demonstrate that herbal medicines can modulate the composition of the gut microbiota and restore gut homeostasis in rodents with metabolic diseases (Figure 1). Subsequently, we summarized the major intestinal bacteria regulated by herbal medicines at different classification levels and discussed their association with metabolic diseases.

At the phylum level, Firmicutes and Bacteroidetes are the most abundant bacteria. An elevated ratio of Firmicutes to Bacteroidetes (F/B) in the gut microbiota has been linked to metabolic diseases such as T2DM, hyperlipidemia, obesity, and NAFLD (Ansari et al., 2016; Chen et al., 2018; Li et al., 2019a; Zhang et al., 2022a). Some herbal medicines have been shown to reduce the F/B ratio. For example, *Alisma orientale* (Sam.) Juz. (Alismataceae; Alismatis Rhizoma) decreased the F/B ratio and improved gut microbiota dysbiosis in hyperlipidemic rats induced through a high-fat and high-sucrose diet (Li et al., 2019a). Similarly, taking the Linggui Zhugan formula reduced the F/B ratio in diabetic mice (Liu et al., 2019). Moreover, Proteobacteria can cause inflammatory responses and lead to metabolic disorders (Shin et al., 2015). Studies showed that *Gynostemma pentaphyllum* (Thunb.) Makino (Cucurbitaceae; Gynostemmae Pentaphylli Herba) and Jiangzhi granules could reduce the abundance of Proteobacteria in NAFLD mice (Jia et al., 2018a; Wang et al., 2021).

At the family level, Lachnospiraceae bacteria are major producers of short-chain fatty acids (SCFAs), which are beneficial in improving metabolic diseases (Du et al., 2022). A study demonstrated that Jiangzhi granules could regulate the gut microbiota disturbance in NAFLD mice and resulted in a significant increase in the abundance of Lachnospiraceae bacteria (Wang et al., 2021). Ruminococcaceae bacteria are known to degrade mucin and ferment carbohydrates, which results in the production of beneficial acetate and propionate (Paone and Cani, 2020). Li et al. (2021b) reported that the Jieyu Qutan Huazhuo formula significantly increased the abundance of Ruminococcaceae bacteria in hyperlipidemia mice. Similarly, Zhang et al. (2019) demonstrated that *Spatholobus suberectus* Dunn (Leguminosae; Spatholobi Caulis) could increase the abundance of Ruminococcaceae bacteria in diet-induced obese mice. In addition, Desulfovibrionaceae bacteria are capable of producing harmful H_2_S and lipopolysaccharide (LPS), which can damage intestinal epithelial cells (Christophersen et al., 2011; Liu et al., 2014). One study revealed that *Momordica charantia* L. (Cucurbitaceae; Momordicae charantiae fructus) significantly reduced the number of Desulfovibrionaceae bacteria in obese rats (Bai et al., 2016).

At the genus level, *Akkermansia* bacteria are beneficial microorganisms. They can degrade mucin and produce propionic acid, thus protecting the intestinal mucosal barrier (Cui et al., 2019). Moreover, *Akkermansia* bacteria are associated with a reduced risk of obesity (Zhou et al., 2020). Notably, Xu et al. (2021) demonstrated that herbal medicine, *Berberis kansuensis* C.K. Schneid. (Berberidaceae; Berberis Cortex), significantly increased the abundance of *Akkermansia* bacteria in T2D rats. Similarly, Li et al. (2022) reported that *Penthorum chinense* Pursh (Saxifragaceae; Penthori Chinensis Herba) could increase the abundance of *Akkermansia* bacteria in the feces of NAFLD mice. *Blautia* bacteria possess potential probiotic properties that help regulate host health and alleviate metabolic syndrome (Liu et al., 2021). It was reported that the Shenqi compound could increase the abundance of *Blautia* bacteria and its metabolites in model rats and thereby improve T2DM (Zhang et al., 2022b). Furthermore, *Escherichia*–*Shigella* bacteria are a class of opportunistic pathogens associated with inflammation. Xiao et al. (2020) found that *Scutellaria baicalensis* Georgi (Labiatae; Scutellariae Radix) and *Coptis chinensis* Franch. (Ranunculaceae; Coptidis Rhizoma) could significantly reduce the number of *Escherichia*–*Shigella* in the feces of diabetic rats.

At the species level, *Akkermansia muciniphila*, which is a well-known beneficial microbe, has been shown to be effective in improving metabolic diseases such as T2DM and NAFLD (Yan et al., 2021). One study demonstrated that *A. muciniphila* reversed high-fat diet (HFD)-induced metabolic endotoxemia, adipose tissue inflammation, and insulin resistance (Everard et al., 2013). Notably, some herbal medicines have been found to increase the abundance of *A. muciniphila* and improve metabolic diseases. For example, Yang et al. (2022a) reported that taking *C. chinensis* Franch. (Ranunculaceae; Coptidis Rhizoma) for 4 weeks could increase the number of *A. muciniphila* in the feces of hyperlipidemic mice. Moreover, *Parabacteroides goldsteinii* is considered a probiotic that can ameliorate obesity and its associated metabolic disorders (Chang et al., 2019). One study found that taking the mycelium of *Ganoderma lucidum* (Leyss. ex Fr.) Karst (Polyporaceae; *Ganoderma*) for 12 weeks could increase the number of *P. goldsteinii* and reduce body weight, inflammation, and insulin resistance in obese mice (Chang et al., 2015).

#### 3.1.2 Regulation of the intestinal barrier and inflammation

Homeostasis of the intestinal barrier is vital for human health. When the integrity of the intestinal barrier is compromised, toxins from gut microbes (e.g., LPS) can escape from the intestinal lumen into the bloodstream (Guerville et al., 2017). LPS is a glycolipid component of the outer membrane of gram-negative bacteria (Carnevale et al., 2019). LPS can activate the TLR4/MyD88/NF-κB signaling pathway to release pro-inflammatory indicators (e.g., IL-6, IL-1β, and TNF-α) and thus trigger a cascade of inflammatory processes that ultimately lead to chronic low-grade inflammation (Mehta et al., 2010; Mohammad and Thiemermann, 2021). It is well known that chronic inflammation is a pathogenic factor in metabolic diseases (Hotamisligil et al., 2006). Thus, strengthening the function of the intestinal barrier and reducing inflammation is beneficial for ameliorating metabolic diseases.

**FIGURE 2:**
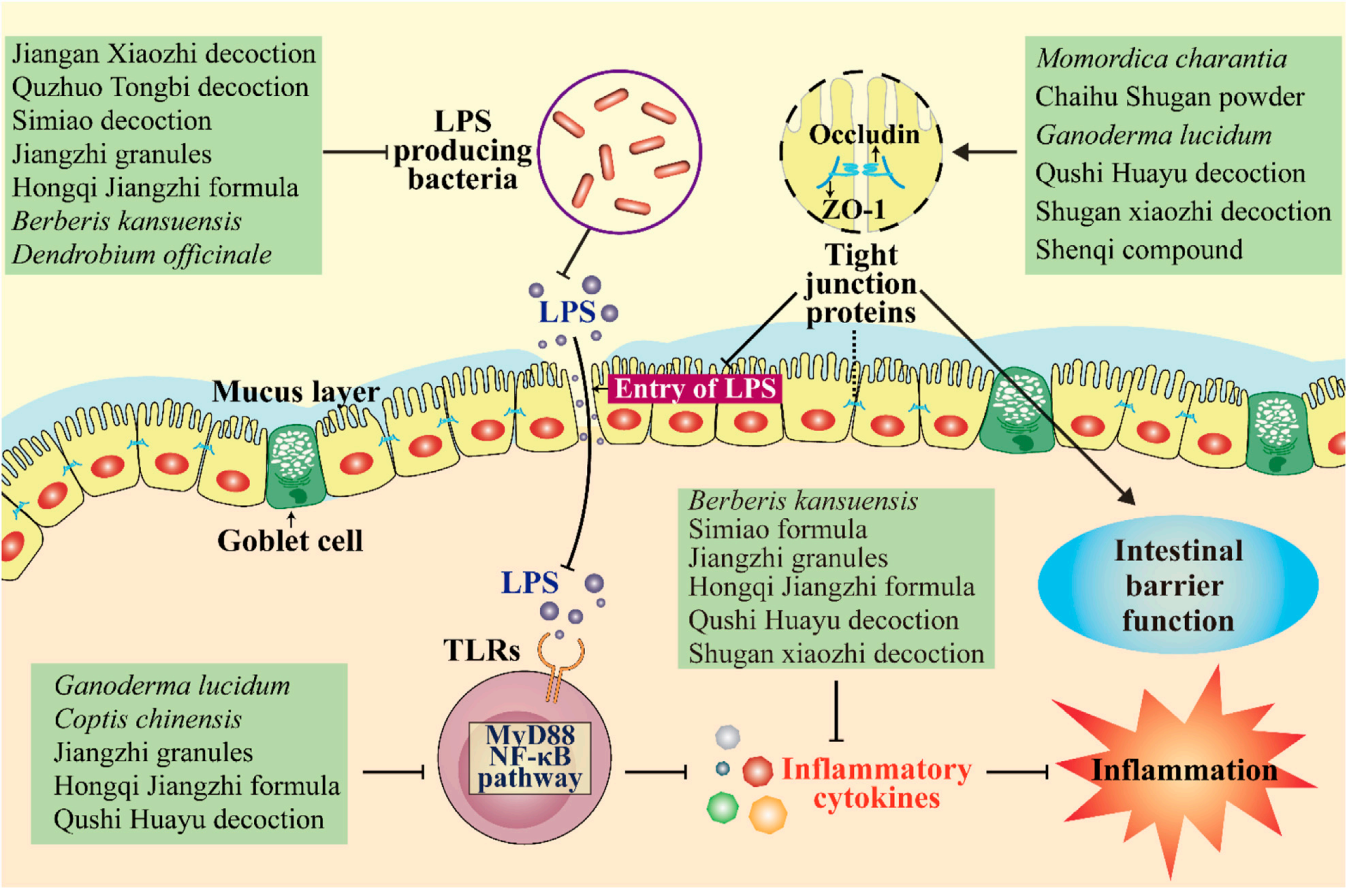
Herbal medicines regulate the intestinal barrier and inflammation. They protect the intestinal mucosal barrier by upregulating the expression of several tight junction proteins and thereby inhibiting LPS gut leakage. They can also inhibit LPS production by reducing the number of LPS-producing bacteria. Moreover, some herbal medicines improve inflammation by inhibiting the MyD88/NF-κB pathway and reducing levels of inflammatory cytokines. ↓, increase or activate; ⊥, decrease or suppress.

Tight junctions, a key component of the intestinal barrier, are essential for preventing the transmission of harmful molecules (Meijers et al., 2018). Studies have shown that the decreased expression of tight junction proteins, such as occludin, zonula occludens-1 (ZO-1), and claudin-1, can lead to an impaired epithelial barrier function and increased intestinal permeability (Hossain and Hirata, 2008; McGuckin et al., 2009). Some herbal medicines have been shown to protect the intestinal mucosal barrier by upregulating the expression of several tight junction proteins (Figure 2). For example, Jiangan Xiaozhi decoction, Quzhuo Tongbi decoction, Simiao decoction, Jiangzhi granules, and Hongqi Jiangzhi formula can upregulate the expression of tight junction-associated proteins occludin and ZO-1 in intestinal tissues of rodents with metabolic diseases (Liang et al., 2018; Liao et al., 2020; Han et al., 2021; Wang et al., 2021; Wen et al., 2021). Similarly, Qushi Huayu decoction was found to protect the intestinal barrier function by upregulating the mRNA expression of ZO-1, occludin, and claudin-1 and thereby inhibiting LPS gut leakage and ultimately improving non-alcoholic steatohepatitis induced by a high-fat diet in mice (Leng et al., 2020). In addition to blocking LPS passage through the intestinal barrier, some herbal medicines can directly inhibit LPS production by reducing the number of LPS-producing bacteria such as *Escherichia–Shigella*, *Enterococcus*, *Desulfovibrio*, *Klebsiella*, and *Enterobacter*. *Berberis kansuensis* C.K. Schneid. (Berberidaceae; Berberis Cortex) significantly improved T2DM, which was associated with reduced inflammation, by lowering the abundance of *Escherichia–Shigella* and *Enterococcus* and levels of LPS, TNF-α, IL-1β, and IL-6 in rats (Xu et al., 2021). Similarly, *Dendrobium officinale* Kimura & Migo (Orchidaceae; Dendrobii Officinalis Caulis) accelerated liver recovery in NAFLD mice by regulating the gut microbiota and gut–liver axis signaling pathways, including reducing the relative abundance of *Desulfovibrio* by 76.1% and the level of microbial LPS (Lei et al., 2019).

#### 3.1.3 Regulation of gut microbiota-derived metabolites

**FIGURE 3:**
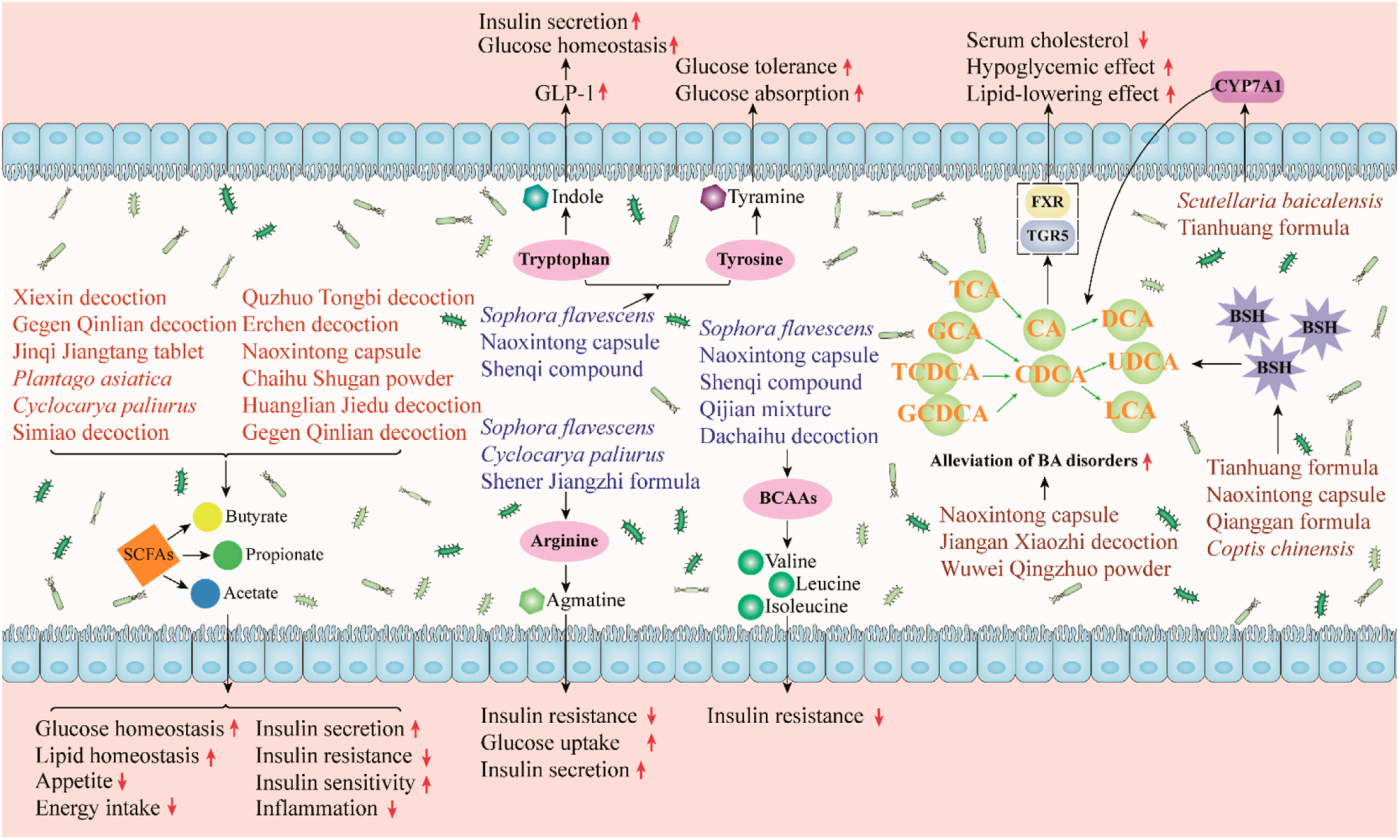
Herbal medicines regulate gut microbiota-derived metabolites, and they can regulate gut microbiota metabolites to alleviate metabolic diseases. SCFAs can improve glucose and lipid homeostasis, promote insulin secretion and sensitivity, and reduce appetite, energy intake, and insulin resistance. Indole can promote glucose homeostasis and insulin secretion. Tyramine, which is transformed from tyrosine, increases glucose tolerance and absorption. Agmatine can enhance glucose uptake and insulin secretion. Agmatine and BCAAs can reduce insulin resistance. Herbal medicines can relieve BA disorders by regulating BSH and CYP7A1 thereby improving the symptoms of metabolic diseases.

The gut microbiota can affect human health and disease by producing some bioactive metabolites such as SCFAs and bile acids (BAs). Next, we categorically summarized the major microbial metabolites regulated by herbal medicines and discussed their roles in metabolic diseases (Figure 3).

1) SCFAs

SCFAs, which are organic fatty acids composed of 1 to 6 carbon atoms, are a major category of microbial metabolites produced by the fermentation of bacteria in the cecum and colon (Koh et al., 2016). SCFAs, including acetate, butyrate, and propionate, have been shown to be beneficial in improving metabolic disorders. Butyrate and propionate can activate intestinal gluconeogenesis through the cAMP-dependent pathway, thereby reducing hepatic glucose production (De Vadder et al., 2014). Acetate inhibits liver lipid accumulation by upregulating several fatty acid oxidation-related proteins and the peroxisome proliferator-activated receptor α (PPARα) gene (Kondo et al., 2009). In addition, SCFAs can inhibit energy intake and appetite through the promotion of the production of satiety hormones. Acetate, butyrate, and propionate stimulate the release of peptide YY (PYY) and glucagon-like peptide-1 (GLP-1) from enteroendocrine L cells and promote the release of the satiety hormone leptin from adipose tissue (den Besten et al., 2015; Larraufie et al., 2018; Xiong et al., 2004). SCFAs can also ameliorate diet-induced insulin resistance, promote pancreatic insulin secretion, and improve insulin sensitivity (Canfora et al., 2015; Mandaliya and Seshadri, 2019). Furthermore, SCFAs can alleviate inflammation. For example, butyrate significantly reduced the production of TNF-α, monocyte chemoattractant protein 1 (MCP-1), and IL-6 and inhibited the activity of NF-κB (Ohira et al., 2013).

A number of herbal medicines have been shown to target the gut microbiome, increase SCFA levels, and thereby improve metabolic diseases. The administration of Xiexin decoction, Jinqi Jiangtang tablet, Gegen Qinlian decoction, *Plantago asiatica* L. (Plantaginaceae; Plantaginis Semen), and *Cyclocarya paliurus* (Batalin) Iljinsk. (Juglandaceae; Cylocaryae Paliuri Folium) can increase the abundance of several SCFA-producing bacteria and the level of SCFAs in T2DM rats or mice (Wei et al., 2018; Cao et al., 2019; Nie et al., 2019; Xu et al., 2020; Li et al., 2021c). Meanwhile, these herbal medicines reduce lipid levels, insulin resistance, and inflammation in the model animals. Similarly, Simiao decoction was reported to increase the abundance of SCFA-producing bacteria (*Bifidobacterium* and *Faecalibaculum*) and improve lipid metabolism and inflammation in NAFLD rodents (Han et al., 2021). Moreover, Quzhuo Tongbi decoction effectively alleviated gouty arthritis in mice by increasing the abundance of butyrate-producing bacteria and the levels of acetate, propionate, and butyrate (Wen et al., 2021).

2) Amino acids

The gut microbiota is also involved in the metabolism of amino acids *in vivo*. Studies have shown that the disturbance of microbial amino acid metabolism is related to the progression of metabolic diseases (Neis et al., 2015). Tryptophan can be metabolized into indole by some gut bacteria. Indole has a variety of beneficial effects on metabolic diseases; it can regulate the secretion of GLP-1 by intestinal endocrine L cells and thereby promote insulin secretion and improve glucose homeostasis (Pols et al., 2010; Du et al., 2022). Tyrosine can be converted into tyramine by the decarboxylase of the gut microbiota (Liu et al., 2020). Tyramine has been reported to improve glucose tolerance and glucose absorption (Morin et al., 2002; Visentin et al., 2003). In addition, arginine is converted into agmatine by the gut microbiota. Studies have confirmed the beneficial effects of arginine and its metabolite agmatine on metabolic diseases, including the promotion of insulin secretion, improving insulin resistance, and increasing cellular glucose uptake (Okazaki, et al., 2019; Du et al., 2022). Notably, some herbs have been found to regulate the metabolism of these amino acids in rodents with metabolic diseases. *Sophora flavescens* Ait. (Leguminosae; Sophorae Flavescentis Radix) significantly decreased the levels of fasted blood glucose, glycosylated serum protein, and glycosylated hemoglobin in T2DM rats by regulating gut bacteria and host-microbial metabolism, including increasing indole and tyramine levels (Shao et al., 2020). Similarly, the Naoxintong capsule improved hyperglycemia and hyperlipidemia in T2D rats, and its anti-diabetic mechanisms are related to the improvement of gut microbial disorders and regulation of tyrosine and tryptophan biosynthesis (Yan et al., 2020). Moreover, Shao et al. (2020) reported that *S. flavescens* Ait. (Leguminosae; Sophorae Flavescentis Radix) had good anti-diabetes effects on T2DM rats and might upregulate arginine and proline metabolism by reducing the abundance of *Prevotella*, *Roseburia*, and *Faecalibacterium*.

Branched-chain amino acids (BCAAs), such as leucine, isoleucine, and valine, have been shown to be closely associated with metabolic disease. Yu et al. (2022) found that a high intake of BCAAs was associated with a higher risk of T2DM. In addition, Zhou et al. (2019) demonstrated the pathogenic role of elevated BCAA levels in obesity-related insulin resistance. Some gut microbes, such as *Streptococcus* and *Prevotella*, are involved in BCAA biosynthesis and catabolism. One study found that berberine improved glycemic control and alleviated insulin resistance in HFD-fed mice, which was associated with altered gut microbiota in BCAA biosynthesis (Yue et al., 2019). Specifically, berberine treatment significantly reduced the relative abundance of BCAA-producing bacteria and serum BCAA levels. Meanwhile, berberine reduced the gut microbial genes involved in BCAA biosynthesis but enriched the genes involved in BCAA degradation and transport. In addition, Gao et al. (2018) evaluated the effects of the Qijian mixture on T2DM mice using metabonomics, gut microbiota, and network pharmacology. The results showed that the Qijian mixture significantly alleviated T2DM, and its anti-diabetic mechanisms were related to the regulation of gut microbiota and the reduction of several amino acids, including three BCAAs (leucine, isoleucine, and valine).

3) Bile acids

Cholesterol is converted to BAs in the liver under the action of cholesterol 7α-hydroxylase (CYP7A1) and CYP27A1. When BAs are secreted into the intestine, gut microbes can participate in their metabolism and maintain their homeostasis (Wahlstrom et al., 2016). For example, conjugated BAs, such as tauro-conjugated β-MCA (T-β-MCA) and glycoursodeoxycholic acid (GUDCA), can be converted into secondary BAs under the action of the bile salt hydrolase (BSH) of some gut bacteria, including *Clostridium*, *Bacteroides*, *Lactobacillus*, and *Bifidobacterium* (Jia et al., 2018b). BAs play an important role in glucose and lipid metabolism by acting on two receptors, namely, the farnesoid X receptor (FXR) and Takeda G protein-coupled receptor 5 (TGR5). Bile acid metabolism disorder has been shown to be closely related to the progression of metabolic diseases (Cai et al., 2022).

Several herbal medicines have been reported to regulate gut bacteria-related bile acid metabolism. The Tianhuang formula showed a lipid-lowering effect through the gut microbiota–T-β-MCA–FXR axis (Yang et al., 2022a). Specifically, it regulated gut microbes and inhibited their BSH activities, which thereby increased T-β-MCA levels and further inhibited intestinal FXR, which lead to increased bile acid synthesis and reduced lipid levels. Similarly, Lu et al. (2022) found that supplementation with Naoxintong capsule could effectively improve hyperlipidemia in HFD-fed rats, and its effect was related to the regulation of gut microbes and the decrease of BSH activity. In addition, *S. baicalensis* Georgi (Labiatae; Scutellariae Radix) improved hyperglycemia and hyperlipidemia in T2DM rats by regulating the interaction between gut microbiota and bile acid metabolism (Zhao et al., 2021). Specifically, the administration of *S. baicalensis* Georgi (Labiatae; Scutellariae Radix) significantly improved gut microbiota dysregulation (e.g., *Lactobacillus* and *Feacalibaculum*) and secondary BA metabolism disorders (e.g., GUDCA) by activating liver CYP7A1 and inhibiting FXR in the gut.

### 3.2 *In vivo* metabolism of herbal medicines by the gut microbiota

Similar to the liver, the gut plays an important role in the metabolism of oral drugs. After oral administration, herbal medicines contact and interact with gut microbes in the colon. The gut microbiota harbors many types of enzymes, such as glycoside hydrolase, oxidase, reductase, and esterase, which can metabolize and transform the chemical components of herbal medicines. These biotransformations may enable herbs to have better bioavailability and bioactivity or less toxicity (Feng et al., 2019; Whang et al., 2019; Javdan et al., 2020). Next, we categorically describe the different mechanisms by which gut microbes influence the metabolism and efficacy of some herbal medicines.

#### 3.2.1 The gut microbiota enhances the bioavailability and bioactivity of herbal medicines

Phytochemicals in herbal medicines are generally low in bioavailability, but some metabolites that are transformed by the gut microbiota may exhibit better bioavailability than their precursors. Ellagitannins, for example, are a group of polyphenols found in pomegranates and *Phyllanthus emblica* L. (Euphorbiaceae; Phyllanthi Fructus) that have low bioavailability. However, their gut microbial metabolites, urolithins (urolithin A, B, C, and D), are more readily absorbed and have better bioavailability than ellagitannins (Espin et al., 2007; Espin et al., 2013). Interestingly, urolithins (e.g., urolithin A and B) have been shown to have beneficial therapeutic effects on metabolic diseases, including dyslipidemia, obesity, and diabetes (Raimundo et al., 2021; Toney et al., 2021; Chen et al., 2022). Thus, the therapeutic effect of pomegranates and *P. emblica* L. (Euphorbiaceae; *P. fructus*) on metabolic diseases may be attributed to urolithins produced by the gut microbiota rather than the polyphenols they contain. In addition, some metabolites produced by gut microbiota may have better bioactivity than their precursors. For instance, protopanaxadiol-type ginsenosides, including ginsenoside Rb1 in *Panax ginseng*, can be metabolized by the gut microbiota into compound K. There is increasing evidence that compound K has a good anti-diabetic effect (Jiang et al., 2014; Shao et al., 2015). In particular, several studies have shown that compound K has better antidiabetic, anti-inflammatory, and hepatoprotective activities than protopanaxadiol-type ginsenosides or ginsenoside Rb1 (Lee et al., 2005; Joh et al., 2011; Li et al., 2012). These findings help elucidate the key role of gut microbes in herbal treatments for metabolic diseases.

#### 3.2.2 The gut microbiota reduces the toxicity of herbal medicines

The toxicity or side effects of herbal medicines have aroused wide concern. The gut microbiota can convert some herbal compounds into less toxic metabolites. Aconitine is a well-known toxic ingredient found in *Aconitum* medicinal plants. Aconitine can be metabolized to benzoylaconine and lipoaconitine by human gut bacteria through deacetylation, demethylation, and esterification reactions. and thus reduce its toxicity (Kawata et al., 1999; Zhao et al., 2008; Zhang et al., 2015). Baicalin is the main active ingredient of *S. baicalensis* Georgi (Labiatae; Scutellariae Radix). Studies have shown that baicalin can be converted into baicalein by the gut microbiota, and baicalein has less toxicity on HepG2 cells than baicalin (Khana et al., 2012). Notably, baicalein has hepatoprotective, anti-dyslipidemia, anti-obesity, anti-inflammatory, and anti-diabetic activities (Fang et al., 2020; Rahimi et al., 2021). In addition to these direct transforming effects, some metabolites derived from gut bacteria also help reduce the toxicity of herbal medicines. Triptolide, which is a natural compound isolated from *Tripterygium wilfordii* Hook F (Celastraceae; Triptergii Radix et Rhizoma), has good anti-inflammatory and neuroprotective activities (Li et al., 2014). It also ameliorates hepatic lipogenesis, inflammation, and fibrosis in NAFLD (Huang et al., 2021). However, its clinical application is limited due to its severe hepatotoxicity. Recently, a study found that gut microbiota-derived propionate could ameliorate triptolide-induced hepatotoxicity (Huang et al., 2020). Specifically, propionate supplementation significantly reduces plasma transaminase, improves liver histology, and decreases liver and plasma malondialdehyde (MDA) levels.

### 3.3 Methodology to study the bidirectional interaction between herbal medicines and the gut microbiota

**FIGURE 4:**
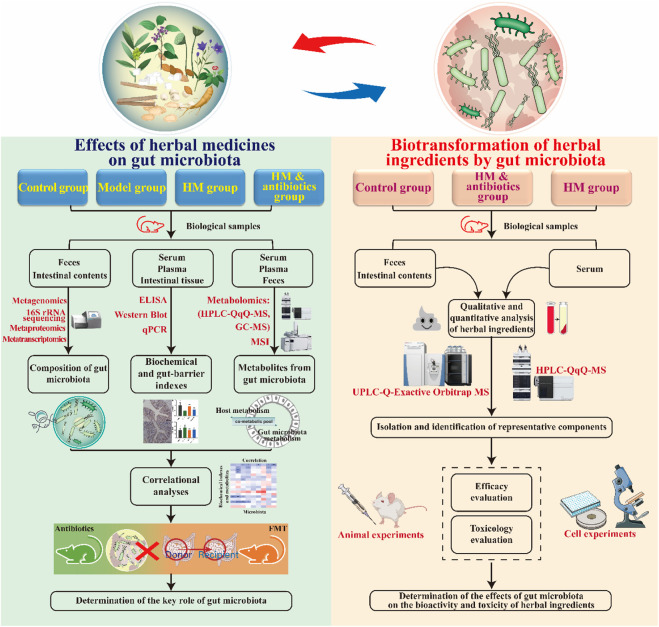
Methodology for studying the bidirectional interaction between herbal medicines and the gut microbiota. HM, herbal medicine.

As described above, the therapeutic effects of herbal medicines on metabolic diseases are closely related to their interaction with the gut microbiota. With the development of science and technology, multidisciplinary techniques and methods can be used to study the complex relationship between herbs, gut microbiota, and diseases. Next, we summarize and discuss some techniques and methods for studying the bidirectional interactions between herbal medicines and gut microbiota (Figure 4).

#### 3.3.1 Methodology to study the effects of herbal medicines on the gut microbiota

To determine the therapeutic effect of herbal medicines on metabolic diseases and the key role of the gut microbiota, four experimental groups were created: a control group, a model group, a herbal medicine group, and an herbal medicine plus antibiotic group. After the experiment, common biochemical indicators, gut microbiota, and microbial metabolites in each group were detected and analyzed. Changes in the composition and diversity of the gut microbiota in each group were studied using 16S ribosomal RNA (rRNA) sequencing and/or metagenomics techniques. The 16S rRNA technique can be used to detect bacteria in samples based on polymerase chain reaction (PCR) amplification. The main challenges in using this technique are the lack of a standardized workflow and the difficulty in identifying bacteria at the species level. Metagenomics is a widely used technique that can identify microorganisms at the species and even strain level (Jovel et al., 2016). In addition, it can also perform a functional analysis of microbial communities. However, methodological biases and inter-individual differences must be considered during data interpretation. Recently, advanced techniques have been developed to study the composition and function of gut microbes. For example, metatranscriptomics can provide knowledge of the transcriptional profiles of microbial populations, which is beneficial in revealing the molecular activities of gut microbes and their regulatory mechanisms (Zhang et al., 2021). Similarly, metaproteomics is a powerful tool that can be used to study the functional activity of gut microbes by characterizing the complex composition of microbial proteins (Stamboulian et al., 2021).

Enzyme-linked immunosorbent assay (ELISA), real-time fluorescent quantitative PCR, Western blot, and immunohistochemistry methods can be used to detect biochemical markers related to metabolic diseases (e.g., fasting blood glucose, insulin, total cholesterol, triglycerides, alanine aminotransferase, and aspartate aminotransferase) in addition to intestinal barrier-related markers (e.g., occludin, ZO-1, and claudin-1) and inflammation-related markers (e.g., LPS, IL-1β, IL-6, and TNF-α). Metabolomics can be used to determine changes in microbial metabolites after drug administration (van Treuren and Dodd, 2020). Metabolomics analysis technology includes gas chromatography-mass spectrometry (GC-MS) and high-performance liquid chromatography-triple quadrupole mass spectrometry (HPLC-QqQ-MS), which can be used to efficiently perform qualitative and/or quantitative analysis of gut microbial metabolites. For example, GC-MS technology can accurately measure the levels of SCFAs produced by gut microbial fermentation, while HPLC-QqQ-MS technology can accurately determine the concentration of BAs. Additionally, mass spectrometry imaging (MSI) techniques, including matrix-assisted laser desorption/ionization MSI, desorption electrospray ionization MSI, and nanostructure-initiator MSI, are becoming attractive tools for the spatial visualization of microbial metabolites in tissues (Luan et al., 2017; Bauermeister et al., 2022). These MSI techniques help us understand the effects of herbal medicines on gut microbial metabolites in two or three dimensions. Additionally, bioinformatic methods can be used to study the correlation between the pharmacodynamic effects of herbal medicines and changes in gut microbes and their metabolites. In addition to antibiotic interventions, fecal microbiota transplantation (FMT) can also be used to identify the critical role of the gut microbiota in the herbal treatment of metabolic diseases.

#### 3.3.2 Methodology to study the biotransformation of herbal ingredients by the gut microbiota

After oral administration, herbal ingredients can be metabolized by gut microbes, and their metabolites are then absorbed into the circulation, producing pharmacological activity (van Duynhoven et al., 2011; Zierer et al., 2018). To determine whether the gut microbiota is involved in the metabolism of herbal ingredients, three experimental groups were created: a control group, a herbal medicine intervention group, and a herbal medicine intervention plus antibiotic group. After the experiment, animal biological samples, including feces or intestinal contents and serum, need to be collected. Feces or intestinal contents can be used to determine herbal metabolites after gut microbial transformation, while serum can be used to determine the absorption of these metabolites and whether bioavailability is improved. Due to the low levels of these metabolites in biological samples, high-sensitivity analytical instruments are needed for their detection. Ultra-high-performance liquid chromatography coupled with Orbitrap mass spectrometry (UPLC-Orbitrap-MS) and HPLC-QqQ-MS have been shown to accurately detect and identify metabolites of herbal phytochemicals in rat intestinal contents and serum samples (Du et al., 2020; Du et al., 2021). Furthermore, additional experimental validation is needed to determine whether the metabolites after gut microbial transformation have a better biological activity or lower toxicity than their precursors. In short, medium-pressure preparation liquid chromatography and high-speed countercurrent chromatography can be used for the targeted separation of specific metabolites. Then, cellular or animal experiments can be performed to compare the activity or toxicity of these metabolites with their precursors.

## 4 Conclusion and perspectives

Gut microbes and their metabolites have recently been implicated to be involved in the pathogenesis of metabolic diseases (Fan and Pederson, 2021; Du et al., 2022). Consequently, the gut microbiota may be a potential target for herbal treatments of metabolic diseases. Several articles have summarized the association between gut microbiota and herbal medicines (Xu et al., 2017; An et al., 2019; Zhao et al., 2020). However, the critical role of gut microbiota in the herbal treatment of metabolic diseases has not been fully described. Therefore, this review provides a comprehensive and up-to-date summary of the relationship between herbal medicines and gut microbiota in metabolic diseases.

There is accumulating evidence indicating the significant contribution of the gut microbiota to the herbal treatment of metabolic diseases. On the one hand, herbal medicines can improve metabolic diseases by increasing beneficial bacteria (e.g., *Akkermansia* and *Blautia*), reducing harmful bacteria (e.g., *Escherichia*–*Shigella*), protecting the intestinal barrier to relieve inflammation, and regulating gut microbial metabolites. On the other hand, gut microbes can metabolize and transform herbal compounds via glycoside hydrolase, oxidase, and reductase. These transformations may make herbs more bioavailable and bioactive or less toxic and thus benefit the treatment of metabolic diseases.

Despite advances in the research of herbal medicines and their effects on the gut microbiota, current studies are limited as they mostly rely on 16S rRNA sequencing technology to detect gut microbes, which has resulted in observations of the effects of herbal medicines on the gut microbiota at the family or genus level. To further explore the effects of herbal medicines on the gut microbiota, metagenomics and/or culturomics should be employed to identify key bacterial species that may be responsible for therapeutic effects. Moreover, current studies on the metabolism of herbal compounds by gut microbes are limited to one or a small class of components, and further analysis of more chemical compositions is needed to gain a better understanding of the overall impact of gut microbiota on herbal medicines. Overall, the recent research progress in the interaction between herbal medicines and gut microbiota in metabolic diseases is encouraging. A comprehensive understanding of these interactions will help reveal the therapeutic mechanisms of herbal medicines.

## References

[B1] An X. Bao Q. Di S. Zhao Y. Zhao S. Zhang H. (2019). The interaction between the gut Microbiota and herbal medicines. Biomed. Pharmacother. 118, 109252. 10.1016/j.biopha.2019.109252 31545247

[B2] Ansari A. Bose S. Yadav M. K. Wang J. H. Song Y. K. Ko S. G. (2016). CST, an herbal formula, exerts anti-obesity effects through brain-gut-adipose tissue axis modulation in high-fat diet fed mice. Molecules 21 (11), 1522. 10.3390/molecules21111522 27845741 PMC6274029

[B3] Bai J. Zhu Y. Dong Y. (2016). Response of gut microbiota and inflammatory status to bitter melon (*Momordica charantia* L.) in high fat diet induced obese rats. J. *Ethnopharmacol* . 194, 717–726. 10.1016/j.jep.2016.10.043 27751827

[B4] Bauermeister A. Mannochio-Russo H. Costa-Lotufo L. V. Jarmusch A. K. Dorrestein P. C. (2022). Mass spectrometry-based metabolomics in microbiome investigations. Nat. Rev. Microbiol. 20 (3), 143–160. 10.1038/s41579-021-00621-9 34552265 PMC9578303

[B5] Cai J. W. Rimal B. Jiang C. T. Chiang J. Y. L. Patterson A. D. (2022). Bile acid metabolism and signaling, the microbiota, and metabolic disease. Pharmacol. Ther. 237, 108238. 10.1016/j.pharmthera.2022.108238 35792223

[B6] Canfora E. E. Jocken J. W. Blaak E. E. (2015). Short-chain fatty acids in control of body weight and insulin sensitivity. Nat. Rev. Endocrinol. 11 (10), 577–591. 10.1038/nrendo.2015.128 26260141

[B7] Cao Y. Yao G. Sheng Y. Yang L. Wang Z. Yang Z. (2019). JinQi Jiangtang tablet regulates gut microbiota and improve insulin sensitivity in type 2 diabetes mice. J. Diabetes Res. 2019, 1872134. 10.1155/2019/1872134 30733971 PMC6348821

[B8] Carnevale R. Pastori D. Nocella C. Cammisotto V. Bartimoccia S. Novo M. (2019). Gut-derived lipopolysaccharides increase post-prandial oxidative stress via Nox2 activation in patients with impaired fasting glucose tolerance: Effect of extra-virgin olive oil. Eur. J. Nutr. 58 (2), 843–851. 10.1007/s00394-018-1718-x 29766292

[B9] Chang C. J. Lin C. S. Lu C. C. Martel J. Ko Y. F. Ojcius D. M. (2015). *Ganoderma lucidum* reduces obesity in mice by modulating the composition of the gut microbiota. Nat. Commun. 6, 7489. 10.1038/ncomms8489 26102296 PMC4557287

[B10] Chang C. J. Lin T. L. Tsai Y. L. Wu T. R. Lai H. C. Lu C. C. (2019). Next generation probiotics in disease amelioration. J. Food Drug Anal. 27, 615–622. 10.1016/j.jfda.2018.12.011 31324278 PMC9307044

[B11] Chen F. Wen Q. Jiang J. Li H. L. Tan Y. F. Li Y. H. (2015). Could the gut microbiota reconcile the oral bioavailability conundrum of traditional herbs. J. Ethnopharmacol. 179, 253–264. 10.1016/j.jep.2015.12.031 26723469

[B12] Chen M. Liao Z. Lu B. Wang M. Lin L. Zhang S. (2018). Huang-Lian-Jie-Du-Decoction ameliorates hyperglycemia and insulin resistant in association with gut microbiota modulation. Front. Microbiol. 9, 2380. 10.3389/fmicb.2018.02380 30349514 PMC6186778

[B13] Chen P. Guo Z. I. Chen F. C. Wu Y. Zhou B. H. (2022). Recent advances and perspectives on the health benefits of urolithin B, a bioactive natural product derived from ellagitannins. Front. Pharmacol. 13, 917266. 10.3389/fphar.2022.917266 35814202 PMC9257173

[B14] Chen Y. Tang Z. Huang Z. Zhou W. Li Z. Li X. (2017). The prevalence of gout in mainland China from 2000 to 2016: A systematic review and meta-analysis. J. Public Health 25 (5), 521–529. 10.1007/s10389-017-0812-5

[B15] Christophersen C. T. Morrison M. Conlon M. A. (2011). Overestimation of the abundance of sulfate-reducing bacteria in human feces by quantitative PCR targeting the *Desulfovibrio* 16S rRNA gene. Appl. Environ. Microbiol. 77 (10), 3544–3546. 10.1128/AEM.02851-10 21460115 PMC3126453

[B16] Cui H. X. Zhang L. S. Luo Y. Yuan K. Huang Z. Y. Guo Y. (2019). A purified anthraquinone-glycoside preparation from Rhubarb ameliorates type 2 diabetes mellitus by modulating the gut microbiota and reducing inflammation. Front. Microbiol. 10, 1423. 10.3389/fmicb.2019.01423 31293553 PMC6603233

[B17] De Vadder F. Kovatcheva-Datchary P. Goncalves D. Vinera J. Zitoun C. Duchampt A. (2014). Microbiota-generated metabolites promote metabolic benefits via gut-brain neural circuits. Cell 156 (1-2), 84–96. 10.2337/db14-1213 24412651

[B18] den Besten G. D. Bleeker A. Gerding A. van Eunen K. Havinga R. van Dijk T. H. (2015). Short-chain fatty acids protect against high-fat diet–induced obesity via a PPARγ-dependent switch from lipogenesis to fat oxidation. Diabetes 64, 2398–2408. 10.2337/db14-1213 25695945

[B19] Du H. Xu T. Yi H. Xu X. M. Zhao C. C. Ge Y. M. (2021). Effect of gut microbiota on the metabolism of chemical constituents of *Berberis kansuensis* extract based on UHPLC-Orbitrap-MS technique. Planta Med. 88, 933–949. 10.1055/a-1617-9489 34521131

[B20] Du H. Xu X. M. Xu T. Li Q. Zhao C. C. Yi H. (2020). Effects of gut microbiota on five absorbed components of *Berberis kansuensis* in rat serum by HPLC-QqQ-MS. China J. Chin. Materia Medica 45, 418–424. 10.19540/j.cnki.cjcmm.20190830.203 32237327

[B21] Du L. Li Q. Yi H. Kuang T. Tang Y. Fan G. (2022). Gut microbiota-derived metabolites as key actors in type 2 diabetes mellitus. Biomed. Pharmacother. 149, 112839. 10.1016/j.biopha.2022.112839 35325852

[B22] Eckel R. H. Grundy S. M. Zimmet P. Z. (2005). The metabolic syndrome. Lancet 365 (9468), 1415–1428. 10.1016/s0140-6736(05)66378-7 15836891

[B23] Espin J. C. González-Barrio R. Cerdá B. López-bote C. Rey A. Tomás-Barberán F. A. (2007). Iberian pig as a model to clarify obscure points in the bioavailability and metabolism of ellagitannins in humans. J. Agric. Food Chem. 55, 10476–10485. 10.1021/jf0723864 17990850

[B24] Espin J. C. Larrosa M. Garcia-Conesa M. T. Tomas-Barberan F. (2013). Biological significance of urolithins, the gut microbial ellagic acid-derived metabolites: The evidence so far. Evid. Based Complement. Altern. Med. 2013, 270418. 10.1155/2013/270418 PMC3679724 23781257

[B25] Everard A. Belzer C. Geurts L. Ouwerkerk J. P. Druart C. Bindels L. B. (2013). Cross-talk between *Akkermansia muciniphila* and intestinal epithelium controls diet-induced obesity. Proc. Natl. Acad. Sci. U. S. A. 110 (22), 9066–9071. 10.1073/pnas.1219451110 23671105 PMC3670398

[B26] Fan Y. Pedersen O. (2021). Gut microbiota in human metabolic health and disease. Nat. Rev. Microbiol. 19, 55–71. 10.1038/s41579-020-0433-9 32887946

[B27] Fang P. H. Yu M. Shi M. Y. Bo P. Gu X. W. Zhang Z. W. (2020). Baicalin and its aglycone: A novel approach for treatment of metabolic disorders. *Pharmacol. Re*p. 72, 13–23. 10.1007/s43440-019-00024-x 32016847

[B28] Feng R. Shou J. W. Zhao Z. X. He C. Y. Ma C. Huang M. (2015). Transforming berberine into its intestine-absorbable form by the gut microbiota. Sci. Rep. 5, 12155. 10.1038/srep12155 26174047 PMC4502414

[B29] Feng W. W. Ao H. Peng C. Yan D. (2019). Gut microbiota, a new frontier to understand traditional Chinese medicines. Pharmacol. Res. 142, 176–191. 10.1016/j.phrs.2019.02.024 30818043

[B30] Gao K. Yang R. Zhang J. Wang Z. Jia C. Zhang F. (2018). Effects of Qijian mixture on type 2 diabetes assessed by metabonomics, gut microbiota and network pharmacology. Pharmacol. Res. 130, 93–109. 10.1016/j.phrs.2018.01.011 29391233

[B31] Guerville M. Leroy A. Sinquin A. Laugerette F. Michalski M. C. Boudry G. (2017). Western-diet consumption induces alteration of barrier function mechanisms in the ileum that correlates with metabolic endotoxemia in rats. Am. J. Physiol. Endocrinol. Metab. 313 (2), E107–E120. 10.1152/ajpendo.00372.2016 28400412

[B32] Han R. Qiu H. Zhong J. Zheng N. Li B. Hong Y. (2021). Si Miao formula attenuates non-alcoholic fatty liver disease by modulating hepatic lipid metabolism and gut microbiota. Phytomedicine 85, 153544. 10.1016/j.phymed.2021.153544 33773192

[B33] Hossain Z. Hirata T. (2008). Molecular mechanism of intestinal permeability: Interaction at tight junctions. Mol. Biosyst. 4, 1181–1185. 10.1039/b800402a 19396381

[B117] Hotamisligil G. S. (2006). Inflammation and metabolic disorders. Nature 444 (7121), 860–867. 10.1038/nature05485 17167474

[B34] Huang J. F. Zhao Q. Dai M. Y. Xiao X. R. Li F. Zhu W. F. (2020). Gut microbiota protects from triptolide-induced hepatotoxicity: Key role of propionate and its downstream signalling events. Pharmacol. Res. 155, 104752. 10.1016/j.phrs.2020.104752 32169656

[B35] Huang R. S. Guo F. Li Y. P. Liang Y. Li G. B. Fu P. (2021). Activation of AMPK by triptolide alleviates nonalcoholic fatty liver disease by improving hepatic lipid metabolism, inflammation, and fibrosis. Phytomedicine 92, 153739. 10.1016/j.phymed.2021.153739 34592488

[B36] Javdan B. Lopez J. G. Chankhamjon P. Lee Y. J. Hull R. Wu Q. (2020). Personalized mapping of drug metabolism by the human gut microbiome. Cell 181 (7), 1661–1679. 10.1016/j.cell.2020.05.001 32526207 PMC8591631

[B37] Jia N. Lin X. Ma S. Ge S. Mu S. Yang C. (2018a). Amelioration of hepatic steatosis is associated with modulation of gut microbiota and suppression of hepatic miR-34a in *Gynostemma pentaphylla* (Thunb.) Makino treated mice. *Nutr. Metab*. (Lond) 15, 86. 10.1186/s12986-018-0323-6 30555521 PMC6282400

[B38] Jia W. Xie G. Jia W. (2018b). Bile acid-microbiota crosstalk in gastrointestinal inflammation and carcinogenesis. Nat. Rev. Gastroenterol. Hepatol. 15, 111–128. 10.1038/nrgastro.2017.119 29018272 PMC5899973

[B39] Jiang S. Ren D. Li J. Yuan G. Li H. Xu G. (2014). Effects of compound K on hyperglycemia and insulin resistance in rats with type 2 diabetes mellitus. Fitoterapia 95, 58–64. 10.1016/j.fitote.2014.02.017 24613802

[B40] Joh E. H. Lee I. A. Jung I. H. Kim D. H. (2011). Ginsenoside Rb1 and its metabolite compound K inhibit IRAK-1 activation--the key step of inflammation. *Biochem. Pharmaco*l. 82, 278–286. 10.1016/j.bcp.2011.05.003 21600888

[B41] Jovel J. Patterson J. Wang W. W. Hotte N. O’Keefe S. Mitchel T. (2016). Characterization of the gut microbiome using 16S or shotgun metagenomics. Front. Microbiol. 7, 459. 10.3389/fmicb.2016.00459 27148170 PMC4837688

[B42] Kawata Y. Cho-mei M. A. Meselhy M. R. Nakamura N. Wang H. (1999). DSpace at University of Toyama: Conversion of aconitine lipoaconitine by human intestinal bacteria and their antinociceptive effects in mice. Journal of traditional medicines. Available at: http://ci.nii.ac.jp/naid/110002536540.

[B43] Khana T. Kim H. G. Choi J. H. Park B. H. Do M. T. Kang M. J. (2012). Protective role of intestinal bacterial metabolism against baicalin-induced toxicity in HepG2 cell cultures. J. Toxicol. Sci. 37, 363–371. 10.2131/jts.37.363 22467027

[B44] Koh A. De Vadder F. Kovatcheva-Datchary P. Backhed F. (2016). From dietary fiber to host physiology: Short-chain fatty acids as key bacterial metabolites. Cell 165 (6), 1332–1345. 10.1016/j.cell.2016.05.041 27259147

[B45] Kondo T. Kishi M. Fushimi T. Kaga T. (2009). Acetic acid upregulates the expression of genes for fatty acid oxidation enzymes in liver to suppress body fat accumulation. J. Agric. Food Chem. 57 (13), 5982–5986. 10.1021/jf900470c 19469536

[B46] Larraufie P. Martin-Gallausiaux C. Lapaque N. Dore J. Gribble F. M. Reimann F. (2018). SCFAs strongly stimulate PYY production in human enteroendocrine cells. Sci. Rep. 8 (1), 74. 10.1038/s41598-017-18259-0 29311617 PMC5758799

[B47] Lee H. U. Bae E. A. Han M. J. Kim N. J. Kim D. H. (2005). Hepatoprotective effect of ginsenoside Rb1 and compound K on tert-butyl hydroperoxide-induced liver injury. Liver Int. 25, 1069–1073. 10.1111/j.1478-3231.2005.01068.x 16162168

[B48] Lei S. S. Li B. Chen Y. H. He X. Wang Y. Z. Yu H. H. (2019). *Dendrobii Officinalis*, a traditional Chinese edible and officinal plant, accelerates liver recovery by regulating the gut-liver axis in NAFLD mice. J. Funct. Foods 61, 103458. 10.1016/j.jff.2019.103458

[B49] Leng J. Huang F. Hai Y. Tian H. Liu W. Fang Y. (2020). Amelioration of non-alcoholic steatohepatitis by Qushi Huayu decoction is associated with inhibition of the intestinal mitogen-activated protein kinase pathway. Phytomedicine 66, 153135. 10.1016/j.phymed.2019.153135 31790895

[B50] Li L. Xu X. Lu X. Zhang Y. Lin W. Xu R. (2019a). Effects of *Alisma orientale* on the diversity of gut microbiota in rats fed on high-fat and high-sugar diet. Front Chin. J. Microecology 31 (4). 10.13381/j.cnki.cjm.201904005

[B51] Li N. Wu Y. Duan J. Zheng X. Yao K. (2021b). Explore effect of Jieyu Qutan Huazhuo prescription on gut-liver axis of rats with high-fat diet based on 16S rDNA sequencing. Chin. J. Exp. Traditional Med. Formulae 27 (9), 77–85. 10.13422/j.cnki.syfjx.20210327

[B52] Li Q. Hu J. Nie Q. Chang X. Fang Q. Xie J. (2021c). Hypoglycemic mechanism of polysaccharide from *Cyclocarya paliurus* leaves in type 2 diabetic rats by gut microbiota and host metabolism alteration. Sci. China Life Sci. 64 (1), 117–132. 10.1007/s11427-019-1647-6 32562054

[B53] Li W. Zhang M. Gu J. Meng Z. J. Zhao L. C. Zheng Y. N. (2012). Hypoglycemic effect of protopanaxadiol-type ginsenosides and compound K on Type 2 Diabetes mice induced by High-Fat Diet combining with Streptozotocin via suppression of hepatic gluconeogenesis. Fitoterapia 83, 192–198. 10.1016/j.fitote.2011.10.011 22056666

[B54] Li X. J. Jiang Z. Z. Zhang L. Y. (2014). Triptolide: Progress on research in pharmacodynamics and toxicology. J. Ethnopharmacol. 155, 67–79. 10.1016/j.jep.2014.06.006 24933225

[B55] Li X. Zhao W. Xiao M. Yu L. Chen Q. Hu X. (2022). *Penthorum chinense* Pursh. extract attenuates non-alcholic fatty liver disease by regulating gut microbiota and bile acid metabolism in mice. J. Ethnopharmacol. 294, 115333. 10.1016/j.jep.2022.115333 35500802

[B56] Liang S. Zhang Y. Deng Y. He Y. Liang Y. Liang Z. (2018). The potential effect of Chinese herbal formula hongqijiangzhi fang in improving NAFLD: Focusing on NLRP3 inflammasome and gut microbiota. Evid. Based Complement. Altern. Med. 2018, 5378961. 10.1155/2018/5378961 PMC5841032 29675053

[B57] Liao J. Xie X. Gao J. Zhang Z. Qv F. Cui H. (2020). Jian-Gan-Xiao-Zhi decoction ameliorates nonalcoholic fatty liver disease through modulating gut microbiota, decreasing gut permeability, and alleviating liver inflammation. Res. Square. 10.21203/rs.3.rs-122886/v1

[B58] Liu M. T. Huang Y. J. Zhang T. Y. Tan L. B. Lu X. F. Qin J. (2019). Lingguizhugan decoction attenuates diet-induced obesity and hepatosteatosis via gut microbiota. World J. Gastroenterol. 25 (27), 3590–3606. 10.3748/wjg.v25.i27.3590 31367159 PMC6658390

[B59] Liu X. M. Mao B. Y. Gu J. Y. Wu J. Y. Chen W. Wang G. (2021). *Blautia*-a new functional genus with potential probiotic properties. Gut Microbes 13, 1–21. 10.1080/19490976.2021.1875796 PMC7872077 33525961

[B60] Liu Y. Hou Y. Wang G. Zheng X. Hao H. (2020). Gut microbial metabolites of aromatic amino acids as signals in host-microbe interplay. Trends Endocrinol. Metab. 31 (11), 818–834. 10.1016/j.tem.2020.02.012 32284282

[B61] Liu Z. H. Maszenan A. M. Liu Y. Jern Ng W. (2014). A brief review on possible approaches towards controlling sulfate-reducing bacteria (SRB) in wastewater treatment systems. Desalination Water Treat. 53 (10), 2799–2807. 10.1080/19443994.2014.943023

[B62] Lu Y. Wan H. Wu Y. Yang J. Yu L. He Y. (2022). Naoxintong capsule alternates gut microbiota and prevents hyperlipidemia in high-fat-diet fed rats. Front. Pharmacol. 13, 843409. 10.3389/fphar.2022.843409 35387330 PMC8978017

[B63] Luan H. M. Wang X. Cai Z. W. (2017). Mass spectrometry‐based metabolomics: Targeting the crosstalk between gut microbiota and brain in neurodegenerative disorders. Mass Spectrom. Rev. 38, 22–33. 10.1002/mas.21553 29130504

[B64] Mandaliya D. K. Seshadri S. (2019). Short chain fatty acids, pancreatic dysfunction, and type 2 diabetes. Pancreatology 19 (2), 280–284. 10.1016/j.pan.2019.01.021 30713129

[B65] McGuckin M. A. Eri R. Simms L. A. Florin T. H. Radford-Smith G. (2009). Intestinal barrier dysfunction in inflammatory bowel diseases. Inflamm. Bowel Dis. 15, 100–113. 10.1002/ibd.20539 18623167

[B66] Mehta N. N. McGillicuddy F. C. Anderson P. D. Christine C. Hinkle C. C. Shah R. (2010). Experimental endotoxemia induces adipose inflammation and insulin resistance in humans. Diabetes 59, 172–181. 10.2337/db09-0367 19794059 PMC2797919

[B67] Meijers B. Farre R. Dejongh S. Vicario M. Evenepoel P. (2018). Intestinal barrier function in chronic kidney disease. Toxins (Basel) 10, 298. 10.3390/toxins10070298 30029474 PMC6071212

[B68] Mohammad S. Thiemermann C. (2021). Role of metabolic endotoxemia in systemic inflammation and potential interventions. Front. Immunol. 11, 594150. 10.3389/fimmu.2020.594150 33505393 PMC7829348

[B69] Morin N. Visentin V. Calise D. Marti L. Zorzano A. Testar X. (2002). Tyramine stimulates glucose uptake in insulin-sensitive tissues *in vitro* and *in vivo* via its oxidation by amine oxidases. J. Pharmacol. Exp. Ther. 303 (3), 1238–1247. 10.1124/jpet.102.040592 12438548

[B70] Neis E. P. J. G. Dejong C. H. C. Rensen S. S. (2015). The role of microbial amino acid metabolism in host metabolism. Nutrients 7, 2930–2946. 10.3390/nu7042930 25894657 PMC4425181

[B71] Nie Q. Hu J. Gao H. Fan L. Chen H. Nie S. (2019). Polysaccharide from *Plantago asiatica* L. attenuates hyperglycemia, hyperlipidemia and affects colon microbiota in type 2 diabetic rats. Food Hydrocoll. 86, 34–42. 10.1016/j.foodhyd.2017.12.026

[B72] Ohira H. Fujioka Y. Katagiri C. Mamoto R. Aoyama-Ishikawa M. Amako K. (2013). Butyrate attenuates inflammation and lipolysis generated by the interaction of adipocytes and macrophages. J. Atheroscler. Thromb. 20 (5), 425–442. 10.5551/jat.15065 23470566

[B73] Okazaki F. Zang L. Q. Nakayama H. Chen Z. Gao Z. J. Chiba H. (2019). Microbiome alteration in type 2 diabetes mellitus model of zebrafish. Sci. Rep. 9, 867. 10.1038/s41598-018-37242-x 30696861 PMC6351536

[B74] Paone P. Cani P. D. (2020). Mucus barrier, mucins and gut microbiota: The expected slimy partners. Gut 69, 2232–2243. 10.1136/gutjnl-2020-322260 32917747 PMC7677487

[B75] Pols T. W. Auwerx J. Schoonjans K. (2010). Targeting the TGR5-GLP-1 pathway to combat type 2 diabetes and non-alcoholic fatty liver disease. Gastroenterol. Clin. Biol. 34 (4-5), 270–273. 10.1016/j.gcb.2010.03.009 20444564

[B76] Rahimi V. B. Askari V. R. Hosseinzadeh H. (2021). Promising influences of *Scutellaria baicalensis* and its two active constituents, baicalin, and baicalein, against metabolic syndrome: A review. Phytotherapy Res. 35, 3558–3574. 10.1002/ptr.7046 33590943

[B77] Raimundo A. F. Ferreira S. Tomas-Barberan F. A. Santos C. N. Menezes R. (2021). Urolithins: Diet-derived bioavailable metabolites to tackle diabetes. Nutrients 13, 4285. 10.3390/nu13124285 34959837 PMC8705976

[B78] Shao J. Liu Y. Wang H. Luo Y. Chen L. (2020). An integrated fecal microbiome and metabolomics in T2DM rats reveal antidiabetes effects from host-microbial metabolic axis of EtOAc extract from *Sophora flavescens* . Oxid. Med. Cell Longev. 2020, 1805418. 10.1155/2020/1805418 32566075 PMC7273480

[B79] Shao X. T. Li N. Zhan J. Z. Sun H. An L. P. Du P. G. (2015). Protective effect of compound K on diabetic rats. Nat. Prod. Commun. 10, 1934578X1501000–245. 10.1177/1934578x1501000206 25920251

[B80] Shin N. R. Whon T. W. Bae J. W. (2015). Proteobacteria: Microbial signature of dysbiosis in gut microbiota. Trends Biotechnol. 33 (9), 496–503. 10.1016/j.tibtech.2015.06.011 26210164

[B81] Stamboulian M. Canderan J. Ye Y. (2021). Metaproteomics as a tool for studying the protein landscape of human-gut bacterial species. Cold Spring Harb. Lab. 10.1101/2021.09.02.458484 PMC8967034 35302987

[B82] Toney A. M. Fox D. Chaidez V. Ramer-Tait A. E. Chung S. (2021). Immunomodulatory role of urolithin a on metabolic diseases. Biomedicines 9, 192. 10.3390/biomedicines9020192 33671880 PMC7918969

[B83] van Duynhoven J. Vaughan E. E. Jacobs D. M. Kemperman R. A. van Velzen E. J. Gross G. (2011). Metabolic fate of polyphenols in the human superorganism. Proc. Natl. Acad. Sci. U. S. A. 108 (1), 4531–4538. 10.1073/pnas.1000098107 20615997 PMC3063601

[B84] van Treuren W. Dodd D. (2020). Microbial contribution to the human metabolome: Implications for health and disease. Annu. Rev. Pathol. 15, 345–369. 10.1146/annurev-pathol-020117-043559 31622559 PMC7678725

[B85] Visentin V. Marq P. Bour S. Subra C. Prévot D. Morin N. (2003). Effect of prolonged treatment with tyramine on glucose tolerance in streptozotocin-induced diabetic rats. J. Physiol. Biochem. 59 (3), 225–232. 10.1007/BF03179919 15000454

[B86] Wahlstrom A. Sayin S. I. Marschall H. U. Backhed F. (2016). Intestinal crosstalk between bile acids and microbiota and its impact on host metabolism. Cell Metab. 24 (1), 41–50. 10.1016/j.cmet.2016.05.005 27320064

[B87] Wang R. R. Zhang L. F. Chen L. P. Wang J. Y. Zhang L. Xu Y. S. (2021). Structural and functional modulation of gut microbiota by Jiangzhi granules during the amelioration of nonalcoholic fatty liver disease. Oxid. Med. Cell Longev. 2021, 2234695. 10.1155/2021/2234695 34966475 PMC8712166

[B88] Wei X. Tao J. Xiao S. Jiang S. Shang E. Zhu Z. (2018). Xiexin Tang improves the symptom of type 2 diabetic rats by modulation of the gut microbiota. Sci. Rep. 8 (1), 3685. 10.1038/s41598-018-22094-2 29487347 PMC5829262

[B89] Wen X. Lou Y. Song S. He Z. Chen J. Xie Z. (2021). Qu-Zhuo-Tong-Bi decoction alleviates gouty arthritis by regulating butyrate-producing bacteria in mice. Front. Pharmacol. 11, 610556. 10.3389/fphar.2020.610556 33603667 PMC7884811

[B90] Whang A. Nagpal R. Yadav H. (2019). Bi-directional drug-microbiome interactions of anti-diabetics. Ebiomedicine 39, 591–602. 10.1016/j.ebiom.2018.11.046 30553752 PMC6354569

[B91] Wu J. Wang K. Wang X. Pang Y. Jiang C. (2021). The role of the gut microbiome and its metabolites in metabolic diseases. Protein Cell 12, 360–373. 10.1007/s13238-020-00814-7 33346905 PMC8106557

[B92] Xiao S. Liu C. Chen M. Zou J. Zhang Z. Cui X. (2020). *Scutellariae radix* and *Coptidis rhizoma* ameliorate glycolipid metabolism of type 2 diabetic rats by modulating gut microbiota and its metabolites. Appl. Microbiol. Biotechnol. 104 (1), 303–317. 10.1007/s00253-019-10174-w 31758238

[B93] Xiong Y. Miyamoto N. Shibata K. Valasek M. A. Motoike T. Kedzierski R. M. (2004). Short-chain fatty acids stimulate leptin production in adipocytes through the G protein-coupled receptor GPR41. PNAS 101 (4), 1045–1050. 10.1073/pnas.2637002100 14722361 PMC327148

[B94] Xu J. Chen H. B. Li S. L. (2017). Understanding the molecular mechanisms of the interplay between herbal medicines and gut microbiota. Med. Res. Rev. 37 (5), 1140–1185. 10.1002/med.21431 28052344

[B95] Xu J. Lian F. Zhao L. Zhao Y. Chen X. Zhang X. (2015). Structural modulation of gut microbiota during alleviation of type 2 diabetes with a Chinese herbal formula. ISME J. 9 (3), 552–562. 10.1038/ismej.2014.177 25279787 PMC4331591

[B96] Xu T. Ge Y. Du H. Li Q. Xu X. Yi H. (2021). Berberis kansuensis extract alleviates type 2 diabetes in rats by regulating gut microbiota composition. J. Ethnopharmacol. 273, 113995. 10.1016/j.jep.2021.113995 33675912

[B97] Xu X. Gao Z. Yang F. Yang Y. Chen L. Han L. (2020). Antidiabetic effects of Gegen Qinlian decoction via the gut microbiota are attributable to its key ingredient berberine. Genomics Proteomics Bioinforma. 18 (6), 721–736. 10.1016/j.gpb.2019.09.007 PMC8377040 33359679

[B98] Yan J. Sheng L. L. Li H. K. (2021). *Akkermansia muciniphila*: Is it the holy grail for ameliorating metabolic diseases. Gut Microbes 13, 1984104. 10.1080/19490976.2021.1984104 34674606 PMC8726741

[B99] Yan Z. Wu H. Zhou H. Chen S. He Y. Zhang W. (2020). Integrated metabolomics and gut microbiome to the effects and mechanisms of naoxintong capsule on type 2 diabetes in rats. Sci. Rep. 10 (1), 10829. 10.1038/s41598-020-67362-2 32616735 PMC7331749

[B100] Yang L. Chen K. Luo D. Guo J. (2022a). Efficacy and mechanism of Tianhuang formula in regulating lipid metabolism disorders in senile mice based on gut microbiota-T-β-MCA-FXR axis. Pharmacol. Clin. Chin. Materia Medica, 1–8.

[B101] Yang Y. Cao S. Xu W. Zang C. Zhang F. Xie Y. (2022b). Dual modulation of gut bacteria and fungi manifests the gut-based anti-hyperlipidemic effect of *Coptidis Rhizoma* . Biomed. Pharmacother. 153, 113542. 10.1016/j.biopha.2022.113542 36076619

[B102] Younossi Z. Anstee Q. M. Marietti M. Hardy T. Henry L. Eslam M. (2018). Global burden of NAFLD and NASH: Trends, predictions, risk factors and prevention. Nat. Rev. Gastroenterol. Hepatol. 15 (1), 11–20. 10.1038/nrgastro.2017.109 28930295

[B103] Yu L. Song P. Zhu Q. Li Y. Jia S. Zhang S. (2022). The dietary branched-chain amino acids transition and risk of type 2 diabetes among Chinese adults from 1997 to 2015: Based on seven cross-sectional studies and a prospective cohort study. Front. Nutr. 9, 881847. 10.3389/fnut.2022.881847 35677550 PMC9168595

[B104] Yue S. J. Liu J. Wang A. T. Meng X. T. Yang Z. R. Peng C. (2019). Berberine alleviates insulin resistance by reducing peripheral branched-chain amino acids. Am. J. Physiol. Endocrinol. Metab. 316, E73–E85. 10.1152/ajpendo.00256.2018 30422704

[B105] Zhang C. Liu J. He X. Sheng Y. Yang C. Li H. (2019). *Caulis spatholobi* ameliorates obesity through activating Brown adipose tissue and modulating the composition of gut microbiota. Int. J. Mol. Sci. 20 (20), 5150. 10.3390/ijms20205150 31627416 PMC6829277

[B106] Zhang L. Wang Y. Wu F. Wang X. Feng Y. Wang Y. (2022a). MDG, an *Ophiopogon japonicus* polysaccharide, inhibits non-alcoholic fatty liver disease by regulating the abundance of *Akkermansia muciniphila* . Int. J. Biol. Macromol. 196, 23–34. 10.1016/j.ijbiomac.2021.12.036 34920070

[B107] Zhang M. Peng C. S. Li X. B. (2015). *In vivo* and *in vitro* metabolites from the main diester and monoester diterpenoid alkaloids in a traditional Chinese herb, the aconitum species. Evid. Based Complement. Altern. Med. 2015, 252434. 10.1155/2015/252434 PMC4332761 25705235

[B108] Zhang X. Wang H. Xie C. Hu Z. Zhang Y. Peng S. (2022b). Shenqi compound ameliorates type-2 diabetes mellitus by modulating the gut microbiota and metabolites. J. Chromatogr. B Anal. Technol. Biomed. Life Sci. 1194, 123189. 10.1016/j.jchromb.2022.123189 35219959

[B109] Zhang Y. Thompson K. Branck T. Yan Y. Nguyen L. Franzosa E. (2021). Metatranscriptomics for the human microbiome and microbial community functional profiling. Annu. Rev. Biomed. data Sci. 4, 279–311. 10.1146/annurev-biodatasci-031121-103035 34465175

[B110] Zhao L. Ma P. Peng Y. Wang M. Peng C. Zhang Y. (2021). Amelioration of hyperglycaemia and hyperlipidaemia by adjusting the interplay between gut microbiota and bile acid metabolism: *Radix Scutellariae* as a case. Phytomedicine 83, 153477. 10.1016/j.phymed.2021.153477 33545549

[B111] Zhao T. Wang Z. Liu Z. Xu Y. (2020). Pivotal role of the interaction between herbal medicines and gut microbiota on disease treatment. Curr. Drug Targets 21, 336–346. 10.2174/1389450121666200324151530 32208116

[B112] Zhao Y. F. Song F. R. Guo X. H. Liu S. Y. (2008). Studies on the biotransformation of aconitine in human intestinal bacteria using soft-ionization mass spectrometry. Chem. J. Chin. Univ. 29 (1), 55–59. 10.1007/978-3-540-77072-5-3

[B113] Zhou M. Shao J. Wu C. Shu L. Dong W. Liu Y. (2019). Targeting BCAA catabolism to treat obesity-associated insulin resistance. Diabetes 68 (9), 1730–1746. 10.2337/db18-0927 31167878 PMC6702639

[B114] Zhou Q. Zhang Y. F. Wang X. X. Yang R. Y. Zhu X. Q. Zhang Y. (2020). Gut bacteria *Akkermansia* is associated with reduced risk of obesity: Evidence from the American. Gut Proj. Nutr. Metabolism 17 (1), 90. 10.1186/s12986-020-00516-1 PMC7583218 33110437

[B115] Zierer J. Jackson M. A. Kastenmuller G. Mangino M. Long T. Telenti A. (2018). The fecal metabolome as a functional readout of the gut microbiome. Nat. Genet. 50 (6), 790–795. 10.1038/s41588-018-0135-7 29808030 PMC6104805

