# Supplementary material for: The interplay between herbal medicines and gut microbiota in metabolic diseases
Source: Front Pharmacol. 2023 Mar 24;14:1105405. doi: 10.3389/fphar.2023.1105405 (PMC10079915; doi:10.3389/fphar.2023.1105405)
Supplement: Supplementary file 1 [file DataSheet1.docx]

**Supplementary Table S1** The composition of herbal preparations.

| Preparation | Drug name | Chinese name | Plant origin | Part used | Dose used |
| --- | --- | --- | --- | --- | --- |
| Naoxintong capsule | *Astragali Radix* | Huangqi | *Astragalus membranaceus* (Fisch.) or  Bge.var.mongholicus (Bge.) Hsiao or  *Astragalus membranaceus* (Fisch.) Bge. | Root | 66 g |
|  | *Paeoniae Radix Rubra* | Chishao | *Paeonia lactiflora* Pall. or  *Paeonia veitchii* Lynch | Root | 27 g |
|  | *Salviae miltiorrhizae Radix et Rhizoma* | Danshen | *Salvia miltiorrhiza* Bge. | Root/ Rhizome | 27 g |
|  | *Angelicae sinensis Radix* | Danggui | *Angelica sinensis* (Oliv.) Diels | Root | 27 g |
|  | *Chuanxiong Rhizoma* | Chuanxiong | *Ligusticum chuanxiong* Hort. | Rhizome | 27 g |
|  | *Persicae Semen* | Taoren | *Prunus persica* (L.) Batsch or  *Prunus davidiana* (Carr.) Franch. | Seed | 27 g |
|  | *Achyranthis bidentatae Radix* | Niuxi | *Achyranthes bidentata* Bl. | Root | 27 g |
|  | *Spatholobi Caulis* | Jixueteng | *Spatholobus suberectus* Dunn | Stem | 20 g |
|  | *Cinnamomi Ranulus* | Guizhi | *Cinnamomum cassia* Presl | Twigs | 20 g |
|  | *Carthami Flos* | Honghua | *Carthamus tinctorius* L. | Flower | 13 g |
|  | *Mori Ramulus* | Sangzhi | *Morus alba* L. | Twigs | 27 g |
|  | *Olibanum* | Ruxiang | *Boswellia carterii* Birdw. or  *Boswellia bhaw-dajiana* Birdw. | Resin | 13 g |
|  | *Myrrha* | Moyao | *Commiphora myrrha* Engl. or  *Commiphora molmol* Engl. | Resin | 13 g |
|  | *Scorpio* | Quanxie | *Buthus martensii* Karsch | Dried body | 13 g |
|  | *Pheretima* | Dilong | *Pheretima aspergillum* (E.Perrier) or  *Pheretima vulgaris* Chen or  *Pheretima guillelmi* (Michaelsen) or  *Pheretima pectinifera* Michaelsen | Dried body | 27 g |
|  | *Hirudo* | Shuizhi | *Whitmania pigra* Whitman or  *Hirudo nipponica* Whitman or  *Whitmania acranulata* Whitman | Dried body | 27 g |
| Xiexin decoction | *Rhei Radix et Rhizome* | Dahuang | *Rheum palmatum* L. or  *Rheum tanguticum* Maxim. ex Bal£. or *Rheum officinale* Baill. | Root/ Rhizome | 10 g |
|  | *Scutellaria Radix* | Huangqin | *Scutellaria baicalensis* Georgi | Root | 5 g |
|  | *Coptidis Rhizoma* | Huanglian | *Coptis chinensis* Franch. or  *Coptis deltoidea* C.Y.Cheng et Hsiao or *Coptis teeta* Wall. | Rhizome | 5 g |
| Huanglian Jiedu decoction | *Coptidis Rhizoma* | Huanglian | *Coptis chinensis* Franch. or  *Coptis deltoidea* C.Y.Cheng et Hsiao or *Coptis teeta* Wall. | Rhizome | 9 g |
|  | *Scutellaria Radix* | Huangqin | *Scutellaria baicalensis* Georgi | Root | 6 g |
|  | *Phellodendri Chinensis Cortex* | Huangbo | *Phellodendron chinense* Schneid. | Cortex | 6 g |
|  | *Gardeniae Fructus* | Zhizi | *Gardenia jasminoides* Ellis | Fruit | 9 g |
| Jinqi Jiangtang tablet | *Coptidis Rhizoma* | Huanglian | *Coptis chinensis* Franch. or  *Coptis deltoidea* C.Y.Cheng et Hsiao or *Coptis teeta* Wall. | Rhizome | 343 g |
|  | *Scutellaria Radix* | Huangqin | *Scutellaria baicalensis* Georgi | Root | 513 g |
|  | *Lonicerae Japonicerae Flos* | Jinyinhua | *Lonicera japonica* Thunb. | Flower | 2058 g |
| Gegen Jiaotai pills | *Puerariae Lobatae Radix* | Gegen | *Pueraria lobata* (Willd.) Ohwi | Root | Unknown |
|  | *Coptidis Rhizoma* | Huanglian | *Coptis chinensis* Franch. or  *Coptis deltoidea* C.Y.Cheng et Hsiao or *Coptis teeta* Wall. | Rhizome |  |
|  | *Cinnamomi Cortex* | Rougui | *Cinnamomum cassia* Presl | Cortex |  |
| Shenqi compound | *Ginseng Radix et Rhizoma* | Renshen | *Panax ginseng* C.A.Mey. | Root/ Rhizome | 15 g |
|  | *Astragali Radix* | Huangqi | *Astragalus membranaceus* (Fisch.) Bge.var.mongholicus (Bge.) Hsiao or  *Astragalus membranaceus* (Fisch.) Bge | Root | 15 g |
|  | *Trichosanthis Radix* | Tianhuafen | *Trichosanthes kirilowii* Maxim. or  *Trichosanthes rosthornii* Harms | Root | 10 g |
|  | *Rehmanniae Radix* | Dihuang | *Rehmannia glutinosa* Libosch. | Earthnut | 10 g |
|  | *Salviae miltiorrhizae Radix et Rhizoma* | Danshen | *Salvia miltiorrhiza* Bge. | Radix/ Rhizome | 10 g |
|  | *Corni Fructus* | Shanzhuyu | *Cornus officinalis* Sieb et Zucc. | Fruit | 10 g |
|  | *Dioscoreae Rhizoma* | Shanyao | *Dioscorea oppositifolia* L. | Root/ Rhizome | 10 g |
|  | *Rhei Radix et Rhizome* | Dahuang | *Rheum palmatum* L. or  *Rheum tanguticum* Maxim. ex Bal£. or  *Rheum officinale* Baill. | Root/ Rhizome | 6 g |
| Gegen Qinlian decoction | *Coptidis Rhizoma* | Huanglian | *Coptis chinensis* Franch. or  *Coptis deltoidea* C.Y.Cheng et Hsiao or  *Coptis teeta* Wall. | Rhizome | Unknown |
|  | *Scutellaria Radix* | Huangqin | *Scutellaria baicalensis* Georgi | Root |  |
|  | *Puerariae Lobatae Radix* | Gegen | *Pueraria lobata* (Willd.) Ohwi | Root |  |
|  | *Anemarrhenae Rhizoma* | Zhimu | *Anemarrhena asphodeloides* Bge. | Rhizome |  |
|  | *Panacis Quinquefolii Radix* | Xiyangshen | *Panax quinquefolium* L. | Root |  |
|  | *Paeoniae Radix Rubra* | Chishao | *Paeonia lactiflora* Pall. or  *Paeonia veitchii* Lynch | Root |  |
|  | *Zingiberis Rhizoma* | Ganjiang | *Zingiber officinale* Rosc. | Rhizome |  |
| Jiangtangjing granules | *Astragali Radix* | Huangqi | *Astragalus membranaceus* (Fisch.) Bge.var.mongholicus (Bge.) Hsiao or  *Astragalus membranaceus* (Fisch.) Bge. | Root | 30 g |
|  | *Puerariae Lobatae Radix* | Gegen | *Pueraria lobata* (Willd.) Ohwi | Root | 30 g |
|  | *Dioscoreae Rhizoma* | Shanyao | *Dioscorea opposita* Thunb | Rhizome | 15 g |
|  | *Polygonati Rhizoma* | Huangjing | *Polygonatum kingianum* Coll.et Hemsl or *Polygonatum sibiricum* Red. or  *Polygonatum cyrtonema* Hua | Rhizome | 15 g |
|  | *Coicis Semen* | Yiyiren | *Coix lacryma-jobi* L.var.ma-yuen (Roman.) Stapf | Seed | 15 g |
|  | *Crataegi Fructus* | Shanzha | *Crataegus pinnatifida* Bge. var. major N. E. Br. or *Crataegus pinnatifida* Bge. | Fruit | 15 g |
|  | *Hirudo* | Shuizhi | *Whitmania pigra* Whitman *or*  *Hirudo nipponica* Whitman or  *Whitmania acranulata* Whitman | Dried body | 5 g |
|  | *Sinapis Semen* | Baijiezi | *Sinapis alba* L. or *Brassica juncea* (L.) Czern. et Coss. | Seed | 5 g |
| Linggui Zhugan formula | *Poria* | Fuling | *Poria cocos* (Schw.) Wolf | Sclerotium | 12 g |
|  | *Cinnamomi Ranulus* | Guizhi | *Cinnamomum cassia* Presl | Twigs | 9 g |
|  | *Atractylodis Macrocephalae Rhizoma* | Baizhu | *Atractylodes macrocephala* Koidz. | Rhizome | 6 g |
|  | *Glycyrrhizae Radix* | Gancao | *Glycyrrhiza uralensis* Fisch. or  *Glycyrrhiza inflata* Bat. or  *Glycyrrhiza glabra* L. | Root/ Rhizome | 6 g |
| Shenlian decoction | *Coptidis Rhizoma* | Huanglian | *Coptis chinensis* Franch. or  *Coptis deltoidea* C.Y.Cheng et Hsiao or  *Coptis teeta* Wall. | Rhizome | 30 g |
|  | *Ginseng Radix et Rhizoma* | Renshen | *Panax ginseng* C.A.Mey. | Root/ Rhizome | 15 g |
| Liuwei Dihuang pills | *Rehmanniae Radix Preparata* | Shudihuang | *Rehmannia glutinosa* Libosch. | Earthnut | 160 g |
|  | *Corni Fructus* | Shanzhuyu | *Cornus officinalis* Sieb et Zucc. | Fruit | 80 g |
|  | *Moutan Cortex* | Mudanpi | *Paeoniasuffruticosa* Andr. | Root skin | 60 g |
|  | *Dioscoreae Rhizoma* | Shanyao | *Dioscorea opposita* Thunb | Rhizome | 80 g |
|  | *Poria* | Fuling | *Poria cocos* (Schw.) Wolf | Sclerotium | 60 g |
|  | *Alismatis Rhizoma* | Zexie | *Alisma orientale* (Sam.) Juzep. or  *Alisma plantago-aquatica* Linn. | Rhizome | 60 g |
| Chowiseungcheng decoction | *Coicis Semen* | Yiyiren | *Coix lacryma-jobi* L.var*.ma-yuen* (Roman.) Stapf. | Seed | 3.75 g |
|  | *Castaneae Semen* | Maoliren | *Castanea seguinii* Dode. | Seed | 3.75 g |
|  | *Raphani Semen* | Laifuzi | *Raphanus sativus* L. | Seed | 1.875 g |
|  | *Ephedrae Herba* | Mahuang | *Ephedra sinica* Stapf. or  *Ephedra intermedia* Schrenk et C.A.Mey. *Ephedra equisetina* Bge. | Herbaceous stem | 1.25 g |
|  | *Platycodi Radix* | Jiegeng | *Platycodon grandiflorum* (Jacq.) A.DC. | Root | 1.25 g |
|  | *Liriopis Tuber* | Maidong | *Ophiopogon japonicus* (L.f) Ker-Gawl. | Earthnut | 1.25 g |
|  | *Schizandrae Fructus* | Wuweizi | *Schisandra chinensis* (Turcz.) Baill. | Fruit | 1.25 g |
|  | *Acori Graminei Rhizoma* | Shichangpu | *Acorus tatarinowii* Schott | Rhizoma | 1.25 g |
|  | *Polygalae Radix* | Yuanzhi | *Polygala tenuifolia* Willd or  *Polygala sibirica* L. | Root | 1.25 g |
|  | *Asparagi Radix* | Tiandong | *Asparagus cochinchinensis* (Lour.) Merr. | Earthnut | 1.25 g |
|  | *Zizyphi Spinosae Semen* | Suanzaoren | *Ziziphus jujuba* Mill. var. spinosa (Bunge) Hu ex H. F. Chou | Seed | 1.25 g |
|  | *Longanae Arill* | Longyanrou | *Dimocarpus longan* Lour. | Aril | 1.25 g |
| Daesiho decoction | *Bupleuri radix* | Chaihu | *Bupleurum chinense* DC. or  *Bupleurum scorzonerifolium* Willd. | Root | 2.0 g |
|  | *Pinelliae rhizoma* | Banxia | *Pinellia ternata* (Thunb.) Breit. | Tuber | 1.33 g |
|  | *Zingiberis rhizoma* | Ganjiang | *Zingiber officinale* Rosc. | Rhizoma | 1.67 g |
|  | *Scutellariae radix* | Huangqin | *Scutellaria baicalensis* Georgi. | Root | 1.00 g |
|  | *Paeoniae radix alba* | Baishao | *Paeonia lactiflora* Pall. | Root | 1.00 g |
|  | *Jujubae fructus* | Dazao | *Ziziphus jujuba* Mill. | Fruit | 1.00 g |
|  | *Aurantii fructus immaturus* | Zhishi | *Citrus aurantium* L. or  *Citrus sinensis* Osbeck | Immature fruit | 0.67 g |
|  | *Rhei radix et rhizoma* | Dahuang | *Rheum palmatum* L. or  *Rheum tanguticum* Maxim. ex Bal£. or  *Rheum officinale* Baill. | Root/ Rhizome | 0.67 g |
| Kangshuailao tablet | *Rehmanniae radix* | Dihuang | *Rehmannia glutinosa* Libosch. | Root | Unknown |
|  | *Ginseng radix et rhizoma* | Renshen | *Panax ginseng* C. A. Mey. | Root/ Rhizome |  |
|  | *Asparagi Radix* | Tiandong | *Asparagus cochinchinensis* (Lour.) Merr. | Earthnut |  |
|  | *Liriopis Tuber* | Maidong | *Ophiopogon japonicus* (L.f) Ker-Gawl. | Earthnut |  |
|  | *Lycii fructus* | gouqizi | *Lycium chinense* Miller. | Fruit |  |
|  | *Poria* | Fuling | *Poria cocos* (Schw.) Wolf. | Sclerotium |  |
| Jianpi Tiaogan drink | *Scutellariae radix* | Huangqin | *Scutellaria baicalensis* Georgi. | Root | Unknown |
|  | *Bupleuri* *radix* | Chaihu | *Bupleurum chinense* DC. or  *Bupleurum scorzonerifolium* Willd. | Root |  |
|  | *Poria* | Fuling | *Poria cocos* (Schw.) Wolf. | Sclerotium |  |
|  | *Salviae miltiorrhizae radix et rhizoma* | Danshen | *Salvia miltiorrhiza* Bge. | Root/ Rhizome |  |
|  | *Coicis Semen* | Yiyiren | *Coix lacryma-jobi* L.var*.ma-yuen* (Roman.) Stapf. | Seed |  |
|  | *Paeoniae radix alba* | Baishao | *Paeonia lactiflora* Pall. | Root |  |
|  | *Cassiae semen* | Juemingzi | *Cassia obtusifolia* L. | Seed |  |
|  | *Pogostemonis herba* | Guanghuoxiang | *Pogostemon cablin* (Blanco) Benth. | Aerial Part |  |
|  | *Alismatis rhizoma* | Zexie | *Alisma orientale* (Sam.) Juzep. or  *Alisma plantago-aquatica* Linn. | Tuber |  |
|  | *Rhei radix et rhizoma* | Dahuang | *Rheum palmatum* L. or  *Rheum tanguticum* Maxim. ex Bal£. or  *Rheum officinale* Baill. | Root/ Rhizome |  |
| Ginseng extract Sanggua drink | *Mori folium* | Sangye | *Morus alba* L. | Leaf | 4 g |
|  | *Puerariae lobatae radix* | Gegen | *Pueraria lobata* (Willd.) Ohwi | Root | 6 g |
|  | *Dioscoreae rhizoma* | Shanyao | *Dioscorea opposita* Thunb. | Root | 3 g |
|  | *Momordicae Charantiae* | Kugua | *Momordica charantia* L. | Fruit | 3 g |
| Salvia-Nelumbinis naturalis formula | *Gynostemmae Pentaphylli herba* | Jiaogulan | *Gynostemma pentaphyllum* (Thunb.) Mak. | Aerial Part | 15 g |
|  | *Polygoni Cuspidati Rhizoma et Radix* | Huzhang | *Panax quinquefolium* L. | Root/ Rhizome | 15 g |
|  | *Salviae miltiorrhizae Radix et Rhizoma* | Danshen | *Salvia miltiorrhiza* Bge. | Radix/ Rhizome | 9 g |
|  | *Artemisiae Scopariae Herba* | Yinchen | *Artemisia scoparia* Waldst.etKit. or  *Artemisia capillaris* Thunb. | Aerial Part | 9 g |
|  | *Nelumbinis Folium* | Heye | *Nelumbo nucifera* Gaertn. | Leaf | 6 g |
| Shenling Baizhu powder | *Ginseng Radix et Rhizoma* | Renshen | *Panax ginseng* C.A.Mey. | Root/ Rhizome | 5 g |
|  | *Poria* | Fuling | *Poria cocos* (Schw.) Wolf | Sclerotium | 5 g |
|  | *Atractylodis Macrocephalae Rhizoma* | Baizhu | *Atractylodes macrocephala* Koidz. | Rhizome | 5 g |
|  | *Dioscoreae Rhizoma* | Shanyao | *Dioscorea opposita* Thunb. | Rhizome | 5 g |
|  | *Lablab Semen Album* | Baibiandou | *Dolichos lablab* L. | Seed | 4 g |
|  | *Nelumbinis Semen* | Lianzi | *Nelumbo nucifera* Gaertn. | Seed | 3 g |
|  | *Glycyrrhizae Radix et Rhizoma Praeparata Cum Melle* | Gancao | *Glycyrrhiza uralensis* Fisch. or  *Glycyrrhiza inflata* Bat. or  *Glycyrrhiza glabra* L | Root/ Rhizome | 3 g |
|  | *Coicis Semen* | Yiyiren | *Coix lacryma-jobi* L.var.ma-yuen (Roman.) Stapf | Seed | 3 g |
|  | *Platycodonis Radix* | Jiegeng | *Platycodon grandiflorum* (Jacq.) A.DC. | Root | 2 g |
|  | *Amomi Fructus* | Sharen | *Amomum villosum* Lour. or  *Amomum villosum* Lour.var.xanthioides T.L.Wu et Senjen or  *Amomum longiligulare* T.L.Wu | Fruit | 2 g |
| Qushi Huayu decoction | *Artemisiae Scopariae Herba* | Yinchen | *Artemisia scoparia* Waldst.etKit. or  *Artemisia capillaris* Thunb. | Aerial Part | 15 g |
|  | *Polygoni Cuspidati Rhizoma et Radix* | Huzhang | *Panax quinquefolium* L. | Root/ Rhizome | 12 g |
|  | *Curcumae Longae Rhizoma* | Jianghuang | *Curcuma Longa* L. | Rhizome | 9 g |
|  | *Gardeniae Fructus* | Zhizi | *Gardenia jasminoides* Ellis | Fruit | 12 g |
|  | *Hyperici japonici Herba* | Tianjihuang | *Hypericum japonicum* Thunb.ex Murray | Whole plant | 9 g |
| Dachaihu decoction | *Bupleuri Radix* | Chaihu | *Bupleurum chinense* DC. *or*  *Bupleurum scorzonerifolium* Willd. | Root | 12 g |
|  | *Scutellaria radix* | Huangqin | *Scutellaria baicalensis* Georgi | Root | 9 g |
|  | *Paeoniae Radix Alba* | Baishao | *Paeonia lactiflora* Pall. | Root | 9 g |
|  | *Pinelliae Rhizoma* | Banxia | *Pinellia ternata* (Thunb.) Makino | Rhizome | 9 g |
|  | *Citri Reticulatae Pericarpium* | Chenpi | *Citrus reticulata* Blanco | Pericarp | 9 g |
|  | *Zingiberis Rhizoma Recens* | Shengjiang | *Zingiber officinale* Rosc. | Rhizome | 15 g |
|  | *Jujubae Fructus* | Dazao | *Ziziphus jujuba* Mill. | Fruit | 12 g |
|  | *Rhei Radix et Rhizoma* | Dahuang | *Rheum palmatum* L. or  *Rheum tanguticum* Maxim. ex Bal£. or  *Rheum officinale* Baill. | Root/ Rhizome | 6 g |
| Qianggan formula | *Artemisiae Scopariae Herba* | Yinchen | *Artemisia scoparia* Waldst.etKit. or  *Artemisia capillaris* Thunb. | Aerial Part | 10 g |
|  | *Isatidis Radix* | Banlangen | *Isatis indigotica* Fort. | Root | 5 g |
|  | *Angelicae sinensis Radix* | Danggui | *Angelica sinensis* (Oliv.) Diels | Root | 5 g |
|  | *Paeoniae Radix Alba* | Baishao | *Paeonia lactiflora* Pall. | Root | 5 g |
|  | *Salviae miltiorrhizae Radix et Rhizoma* | Danshen | *Salvia miltiorrhiza* Bge. | Radix/ Rhizome | 10 g |
|  | *Curcumae Radix* | Yujin | *Curcuma wenyujin* Y. H. Chen et C. Ling or *Curcuma Longa* L. or *Curcuma kwangsiensis* S. G. Lee et C. F. Liang or *Curcuma phaeocaulis* Val. | Earthnut | 5 g |
|  | *Astragali Radix* | Huangqi | *Scutellaria baicalensis* Georgi | Root | 10 g |
|  | *Codonopsis Radix* | Dangshen | *Codonopsis pilosula* (Franch.) Nannf. or *Codonopsis pilosula* Nannf.var.modesta (Nannf.) L.T.Shen or *Codonopsis tangshen* Oliv | Root | 5 g |
|  | *Alismatis Rhizoma* | Zexie | *Alisma orientale* (Sam.) Juzep. or  *Alisma plantago-aquatica* Linn. | Rhizome | 5 g |
|  | *Polygonati Rhizoma* | Huangjing | *Polygonatum kingianum* Coll.et Hemsl. or *Polygonatum sibiricum* Red. or  *Polygonatum cyrtonema* Hua | Rhizome | 5 g |
|  | *Rehmanniae Radix* | Dihuang | *Rehmannia glutinosa* Libosch. | Earthnut | 5 g |
|  | *Dioscoreae Rhizoma* | Shanyao | *Dioscorea opposita* Thunb. | Rhizome | 5 g |
|  | *Crataegi Fructus* | Shanzha | *Crataegus pinnatifida* Bge. var. major  N. E. Br. or *Crataegus pinnatifida* Bge. | Fruit | 4 g |
|  | *Massa Medicata Fermentata* | Shenqu | *Medicated Leaven Massa Medicata Fermentata* | Zymotic product | 4 g |
|  | *Gentianae Macrophyllae Radix* | Qinjiao | *Gentiana macrophylla* Pall. or  *Gentiana straminea* Maxim. or  *Gentiana crassicaulis* Duthie ex Burk. or  *Gentiana dahurica* Fisc. | Root | 4 g |
|  | *Glycyrrhizae Radix* | Gancao | *Glycyrrhiza uralensis* Fisch. or  *Glycyrrhiza inflata* Bat. or  *Glycyrrhiza glabra* L. | Root/ Rhizome | 4 g |
| Jiangan Jiangzhi pills | *Polygoni Multiflori Radix* | Heshouwu | *Polygonum multiflorum* Thunb. | Earthnut | 20 g |
|  | *Poria* | Fuling | *Poria cocos* (Schw.) Wolf | Sclerotium | 30 g |
|  | *Curcumae Radix* | Yujin | *Curcuma wenyujin* Y. H. Chen et C. Ling or *Curcuma Longa* L. or  *Curcuma kwangsiensis* S. G. Lee et C. F. Liang or *Curcuma phaeocaulis* Val. | Earthnut | 20 g |
|  | *Cassiae Semen* | Juemingzi | *Cassia obtusifolia* L. or *Cassia tora* L. | Seed | 15 g |
|  | *Crataegi Fructus* | Shanzha | *Crataegus pinnatifida* Bge. var. major  N. E. Br. or *Crataegus pinnatifida* Bge. | Fruit | 30 g |
|  | *Salviae miltiorrhizae Radix et Rhizoma* | Danshen | *Salvia miltiorrhiza* Bge. | Radix/ Rhizome | 15 g |
|  | *Aurantii Fructus Immaturus* | Zhishi | *Citrus aurantium* L. or  *Citrus sinensis* Osbeck | Fruit | 15 g |
|  | *Curcumae Longae Rhizoma* | Jianghuang | *Curcuma Longa* L. | Rhizome | 10 g |
|  | *Alismatis Rhizoma* | Zexie | *Alisma orientale* (Sam.) Juzep. or  *Alisma plantago-aquatica* Linn. | Rhizome | 15 g |
|  | *Nelumbinis Folium* | Heye | *Nelumbo nucifera* Gaertn. | Leaf | 20 g |
| Erchen decoction | *Pinelliae Rhizoma* | Banxia | *Pinellia ternata* (Thunb.) Makino | Rhizome | 15 g |
|  | *Citri Reticulatae Pericarpium* | Chenpi | *Citrus reticulata* Blanco | Pericarp | 15 g |
|  | *Poria* | Fuling | *Poria cocos* (Schw.) Wolf | Sclerotium | 9 g |
|  | *Glycyrrhizae Radix* | Gancao | *Glycyrrhiza uralensis* Fisch. or  *Glycyrrhiza inflata* Bat. or  *Glycyrrhiza glabra* L. | Root/ Rhizome | 4.5 g |
|  | *Zingiberis Rhizoma Recens* | Shengjiang | *Zingiber officinale* Rosc. | Rhizome | 7 pieces |
|  | *Mume Fructus* | Wumei | Prunus mume (Sieb.) Sie.et Zucc. | Fruit | 1 piece |
| Jiangan Xiaozhi decoction | *Salviae miltiorrhizae Radix et Rhizoma* | Danshen | *Salvia miltiorrhiza* Bge. | Root/ Rhizome | 15 g |
|  | *Notoginseng Radix et Rhizoma* | Sanqi | *Panax notoginseng* ( Burk.) F. H. Chen | Root/ Rhizome | 6 g |
|  | *Curcumae Rhizoma* | Ezhu | *Curcuma phaeocaulis* VaL. or  *Curcuma kwangsiensis* S.G.Lee et C.F.Liang *Curcuma wenyujin* Y.H.ChenetC.Ling | Rhizome | 15 g |
|  | *Crataegi Fructus* | Shanzha | *Crataegus pinnatifida* Bge. var. major  N. E. Br. or  *Crataegus pinnatifida* Bge. | Fruit | 20 g |
|  | *Astragali Radix* | Huangqi | *Astragalus membranaceus* (Fisch.) Bge.var.mongholicus (Bge.) Hsiao or  *Astragalus membranaceus* (Fisch.) Bge. | Root | 20 g |
|  | *Vaticae Fructus* | Qingmei | *Vatica mangachapoi* Blanco | Fruits | 10 g |
|  | *Paeoniae Radix Rubra* | Chishao | *Paeonia lactiflora* Pall. or  *Paeonia veitchii* Lynch | Root | 20 g |
|  | *Curcumae Longae Rhizoma* | Jianghuang | *Curcuma Longa* L. | Rhizome | 12 g |
|  | *Alismatis Rhizoma* | Zexie | *Alisma orientale* (Sam.) Juzep. or  *Alisma plantago-aquatica* Linn. | Rhizome | 15 g |
|  | *Chrysanthemi Flos* | Juhua | *Chrysanthemum morifolium* Ramat. | Flower | 15 g |
|  | *Nelumbinis Folium* | Heye | *Nelumbo nucifera* Gaertn. | Leaf | 15 g |
|  | *Glycyrrhizae Radix* | Gancao | *Glycyrrhiza uralensis* Fisch. or  *Glycyrrhiza inflata* Bat. or  *Glycyrrhiza glabra* L. | Root/ Rhizome | 6 g |
| Jiangzhi granules | *Gynostemmae Pentaphylli herba* | Jiaogulan | *Gynostemma pentaphyllum* (Thunb.) Mak. | Whole plant | 15 g |
|  | *Polygoni Cuspidati Rhizoma et Radix* | Huzhang | *Panax quinquefolium* L. | Root/ Rhizome | 15 g |
|  | *Nelumbinis Folium* | Heye | *Nelumbo nucifera* Gaertn. | Leaf | 6 g |
|  | *Artemisiae Scopariae Herba* | Yinchen | *Artemisia scoparia* Waldst.etKit. or  *Artemisia capillaris* Thunb. | Aerial Part | 9 g |
|  | *Salviae miltiorrhizae Radix et Rhizoma* | Danshen | *Salvia miltiorrhiza* Bge. | Root/ Rhizome | 9 g |
| Chaihu Shugan powder | *Bupleuri Radix* | Chaihu | *Bupleurum chinense* DC. *or*  *Bupleurum scorzonerifolium* Willd. | Root | 6 g |
|  | *Chuanxiong Rhizoma* | Chuanxiong | *Ligusticum chuanxiong* Hort. | Rhizome | 5 g |
|  | *Aurantii Fructus* | Zhiqiao | *Citrus aurantium* L. | Fruit | 5 g |
|  | *Citri Reticulatae Pericarpium* | Chenpi | *Citrus reticulata* Blanco | Pericarp | 6 g |
|  | *Paeoniae Radix Alba* | Baishao | *Paeonia lactiflora* Pall. | Root | 5 g |
|  | Cyperi Rhizoma | Xiangfu | Cyperus rotundus L. | Rhizome | 5 g |
|  | *Glycyrrhizae Radix et Rhizoma Praeparata Cum Melle* | Gancao | *Glycyrrhiza uralensis* Fisch. or  *Glycyrrhiza inflata* Bat. or  *Glycyrrhiza glabra* L. | Root/ Rhizome | 3 g |
| Shugan Xiaozhi decoction | *Artemisiae Scopariae Herba* | Yinchen | *Artemisia scoparia* Waldst.etKit. or  *Artemisia capillaris* Thunb. | Aerial Part | 6 g |
|  | *Nelumbinis Folium* | Heye | *Nelumbo nucifera* Gaertn. | Leaf | 6 g |
|  | *Pumice Stone* | Fushi | *Pumex* | Cellular stone | 6 g |
|  | Cassiae Semen | Juemingzi | *Cassia obtusifolia* L. or *Cassia tora* L. | Seed | 6 g |
|  | *Crataegi Fructus* | Shanzha | *Crataegus pinnatifida* Bge. var. major  N. E. Br. or *Crataegus pinnatifida* Bge. | Fruit | 6 g |
|  | *Alismatis Rhizoma* | Zexie | *Alisma orientale* (Sam.) Juzep. or  *Alisma plantago-aquatica* Linn. | Rhizome | 6 g |
|  | *Poria* | Fuling | *Poria cocos* (Schw.) Wolf | Sclerotium | 4 g |
|  | *Aurantii Fructus* | Zhiqiao | *Citrus aurantium* L. | Fruit | 3 g |
|  | *Bupleuri Radix* | Chaihu | *Bupleurum chinense* DC. *or*  *Bupleurum scorzonerifolium* Willd. | Root | 2 g |
|  | *Gardeniae Fructus* | Zhizi | *Gardenia jasminoides* Ellis | Fruit | 2 g |
|  | *Paeoniae Radix Alba* | Baishao | *Paeonia lactiflora* Pall. | Root | 1 g |
|  | *Glycyrrhizae Radix* | Gancao | *Glycyrrhiza uralensis* Fisch. or  *Glycyrrhiza inflata* Bat. or  *Glycyrrhiza glabra* L. | Root/ Rhizome | 1 g |
| Hongqi Jiangzhi formula | *Astragali Radix* | Huangqi | *Astragalus membranaceus*(Fisch.) Bge.var.mongholicus (Bge.) Hsiao or  *Astragalus membranaceus* (Fisch.) Bge. | Root | 15 g |
|  | Rice fermented with the mould Monascus purpureus (Red Rice) | Hongqu | *Monascus purpureus* Went | Zymotic product | 12 g |
|  | *Artemisiae Scopariae Herba* | Yinchen | *Artemisia scoparia* Waldst.etKit. or  *Artemisia capillaris* Thunb. | Aerial Part | 10 g |
|  | *Lycii Fructus* | Gouqizi | *Lycium barbarum* L. | Fruit | 10 g |
|  | *Curcumae Longae Rhizoma* | Jianghuang | *Curcuma Longa* L. | Rhizome | 6 g |
|  | *Nelumbinis Folium* | Heye | *Nelumbo nucifera* Gaertn. | Leaf | 10 g |
|  | *Magnoliae Officinalis Cortex* | Houpo | *Magnolia officinalis* Rehd.et Wils. M*agnolia officinalis* Rehd.et Wils.var.biloba Rehd.et Wils. | Cortex | 6 g |
| Simiao decoction | *Atractylodis rhizoma* | Cangzhu | *Atractylodes lancea* (Thunb.) DC. | Rhizome | 12 g |
|  | *Phellodendri amurensis cortex* | Huangbo | *Phellodendron amurense* Rupr. | Cortex | 12 g |
|  | *Achyranthis bidentatae radix* | Niuxi | *Achyranthes bidentata* Bl. | Root | 12 g |
|  | *Coicis Semen* | Yiyiren | *Coix lacryma-jobi* L.var*.ma-yuen* (Roman.) Stapf. | Seed | 30 g |
| Quzhuo Tongbi decoction | *Smilacis glabrae rhizoma* | Tufuling | *Smilax glabra* Roxb. | Rhizome | 60 g |
|  | *Dioscoreae spongiosae rhizoma* | Bixie | *Dioscorea spongiosa* J. Q. Xi，M. Mizuno et W. L. Zhao | Rhizome | 30 g |
|  | *Corn Stigma* | Yumixu | *Zea mays* L. | Stigma | 15 g |
|  | *Coicis Semen* | Yiyiren | *Coix lacryma-jobi* L. var. ma-yuen (Rom.Caill.) Stapf | Seed | 30 g |
|  | *Siegesbeckiae Herba* | Xixiancao | *Siegesbeckia orientalis* L. | Whole plant | 18 g |
|  | *Curcumae Longae Rhizoma* | Jianghuang | *Curcuma longa* L. | Rhizome | 12 g |
|  | *Taxilli Herba* | Sangjishen | *Taxillus chinensis* (DC.) Danser. | Branch/Leaf | 15 g |
|  | *Corydalis Rhizoma* | Yanhusuo | *Corydalis yanhusuo* (Y. H. Chou and Chun C. Hsu) W. T. Wang ex Z. Y. Su and C. Y. Wu | Tuber | 18 g |
|  | *Citri Sarcodactylis Fructus* | Foshou | *Citrus medica* L. var. sarcodactylis Swingle. | Fruit | 12 g |
| Shanmei capsule | *Crataegi folium* | Shanzhaye | *Crataegus pinnatifida* Bge. var. major  N. E. Br. or *Crataegus pinnatifida* Bge. | Leaf | Unknown |
|  | *Davuricae fructus rosae* | Cimeiguo | *Rosa davurica* Pall. | Fruit |  |
| Shener Jiangzhi formula | *Acanthopanacis senticosi radix et rhizoma seu caulis* | Ciwujia | *Acanthopanax senticosus (Rupr.etMaxim.) Harms* | Root/ Rhizome | 265 g |
|  | *Lonice raejaponicae caulis* | Rendongteng | *Lonicera japonica* Thunb. | Stem | 177 g |
|  | *Crataegi fructus* | Shanzha | *Crataegus pinnatifida* Bge. | Fruit | 177 g |
|  | *Auricularia auricula* | Heimuer | *Auricularia auricular* (L.) Underw. | Fruiting body | 133 g |
| Jieyu Qutan Huazhuo formula | *Cyperi rhizoma* | Xiangfu | *Cyperus rotundus* L. | Rhizome | 10 g |
|  | *Citri reticulatae pericarpium* | Chenpi | *Citrus reticulata* Blanco | Pericarp | 8 g |
|  | *Pinelliae rhizoma* | Fabanxia | *Pinellia ternata* (Thunb.) Breit. | Stem tuber | 9 g |
|  | *Poria* | Fuling | *Poria cocos* (Schw.) Wolf | Sclerotium | 10 g |
|  | *Atractylodis rhizoma* | Cangzhu | *Atractylodes lancea* (Thunb.) DC. | Rhizome | 10 g |
|  | *Amomi fructus* | Sharen | *Amomum villosum* Lour. or  *Amomum villosum* Lour.var.xanthioides T.L.Wu et Senjen or  *Amomum longiligulare* T.L.Wu | Fruit | 5 g |
|  | *Massa Medicata Fermentata* | Shenqu | Medicated Leaven Massa Medicata Fermentata | Zymotic product | 15 g |
|  | *Crataegi fructus* | Shanzha | *Crataegus pinnatifida* Bge. var. major  N. E. Br. *or Crataegus pinnatifida* Bge. | Fruit | 15 g |
|  | *Gardeniae fructus* | Zhizi | *Gardenia jasminoides* Ellis. | Fruit | 10 g |
|  | *Chuanxiong rhizoma* | Chuanxiong | *Ligusticum chuanxiong* Hort. | Rhizome | 9 g |
| Tianhuang formula | *Notoginseng radix et rhizoma* | Sanqi | *Panax notoginseng* (Burk.) F. H. Chen | Root/ Rhizome | 600 g |
|  | *Coptidis rhizoma* | Huanglian | *Coptis chinensis* Franch. or  *Coptis deltoidea* C.Y.Cheng et Hsiao or  *Coptis teeta* Wall. | Rhizome | 500 g |
| Wuwei Qingzhuo powder | *Granati pericarpium* | Shiliu | *Punica granarum* L. | Pericarp | 400 g |
|  | *Carthami flos* | Honghua | *Carthamus tinctorius* L. | Flower | 200 g |
|  | *Amomi fructus rotundus* | Doukou | *Amomum kravanh* Pierre ex Gagnep. *or*  *Amomum compactum* Soland ex Maton | Fruit | 50 g |
|  | Cinnamomi cortex | Rougui | *Cinnamomum cassia* Presl | Cortex | 50 g |
|  | *Piperis longi fructus* | Biba | *Piper longum* L. | Bunch | 50 g |
| Yinian Kangbao tea | *Nelumbinis folium* | Heye | *Nelumbo nucifera* Gaertn. | Leaf | Unknown |
|  | *Crataegi Fructus* | Shanzha | *Crataegus pinnatifida* Bge. | Fruit |  |
|  | *Lycii Fructus* | Gouqizi | *Lycium barbarum* L. | Fruit |  |
|  | *Mori Folium* | Sangye | *Morus alba* L. | Leaf |  |
|  | *Dioscoreae Rhizoma* | Shanyao | *Dioscorea opposita* Thunb. | Rhizome |  |
|  | *Lily Bulbus* | Baihe | *Lilium lancifolium* Thunb. | Meaty scale leaves |  |
|  | *Poria* | Fuling | *Poria cocos (Schw.)* Wolf | Sclerotium |  |
|  | *Glycyrrhizae Radix ET Rhizoma* | Gancao | *Glycyrrhiza uralensis* Fisch. | Root/ Rhizome |  |
|  | *Alpiniae Officinarum Rhizoma* | Gaoliangjiang | *Alpinia officinarum* Hance. | Rhizome |  |
|  | *Puerariae Lobatae Radix* | Gegen | *Pueraria lobata (Willd.)* Ohwi | Root |  |
|  | *Platycodon Radix* | Jiegeng | *Platycodon grandiflorum* (jacp.) A. DC. | Root |  |
|  | *Nelumbinis Semen* | Lianzi | *Nelumbo nucifera* Gaertn. | Seed |  |
|  | *Portulacae Herba* | Machixian | *Portulaca oleracea* L. | Whole plant |  |
|  | *Cinnamomi Cortex* | Rougui | *Cinnamomum cassia* Presl | Cortex |  |
|  | *Polygonati Odorati Rhizoma* | Yuzhu | *Polygonatum odoratum* (MilL.) Druce | Rhizome |  |
| Congxin Lunzhi formula | *Coptidis Rhizoma* | Huanglian | *Coptis chinensis* Franch. or  *Coptis deltoidea* C.Y.Cheng et Hsiao or  *Coptis teeta* Wall. | Rhizome | 6 g |
|  | *Ganoderam* | Lingzhi | *Ganoderma lucidum* (Leyss.ex Fr.) Karst. *Ganoderma sinense* Zhao，Xu et Zhang | Sporophore | 10 g |
|  | *Ziziphi Spinosae Semen* | Suanzaoren | *Ziziphus jujuba Mill. var. spinosa* (Bunge) Hu ex H. F. Chou | Seed | 20 g |
|  | *Salviae miltiorrhizae Radix et Rhizoma* | Danshen | *Salvia miltiorrhiza* Bge. | Root/ Rhizome | 15 g |
| Qijian mixture | *Astragali Radix* | Huangqi | *Astragalus membranaceus* (Fisch.) or  Bge.var.mongholicus (Bge.) Hsiao or  *Astragalus membranaceus* (Fisch.) Bge. | Root | Unknown |
|  | [*Winged Euonymus Twig*](javascript:;) | Guijianyu | *Euonymus alatus* (Thunb.) Sieb. | Twig |  |
|  | *Salviae miltiorrhizae Radix et Rhizoma* | Danshen | *Salvia miltiorrhiza* Bge. | Root/ Rhizome |  |
|  | *Dioscoreae Rhizoma* | Shanyao | *Dioscorea oppositifolia* L. | Root/ Rhizome |  |
|  | *Rehmanniae Radix* | Dihuang | *Rehmannia glutinosa* Libosch. | Earthnut |  |
|  | *Puerariae Lobatae Radix* | Gegen | *Pueraria lobata* (Willd.) Ohwi | Root |  |
|  | *Coptidis Rhizoma* | Huanglian | *Coptis chinensis* Franch. or  *Coptis deltoidea* C.Y.Cheng et Hsiao or *Coptis teeta* Wall. | Rhizome |  |
|  | *Trichosanthis Radix* | Tianhuafen | *Trichosanthes kirilowii* Maxim. or  *Trichosanthes rosthornii* Harms | Root |  |
|  | *Poria* | Fuling | *Poria cocos* (Schw.) Wolf | Sclerotium |  |
|  | *Liriopis Tuber* | Maidong | *Ophiopogon japonicus* (L.f) Ker-Gawl. | Earthnut |  |

**Supplementary Table S2** Effect of herbal medicine on gut microbiota and its potential mechanism in the treatment of T2DM.

| Herbal medicine | Subject | Outcome | Potential mechanism | Changes in gut microbiota | Reference |
| --- | --- | --- | --- | --- | --- |
| Naoxintong capsule | SD rats | TG ↓, TC ↓, LDL-C ↓, FFA ↓ | 1. Inflammation: IL-1β ↓, IL-6 ↓, TNF-α ↓, CRP ↓  2. AAs: alanine ↑, tyrosine ↑, tryptophan ↑  3. BAs: GCA ↓, GCDCA ↓ | 1. Family level: Ruminococcaceae ↑, Helicobacteraceae ↓  2. Genus level: *Ruminococcus* 1 ↑ | (Yan et al., 2020) |
| Xiexin decoction | SD rats | TC ↓, TG ↓, LDL-C ↓, FFA ↓, HOMA-IR ↓, HDL-C ↑, HOMA-ISI ↑ | 1. Inflammation: CRP ↓, IL-6 ↓, IL-1β ↓, TNF-α ↓  2. SCFAs: acetic acid ↑, propionic acid ↑, isobutyric acid ↑, butyric acid ↑ | 1. Phylum level: Actinobacteria ↑  2. SCFA-producing bacteria: *Alloprevotella* ↑, *Barnesiella* ↑, *Eubacterium Ventriosum* group ↑, *Lachnospiraceae* UCG 001 ↑, *Papillibacter* ↑, *Prevotellaceae* NK3B31 group ↑ | (Wei et al., 2018) |
| Huanglian Jiedu decoction | SD rats | FBG ↓, TC ↓, TG ↓, LDL-C↓, HOMA-IR ↓, FINS ↓ | 1. Inflammation: CRP ↓, IL-6 ↓, IL-1β ↓, TNF-α ↓  2. AAs: valine ↓, leucine ↓, isoleucine ↓ | 1. Phylum level: Bacteroidetes ↑, F/B ↓  2. Genus level: *Akkermansia* ↑  3. SCFA-producing bacteria: *Adlercreutzia* ↑, *Porphyromonadaceae* ↑, *Lachnospiraceae* ↑ | (Chen et al., 2018) |
| *Scutellaria baicalensis* Georgi (Labiatae; *Scutellariae radix*) and *Coptis chinensis Franch.* (Ranunculaceae; *Coptidis rhizoma*) | SD rats | FBG ↓, TG ↓, TC ↓, LDL-C ↓, FFA ↓, FINS ↓, HDL-C ↑ | 1. Inflammation: CRP ↓, IL-6 ↓, IL-1β ↓, TNF-α ↓  2. BAs: DCA ↓, CA ↓, TUDCA ↑, GCA ↑, TCA ↑ | 1. Phylum level: F/B ↓  2. SCFA-producing bacteria: *Bacteroidales* S24-7 group norank ↑, *Parasutterella* ↑, *Prevotellaceae* UCG 001 ↑  3. Secondary BA-producing bacteria: *Escherichia*-*Shigella* ↓ | (Xiao et al., 2020) |
| Jinqi Jiangtang tablet | C57BL/6J mice | FBG ↓, HbA1c ↓ | 1. Inflammation: TNF-α ↓, IL-6 ↓, MCP-1 ↓  2. SCAFs: acetic acid ↑, propionic acid ↑, butyric acid ↑ | 1. Phylum level: Verrucomicrobia ↑  2. Family level: Helicobacteraceae ↓  3. Genus level: *Desulfovibrio* ↓  4. SCFA-producing bacteria: *Akkermansia* ↑ | (Cao et al., 2019) |
| Gegen Jiaotai pills | SD rats | FBG ↓, TG ↓, TC ↓, LDL-C ↓, HOMA-IR ↓, HDL-C ↑ | 1. BA receptors: FXR ↑, TGR5 ↑  2. Colon BAs: CA ↑, TCA ↑, GCA ↑, CDCA ↑, TCDCA ↑, GCDCA ↑ | 1. Phylum level: Proteobacteria ↓  2. Genus level: *Lactobacillus* ↑ | (Chen et al., 2021a) |
| *Edgeworthia gardneri* (Wall.) Meisn. (Thymelaeaceae; *Edgeworthiae rhei radix et flos*) and *Carthamus tinctorius* L. (Compositae; *Carthami flos*) | ZLN and ZDF rats | FBG ↓, TC ↓, TG ↓, FFA ↓, HOMA-IR ↓, FINS ↓ | 1. Gut barrier: occludin ↑  2. Inflammation: TNF-α ↓, IL-6 ↓, LPS ↓, MyD88 ↓, CTSK ↓, TLR4 ↓ | Phylum level: Actinobacteria ↑, F/B ↓ | (Li et al., 2021b) |
| Shenqi compound | GK rats | FBG ↓, INS ↑ | 1. Gut barrier: ZO-1 ↑  2. Inflammation: LPS ↓  3. AAs: isoleucine ↑, tyrosine ↑, alanine ↑  4. SCFA: butyric acid ↑ | 1. Phylum level: F/B *↓*  2. Family level: Prevotellaceae ↑  3. SCFA-producing bacteria: *Butyricimonas* ↑, *Bacteroides* ↑, *Blautia* ↑, *Roseburia* ↑ | (Zhang et al., 2022d) |
| Gegen Qinlian decoction | GK rats | FBG ↓, HOMA-IR ↓ | 1. SCFAs: acetic acid ↑, butyric acid ↑  2. Inflammation: IL-1β ↓, IL-6 ↓, IL-17 ↓, TNF-α ↓, IFN-γ ↓, MCP-1 ↓  3. Immune-related genes: NF-κB *↓* | 1. Genus level: *Alistipes* ↑, *Butyricimonas* ↑  2. SCFA-producing bacteria: *Faecalibacterium* ↑, *Roseburia* ↑, *Dorea* ↑, *Clostridium* XIVa ↑, *Ruminococcus* 2 ↑, *Butyricicoccus* ↑ | (Xu et al., 2020) |
| *Lycium barbarum* L. (Solanaceae; *Lycii* leaves) | Rats | FBG ↓, TG ↓, LDL-C ↓, FFA ↓, ALT ↓, AST ↓, INS ↑ | Histidinal ↑, L-allothreonine ↑ | Genus level: *Ruminococcus* 1 ↓ | (Zhao et al., 2020) |
| Jiangtangjing granules | SD rats | FBG ↓, HbA1c ↓, INS ↑ | 1. GLP-1 ↑  2. Signaling pathway: cAMP/PKA ↑ | Phylum level: Bacteroidetes ↑ | (Sun et al., 2022b) |
| *Scutellaria baicalensis* Georgi (Labiatae; *Scutellariae radix*) | SD rats | BW ↓, FBG ↓, TC ↓, TG ↓, LDL-C ↓, HDL-C ↓, INS ↓, HOMA-IR ↓ | 1. BA receptors: CYP7A1 ↑, FXR ↓  2. BAs: DCA ↓, LCA ↓, GDCA ↓, GLCA ↓, GUDCA ↓, TLCA ↓ | Genus level: *Lactobacillus* ↑, *Faecalibaculum* ↑ | (Zhao et al., 2021a) |
| *Ganoderma lucidum* (Leyss.ex Fr.) Karst. (Polyporaceae; *Ganoderma*) | SD rats | TC ↓, TG ↓, HDL-C ↑, INS1 ↑, INS2 ↑ | 1. Glycogen synthesis: GYG1 ↑, G6PC ↓  2. Lipid metabolism: ACC ↓, Fads1 ↓ | 1. Phylum level: Proteobacteria ↓  2. Genus level: *Lactobacillus* ↑ | (Jiang et al., 2021b) |
| Linggui Zhugan formula | C57BL/6J mice | FBG ↓, HbA1c ↓, HOMA-IR ↓, TC ↓, TG ↓, LDL-C ↓, FFA ↓ | GLP-1 ↑, PYY ↑ | 1. Phylum level: Firmicutes ↓, Bacteroidetes ↑  2. Genus level: *Lactobacillus* ↑, *Bacteroides* ↑ | (Wu et al., 2019) |
| Shenlian decoction | C57BL/KsJ-db/db mice | BW ↓, FBG ↓ | 1. Lipopolysaccharide biosynthesis ↓  2. Insulin generation ↑ | 1. Phylum level: Epsilonbacteraeota ↓, Verrucomicrobia ↑  2. Family level: Rikenellaceae ↓, Helicobacteraceae ↓  3. Species level: *Bacteroides caecimuris* ↑, *Bacteroides acidifaciens* ↑ | (Sun et al., 2022a) |
| Liuwi Dihuang pills | GK rats | FBG ↓, INS ↓ | 1. SCFAs: acetic acid ↑, propionic acid ↑, butyric acid ↑  2. Signaling pathway: GPR43/41 ↑, GLP-1 ↑ | 1. Phylum level: Proteobacteria ↓  2. Genus level: *Faexalibacterium* ↓  3. SCFA-producing bacteria: *Lactobacillus* ↑ | (Yi et al., 2022) |
| *Berberis kansuensis* C.K.Schneid. (Berberidaceae; *Berberis cortex*) | Wistar rats | BW ↓, FBG ↓, GSP ↓, HOMA-IR ↓, ISI ↑ | Inflammation: TNF-α ↓, IL-1β ↓, IL-6 ↓, LPS ↓ | 1. Phylum level: Bacteroidetes ↑, Proteobacteria ↓, F/B ↓  2. Genus level: *Akkermansia* ↑, *Ruminococcus* g*auvreauii* Group ↓, *Escherichia*-*Shigella* ↓, *Enterococcus* ↓ | (Xu et al., 2021) |
| *Plantago asiatica* L. (Plantaginaceae; *Plantaginis semen*) | Wistar rats | FBG ↓, GSP ↓, NEFA ↓, TC ↓, TG ↓, HDL-C ↓, MDA ↓, SOD ↑, T-AOC ↑ | SCAFs: acetic acid ↑, propionic acid ↑, butyric acid ↑ | Species level: *Bacteroides* *ovatus* ↑, *Lactobacillus* *fermentum* ↑, *Prevotella* *loescheii* ↑ | (Nie et al., 2019) |
| *Rheum palmatum* L. (Polygonaceae; *Rhei radix et rhizoma*) | SD rats | FBG ↓, GSP ↓, HOMA-IR ↓ | 1. Gut barrier: ZO-1 ↑, occludin ↑  2. Inflammation: LPS ↓  3. Signaling pathway: GLP-1 ↑ | 1. Phylum level: Firmicutes ↓, Bacteroidetes ↑  2. Genus level: *Lactobacillus* ↑, *Roseburia* ↑, *Akkermansia* ↑, *Desulfovibrio* ↓ | (Cui et al., 2019) |
| *Sophora flavescens* Ait. (Leguminosae; *Sophorae flavescentis radix*) | SD rats | FBG ↓, GSP ↓, TC ↓, TG ↓, LDL-C ↓, HDL-C ↑ | 1. AAs: indole ↑, tyramine ↑  2. BAs: CA ↓, DCA ↓, GCA ↓ | 1. Genus level: *Blautia* ↑, *Phascolarctobacterium* ↓, *Prevotella* ↓, *Faecalibacterium* ↓  2. BA-producing bacteria: *Ruminococcus* ↑ | (Shao et al., 2020) |
| *Ophiopogon japonicus* (L.f) Ker-Gawl. (Liliaceae; *Ophiopogonis radix*) | C57BL/6J mice | FBG ↓, HOMA-IR ↓ | SCFAs: isobutyrate ↑, acetate ↑, caprate ↑, butyrate ↑, propionate ↑, lactate ↑, valerate ↑ | 1. Phylum level: Proteobacteria ↓, Actinobacteria ↑  2. Family level: Desulfovibrionaceae ↓  3. Genus level: *Bifidobacterium* ↑ | (Wang et al., 2019a) |
| *Cyclocarya paliurus* (Batalin) Iljinsk. (Juglandaceae; *Cylocaryae paliuri folium*) | Wistar rats | FBG ↓, HOMA-IR ↓ | 1. Inflammation: IL-1β ↓, IL-6 ↓, TNF-α ↓  2. Leptin ↓, diponectin ↑, GLP-1 ↑  3. SCFAs: acetic ↑, propionic ↑, butyric acids ↑ | 1. Phylum level: F/B ↓  2. Genus level: *Lachnospiraceae* NK4A136 group ↑, *Roseburia* ↑, *Prevotellaceae* UCG 001 ↓ | (Li et al., 2021d) |
| *Sargassum fusiforme* (Harv.) Setch. (Sargassaceae; *Sargassum*) | ICR mice | FBG ↓, MDA ↓, IPGTT ↓, TC ↓, TG ↓, LDL-C ↓, HDL-C ↑, CAT ↑, SOD ↑ | AAs: L-valine ↓, L-isoleucine ↓, aromatic amino acids ↓, L-tyrosine ↑, L-phenylalanine ↑ | Genus level: *Romboutsia* ↓, *Roseburia* ↑, *Anaerotruncus* ↑, *Lachnoclostridium* ↑, *Bifidobacterium* ↑, *Ruminococcus* ↑ | (Wu et al., 2021b) |
| Qijian mixture | Male KKay  mice | FBG ↓, TC ↓, INS ↑ | - | 1. Phylum level: Bacteroidetes ↑  2. Family level: Lachnospiraceae ↑  3. Genus level: *Lachnospiraceae* NK4A136, *Alistipes*, *Parabacteroides* ↑ | (Gao et al., 2018) |

**Abbreviations:** TG, triglyceride; TC, total cholesterol; LDL-C, low-density lipoprotein cholesterol; FFA, free fatty acid; HOMA-IR, homeostasis assessment of insulin resistance; HDL-C, high-density lipoprotein cholesterol; HOMA-ISI, homeostasis assessment of insulin sensitivity index; FBG, fasting blood glucose; FINS, fasting insulin; HbAlc, glycosylated hemoglobin; INS, insulin; ALT, alanine aminotransferase; AST, aspartate aminotransferase; BW, body weight; GSP, glycated serum protein; ISI, insulin sensitivity index; NEFA, non esterified fatty acid; MDA, malondialdehyde; SOD, superoxide dismutase; T-AOC, total antioxidant capacity; IPGTT, intraperitoneal glucose tolerance test; CAT, catalase; IL, interleukin; TNF-α, tumor necrosis factor α; CRP, C reactive protein; AA, amino acid; BA, bile acid; SCFA, short-chain fatty acid; DCA, deoxycholic acid; CA, cholic acid; TUDCA, tauroursodeoxycholic acid; GCA, glycocholic acid; TCA, taurocholic acid; FXR, farnesoid X receptor; TGR5, takeda G protein-coupled receptor 5; CDCA, chenodeoxycholic acid; TCDCA, taurochenodeoxycholic acid; GCDCA, glycoursodeoxycholic acid; LPS, lipopolysaccharide; MyD88, myeloid differentiation primary response gene 88; CTSK, cathepsin K; TLR, toll-like receptor; ZO-1, zonulaoccludens 1; IFN, interferon; MCP-1, monocyte chemoattractant protein 1; NF-κB, nuclear factor kappa B; GLP-1, glucagon-like peptide-1; cAMP, cyclic adenosine monophosphate; PKA, protein kinase A; CYP7A1, cholesterol 7α-hydroxylase; LCA, lithocholic acid; GDCA, glycodeoxycholic acid; GLCA, glycolithocholate acid; GUDCA, glycoursodeoxycholic acid; TLCA, Taurolithocholic acid; GYG1, glycogenin‐1; G6PC, glucose‐6‐phosphatase‐α; ACC, acetyl-CoA carboxylse; Fads, fatty acyl desaturases; PYY, peptide YY; GPR, G protein-coupled receptor.

**Supplementary Table S3 Effect of herbal medicine on gut microbiota and its potential mechanism in the treatment of obesity.**

| Herbal medicine | Subject | Outcome | Potential mechanism | Changes in gut microbiota | Reference |
| --- | --- | --- | --- | --- | --- |
| Chowiseungcheng decoction | C57BL/6J mice | BW ↓, LW ↓, ATW ↓, FBG ↓, TG ↓, AST ↓, ALT ↑, HDL-C ↑ | 1. Neuropeptides: AgRP ↓, NPY ↓  2. Adipokines: RBP4 ↓ | Phylum level: Firmicutes *↓*, F/B *↓* | (Ansari et al., 2016) |
| *Bupleurum chinense* DC. (Apiaceae; *Bupleuri* *radix*) | C57BL/6J mice | TG ↓, NEFA ↓, LDL-C ↓ | 1. Gene expression: ATG ↑, FASN ↓, SREBP1c ↓  2. Signaling pathway: FGF21 ↑, KLB ↑, PGC1α↑, GLUT1 ↑ | 1. Phylum level: F/B ↓, Proteobacteria ↓  2. Family level: Desulfovibrionacea ↓, Porphyromonadaceae ↑  3. Genus level: *Parabacteroides* ↑, *Lactobacillus* ↑ | (Wu et al., 2021a) |
| Daesiho decoction | C57BL/6J mice | BW ↓, LW ↓, TC ↓, TG ↓, FBG ↓, HDL-C ↑ | Lipid metabolism: ACAA2 ↑, CXCL16 ↑, SNX17 ↑, SREBF2 ↑, Vldlr ↑ | Genus level: *Roseburia* ↑, *Bacteroides* ↑, *Ruminococcus* ↑, *Bifidobacterium* ↑ | (Hussain et al., 2016) |
| Erchen decoction | C57BL/6J mice | BW ↓, BWG ↓, FBG ↓, HOMA-IR ↓, LDL-C ↓, TG ↓, ALT ↑ | SCFA: isobutyric ↑ | 1. Phylum level: Fimicutes ↓  2. Genus level: *Prevotella* ↓, *Holdemania* ↓, *Akkermansia* ↑ | (Zhao et al., 2021b) |
| Kangshuailao tablet | C57BL/6J mice | BW ↓, FBG ↓ | —— | 1. Phylum level: Firmicutes ↓, Proteobacteria ↓, F/B ↓, Bacteroidetes ↑  2. Genus level: *Bacteroides* ↑, *Alistipes* ↑, *Christensenellaceae* R-7 group ↓ | (Gong et al., 2020) |
| *Juglans mandshurica* Maxim. (Juglandaceae; *Juglandis mandshurica pericarpium*) | SD rats | TC ↓, TG ↓, LDL-C ↓, ALT ↓, AST ↓, MDA ↓, FPG ↓, INS ↓, HOMA-IR ↓, HDL-C ↑ | —— | 1. Phylum level: Firmicutes ↓, Bacteroidetes ↑  2. Genus level: *Romboutsia* ↓, *Bacteroides* ↑ | (Wang et al., 2019b) |
| *Spatholobus suberectus* Dunn (Leguminosae; *Spatholobi caulis*) | C57BL/6J mice | BW ↓, TG ↓, LDL-C ↓, HDL-C ↑ | 1. BAT: UCP1 ↑, PRDM16 ↑, PGC1α ↑, MCAD ↑, TFAM ↑  2. Inflammation: TNF-α ↓, IL-6 ↓, IL-1β ↓  3. Lipid metabolism: SREBP1 ↓, ACC ↓, FASN ↓, PPARγ ↓, PPARα ↑, HSL ↑, ATGL ↑, FAO ↑, SIRT1 ↑, CPT-1α ↑, CPT-1β ↑ | 1. Phylum level: Bacteroidetes ↑, Anaerotruncus ↑,  2. Family level: Ruminococcaceae ↑  3. Genus level: *Enterococcus* ↓, *Bacteroides* ↓, *Lactobacillus* ↑, *Candidatus*-*Arthromitus* ↑, *Ruminococcus* ↑, *Prevotella* ↑, *Adlercreutzia* ↑, *Anaerotruncus* ↑, *Parabacteroides* ↑, *Bifidobacterium* ↑ | (Zhang et al., 2019) |
| *Corydalis bungeana* Turcz. (Papaveraceae; *Corydalis bungeanae herba*) | Wistar rats | BW ↓, CCK ↓, TG ↓, TC ↓ | 1. AAs: citrulline ↑, L-lysine ↑, dimethylglycine ↓  2. BA: CA ↓ | 1. Phylum level: Proteobacteria ↓  2. Family level: Lachnospiraceae ↑, Sutterellaceae ↑, Porphyromonadaceae ↑ | (Fu et al., 2022) |
| Jianpi Tiaogan drink | C57BL/6J mice | TC ↓, TG ↓, LDL-C ↓, HDL-C ↑ | —— | 1. Phylum level: Proteobacteria ↓, Firmicutes ↓, F/B ↓  2. Family level: Erysipelotrichaceae ↓, Parvibacter ↓  3. Genus level: *Clostridium sensu stricto* 1 ↓, *Erysipelotrichia* ↓, *Erysipelotrichales* ↓ | (Dong et al., 2022) |
| *Momordica charantia* L. (Cucurbitaceae; *Momordicae charantiae fructus*) | SD rats | BW ↓, FBG ↓, HOMA-IR ↓, FBG ↓, FINS ↓, TC ↓, HDL-C ↑ | 1. Inflammation: TNF-α ↓, IL-6 ↓, MCP-1 ↓  2. Signaling pathway: NF-кB ↓ | 1. Family level: Enterobacteriaceae ↓, Desulfovibrionaceae ↓  2. Genus level: *Allobaculum* ↑, *Butyricimonas* ↑, *Faecalibacterium* ↑, *Escherichia* ↓ | (Bai et al., 2016) |
| *Ganoderma lucidum* (Leyss.ex Fr.) Karst. (Polyporaceae; *Ganoderma*) | C57BL/6J mice | BW ↓, BWG ↓, FBG ↓, FFA ↓, FINS ↓, IR ↓ | 1. Inflammation: TNF-α↓, IL-1β ↓, IL-6 ↓, PAI-1 ↓, IL-10 ↑, TLR4 ↓  2. Gut barrier: ZO-1 ↑, occludin ↑ | 1. Phylum level: F/B ↓, Proteobacteria ↓  2. Species level: *Bacteroides* spp. ↑, *Anaerotruncus colihominis* ↑, *Roseburia hominis* ↑, *Clostridium* cluster ↑, *Escherichia fergusonii* ↓, *Oscillibacter valericigenes* ↓, *Lactococcus lactis* ↓ | (Chang et al., 2017) |
| *Paeonia suffruticosa* Andr. (Ranunculaceae; *Moutan cortex*)  and *Paeonia veitchii* Lynch (Ranunculaceae; *Paeoniae radix* rubra) | C57BL/6J mice | BWG ↓, FPINS ↓, FBG ↓, FBG ↓, TC ↓, TG ↓, LDL-C ↓, HDL-C ↑ | 1. Glucose metabolism ↑  2. Gut-liver axis: SREBP1c ↓, SREBP2 ↓ | Genus level: *Bacteroides* ↓, *Anaerotruncus* ↑, *Flavonifractor* ↑ | (Zhong et al., 2017) |
| *Panax ginseng* C. A. Mey. (Araliaceae; *Ginseng radix et rhizoma*) | C57BL/6J mice | BWG ↓, BW ↓, HOMA-ISI ↑ | —— | Species level: *Enterococcus faecalis* ↑ | (Quan et al., 2020) |
| Sanggua drink | C57BL/6J mice | HOMA-IR ↓, TC ↓, TG ↓, LDL-C ↓, HDL-C ↑ | 1. Pathway: PPARα ↑ | 1. Phylum level: F/B ↓, Verrucomicrobia ↑  2. Family level: Erysipelotrichaceae ↓, Christensenellaceae ↓, Verrucomicrobiaceae ↓, Rikenellaceae ↓ | (Zheng et al., 2020) |
| *Citrus aurantium* L. (Rutaceae; *Aurantii fructus*) | C57BL/6J mice | BW ↓, LW ↓, FBG ↓, FINS ↓, HOMA-IR ↓, TC ↓, TG ↓, NEFA ↓, LDL-C ↓, ALT ↓, AST ↓ | 1.Inflammation: LPS ↓, NF-κB/IκKα/β ↓, TNF-α ↓  2. Gut barrier: claudin-3 ↑, occludin ↑ | 1. Phylum level: Verrucomicrobia ↑, Bacteroidetes ↑, F/B ↓  2. Genus level: *Akkermansia* ↑, *Alistipes* ↑ | (Bai et al., 2019) |

**Abbreviations:** BW, body weight; LW, liver weight; ATW, adipose tissue weight; FBG, fasting blood glucose; TG, triglyceride; ALT, alanine aminotransferase; AST, aspartate aminotransferase; HDL-C, high-density lipoprotein cholesterol; NEFA, non esterified fatty acid; LDL-C, low-density lipoprotein cholesterol; BWG, body weight gain; HOMA-IR, homeostasis assessment of insulin resistance; MDA, malondialdehyde; FPG, fasting plasma glucose; INS, insulin; CCK, cholecystokinin; FINS, fasting insulin; FFA, free fatty acid; IR, insulin resistance; FPINS, fasting plasma insulin; HOMA-ISI, homeostasis assessment of insulin sensitivity index; LPS, lipopolysaccharide; NF-κB, nuclear factor kappa B; IκK, inhibitor of NF-κB kinase α; PPAR, peroxisome proliferator-activated receptor; SREBP, sterol-regulatory element binding protein, ZO-1, zonulaoccludens 1; TLR, toll-like receptor; IL, interleukin; TNF-α, tumor necrosis factor α; PAI-1, plasminogen activator inhibitor 1; MCP-1, monocyte chemoattractant protein 1; AA, amino acid; BA, bile acid; CA, cholic acid; SCFA, short-chain fatty acid; ACC, acetyl-CoA carboxylse; CPT, carnitine palmitoyl transferase; ATG, anti-thymocyte globulin; HSL, hormone sensitive lipase; ATGL, adipose triglyceride lipase; GLUT, glucose transporter; CXCL16, CXC chemokine ligand 16; SNX17, sorting nexin 17; Vldlr, very low density lipoprotein receptor; ACAA2, acetyl-coenzyme A acyltransferase 2; PGC1α, peroxisome proliferator-activated receptor gamma coactivator 1α; RBP4, retinol‐binding protein 4; AgRP, agouti-related peptide; NPY, neuropeptide Y; SIRT1, sirtuin 1; TFAM, mitochondrial transcription factor A; MCAD, medium-chain acyl-CoA dehydrogenase; KLB, β-klotho; FASN, fatty acid synthase; UCP, uncoupling protein 1; PRDM, positive regulatory domain containing; FGF, fibroblast growth factor; BAT, brown adipose tissue; FAO, fatty acid oxidation.

**Supplementary Table S4 Effect of herbal medicine on gut microbiota and its potential mechanism in the treatment of NAFLD.**

| Herbal medicine | Subject | Outcome | Potential mechanism | Changes in gut microbiota | Reference |
| --- | --- | --- | --- | --- | --- |
| *Ophiopogon japonicus* (L.f) Ker-Gawl. (Liliaceae; *Ophiopogonis radix*) | C57BL/6J mice | BW ↓, TG ↓, TC ↓, HDL-C ↓, LDL-C ↓, AST ↓, ALT ↓ | 1. Inflammation: IL-1β ↓, IL-6 ↓, TNF-α ↓  2. Gut barrier: occludin ↑, ZO-1 ↑, Muc-2 ↑  3. Lipid metabolism: SREBP1c ↓, FASN ↓, ACC-1 ↓, C/EBP ↓, PPARγ ↓ | 1. Phylum level: F/B ↓  2. Genus level: *Akkermansia* ↑, *Lachnospiraceae* NK4A136 group ↑, *Allobaculum* ↑ | (Zhang et al., 2022a) |
| Salvia-Nelumbinis naturalis formula | C57BL/6J mice | TG ↓, TC ↓, ALT ↓, AST ↓ | 1. BAs: GHCA ↓, T-ω-MCA ↓, GCA ↓, GDCA ↓, ω-MCA ↓  2. Receptors: FXR ↑, FGF15 ↑  3. Inflammation: CD68 ↓, IL-1β ↓, TNF-α ↓ | 1. Phylum level: F/B ↓  2. Family level: Prevotellaceae ↑, Lachnospiraceae ↑, Akkermansiaceae ↑  3. Genus level: *Clostridium sensu stricto* 1 ↓, *Alloprevotella* ↑, *Roseburia* ↑, *Lachnospiraceae* NK4A136 group ↑, *Akkermansia* ↑ | (Li et al., 2021a) |
| Shenling Baizhu powder | SD rats | BW ↓, LW ↓, ALT ↓, AST ↓, TG ↓, TC ↓ | 1. Inflammation: LPS ↓, IL-1β ↓, IL-1 ↓, TNF-α ↓  2. Signaling pathway: TLR4 ↓, MyD88 ↓, NLRP3 ↓ | 1. Phylum level: Actinobacteria ↑, Bacteroidetes ↑, F/B ↓  2. Genus level: *Bifidobacterium* ↑, *Phascolarctobacterium* ↓, *Desulfovibrio* ↓ | (Zhang et al., 2018) |
| *Poria cocos* (Schw.) Wolf (Polyporaceae; *Poria*) | C57BL/6J mice | MDA ↓, T- AOC ↑, AST ↓, ALT ↓, HDL-C ↑ | 1. Inflammation: LPS ↓  2. Signaling pathway: NF-κB ↓, CCL3 ↓, CCR1 ↓ | Genus level: *Faecalibaculu* ↑, *Tuzzerella* ↓, *Enterococcus* ↓ | (Tan et al., 2022) |
| Simiao decoction | C57BL/6J mice | BWG ↓, BW ↓, LW ↓, FBG ↓, IPGTT ↓, TG ↓, TC ↓, LDL-C ↓, AST ↓, ALT ↓ | 1. Lipid metabolism: Acly ↓, ACC ↓, FASN ↓, SCD-1 ↓, PPARα ↓, CD36 ↓  2. Inflammation: NLRP3 ↓, Il-1α ↓, IL-1β ↓  3. Gut barrier: ZO-1 ↑, occludin ↑ | 1. Phylum level: Verrucomicrobia ↑, Firmicutes ↓  2. Genus level: *Akkermansia* ↑  3. SCFA-producing bacteria: *Bifidobacterium* ↑, *Faecalibaculum* ↑  4. Species level: *Akkermansia muciniphila* ↑ | (Han et al., 2021) |
| Qushi Huayu decoction | C57BL/6J mice | BW ↓, ALT ↓, TG ↓ | 1. Inflammation: LPS ↓, LBP ↓, CD14 ↓, NF-κB ↓, CPT ↓  2. Gut barrier: ZO-1 ↑, occludin ↑, claudin-1 ↑ | 1. Phylum level: Firmicutes ↓  2. Genus level: *Romboutsia* ↓, *Rikenella* ↓, *Tyzzerella* ↓, *Parabacteroides* ↑ | (Leng et al., 2020) |
| *Cassia obtusifolia* L. (Leguminosae; *Cassiae semen*) | C57BL/6J mice | LW ↓, TC ↓, TG ↓, FFA ↓, LDL-C ↓, ALT ↓, AST ↓ | 1. Inflammation: TNF-α ↓, LPS ↓, IL-6 ↓, IL-10 ↑  2. Gut barriers: occludin ↑, ZO-1 ↑ | 1. Phylum level: Firmicutes ↓  2. Genus level: *Klebsiella* ↓, *Ruminococcus* ↑ | (Luo et al., 2021) |
| *Paeonia lactiflora* Pall. (Ranunculaceae; *Paeoniae radix* alba) and *Glycyrrhiza uralensis* Fisch. (Leguminosae; *Glycyrrhizae radix et rhizoma*) | C57BL/6J mice | BW ↓, BWG ↓, LW ↓, HOMA-IR ↓, FINS ↓  TG ↓, FFA ↓, ALT ↓ | 1. Inflammation: LPS ↓, MCP-1 ↓, IL-6 ↓  2. Gut barrier: ZO-1 ↑, occludin ↑  3. Gut-liver axis: TLR2 ↓, TLR9 ↓ | 1. Phylum level: Firmicutes ↓, F/B ↓, Proteobacteria ↓, Bacteroidetes ↑  2. Genus level: *Desulfovibrio* ↓ | (Chen et al., 2021b) |
| Dachaihu decoction | SD rats | BW ↓, MDA ↓, SOD ↑, GSH-Px ↑, TG ↓, TC ↓, AST ↓, ALT ↓, INS ↓, HOMA-IR ↓ | AAs: glycine ↓, serine ↓, threonine ↓, leucine ↓, 5-HPETE ↓, isoleucine ↓ | 1. Phylum level: F/B ↓  2. Genus: *Bacteroides* ↑, *Lactobacillus* ↑, *Akkermansia* ↑, *Turicibacter* ↑ | (Cui et al., 2020) |
| *Dendrobium officinale* Kimura & Migo (Orchidceae; *Dendrobii officinalis caulis*) | ICR mice | TC ↓, LDL-C ↓ | 1. Signaling pathway: TLR4 ↓, NF-κB ↓, MyD88 ↓  2. Inflammation: LPS ↓, TNF-α ↓, IL-6 ↓, IFN-γ ↓  3. Gut barrier: ZO-1 ↑, occlaudin ↑ | Genus level: *Desulfovibrio* ↓, *Rikenella* ↓ | (Lei et al., 2019) |
| Defatted walnut powder | C57BL/6 mice | BW ↓, LW ↓, FBG ↓  TG ↓, TC ↓, LDL-C ↓, MDA ↓, HDL-C ↑ | Signaling pathway: NF-κB ↓, MAPK ↓ | 1. Phylum level: Firmicutes ↓  2. Class level: Erysipelotrichia ↓  3. Family level: Prevotellaceae ↑  4. Genus level: *Bacteroides* ↑ | (Ren et al., 2021) |
| *Penthorum chinense* Pursh. (Saxifragaceae; *Penthori chinensis herba*) | C57BL/6J mice | LW ↓, FBG ↓, FINS ↓, HOMA-IR ↓, TC ↓, TG ↓, LDL-C ↓, ALT ↓, AST ↓ | 1. BA receptors: CYP7A1 ↑, CYP7B1 ↑, FXR ↑, BSEP ↑  2. BAs: T-β-MCA ↓, TDCA ↓, TCDCA ↓, TUDCA ↓, UDCA ↓, CDCA ↓, T-β-MCA ↑, DCA ↑ | 1. Phylum level: F/B ↓  2. Genus level: *Akkermansia* ↑, *Parabacteroides* ↑, *Clostridium* Ⅳ ↓ | (Li et al., 2022) |
| Qianggan formula | C57BL/6J mice | ALT ↓, AST ↓ | 1. Inflammation: TNF-α ↓, IL-1β ↓, TLR4 ↓, MyD88 ↓, NF-ĸB ↓, IκBα ↓  2. BA receptors: TGR5 ↓, FXR ↓, BSEP ↑  3. BAs: TDCA ↓, TLCA ↓ | Class level: Clostridia ↑ | (Li et al., 2020) |
| *Gynostemma pentaphyllum* (Thunb.) Makino (Cucurbitaceae; *Gynostemmae pentaphylli* herba) | C57BL/6 J mice | FBG ↓, HOMA-IR ↓, AST ↓, ALT ↓  Liver: TG ↓ | Liver lipid metabolism: HNF-4α ↑, SIRT1 ↑, PPARα ↑ | 1. Phylum level: Firmicutes ↓, Proteobacteria ↓  2. Genus level: *Eubacterium* ↓, *Clostridium* ↓, *Parasutterella* ↑ | (Jia et al., 2018) |
| Qushi  Huayu formula | SD rats | BW ↓, TG ↓, FFA ↓ | —— | 1. Phylum level: Actinobacteria ↑  2. Family level: Peptococcaceae ↓  3. Genus level: *Sporacetigenium* ↑, *Collinsella* ↑  4. Species level: *Collinsella aerofaciens* ↑ | (Yin et al., 2013) |
| Jiangan Jiangzhi pills | SD rats | BW ↓, TC ↓, TG ↓, ALT ↓, AST ↓ | Inflammation: IL-6 ↓, IL-1β ↓, TNF-α ↓ | 1. Phylum level: F/B ↓  2. Genus level: *Lactobacillus* ↑, *Bacteroides* ↑, *Turicibacter* ↑, *Akkermansia* ↑, *Staphylococcaceae* ↑, *Enterococcus* ↓ | (Zhao et al., 2022) |
| Erchen decoction | SD rats | BW ↓, MDA ↓, SOD ↓, GSH-Px ↑, TC ↓, TG ↓, ALT ↓, AST ↓ | 1. Inflammation: IL-6 ↓, IL-1β ↓, TNF-α ↓  2. BA: TCA ↑ | 1. Phylum level: F/B ↓  2. Family level: Lachnospiraceae ↑  3. Genus level: *Desulfovibrio* ↓, *Lactobacillus* ↑, *Dubosiella* ↑, *Akkermansia* ↑, *Intestinimonas* ↑ | (Miao et al., 2022) |
| Jiangan Xiaozhi decoction | SD rats | BWG ↓, ALT ↓, AST ↓, TC ↓, TG ↓ | 1. Gut barrier: occludin ↑, ZO-1 ↑  2. Inflammation: IL-6 ↓, IL-1β ↓, TNF-α ↓ | 1. Phylum level: F/B ↓  2. Genus level: *Lactobacillus* ↓, *Blautia* ↓, *Collinsella* ↓ | (Liao et al., 2020) |
| Jiangzhi granules | C57BL/6J mice | BW ↓, BWG ↓, MDA ↓, HOMA-IR ↓, SOD ↑, FFA ↓, ALT ↓, TC ↓, TG ↓ | 1. Gut barrier: occludin ↑, ZO-1 ↑, Muc-5 ↑  2. Liver lipid metabolism: PPARα ↑, CD14 ↑, TLR2 ↓, FABP5 ↓ | 1. Phylum level: Proteobacteria ↓, F/B ↓  2. Family level: Lachnospiraceae ↑, Desulfovibrionaceae ↓, Rikenellaceae ↓, Christensenellaceae ↓, Peptococcaceae ↓ | (Wang et al., 2021) |
| Chaihu Shugan powder | SD rats | BW ↓, TC ↓, TG ↓ | 1. Inflammation: TNF-α ↓, IL-1β ↓, IL-18 ↓, NF-кB ↓, NLRP3 ↓  2. SCFA: butyric acid ↑ | 1. Family level: Enterobacteriaceae ↓, Staphylococcaceae ↓  2. Genus level: *Veillonella* ↑, *Anaeroplasma* ↑ | (Liang et al., 2018b) |
| *Mallotus oblongifolius* (Miq.) Muell. Arg. (Euphorbiaceae *Mallotus folium*) | Wistar rats | TC ↓, LDL-C ↓, ALT ↓, GGT ↓ | Inflammation: IL-1β ↓, TNF-α↓, IL-6 ↓ | Phylum level: Actinobacteria ↑, Tenericutes ↑ | (Lin et al., 2022) |
| Shugan Xiaozhi decoction | SD rats | ALT ↓, AST ↓, TC ↓, TG ↓ | 1. Lipid metabolism: PPARα ↑  2. Gut barrier: ZO-1 ↑, occludin ↑, SIgA ↓  3. Inflammation: LPS ↓, IL-1β ↓, TNF-α ↓,  MCP-1 ↓ | Family level: Erysipelotrichaceae ↓, Desulfovibrionaceae ↓, Prevotellaceae ↑ | (Yang et al., 2022a) |
| Hongqi Jiangzhi formula | SD rats | BW ↓, TC ↓, TG ↓, LDL-C ↓ | 1. Gut barrier: occludin ↑ZO-1 ↑  2. Inflammation: LPS ↓, IL-1β↓, IL-18 ↓,  NLRP3 ↓ | 1. Class level: Epsilonproteobacteria ↓  2. Order level: Campylobacterales ↓  3. Family level: Helicobacteraceae ↓, Verrucomicrobiaceae ↓, Enterobacteriaceae ↓ | (Liang et al., 2018a) |

**Abbreviations:** BW, body weight; LW, liver weight; ALT, alanine aminotransferase; AST, aspartate aminotransferase; MDA, malondialdehyde; FFA, free fatty acid; TG, triglyceride; TC, total cholesterol; LDL-C, low-density lipoprotein cholesterol; HOMA-IR, homeostasis assessment of insulin resistance; HDL-C, high-density lipoprotein cholesterol; HOMA-ISI, homeostasis assessment of insulin sensitivity index; FBG, fasting blood glucose; FINS, fasting insulin; SOD, superoxide dismutase; T-AOC, total antioxidant capacity; IPGTT, intraperitoneal glucose tolerance test; INS, insulin; BWG, body weight gain; GGT, gamma-glutamyltransferase; LPS, lipopolysaccharide; IL, interleukin; TNF-α, tumor necrosis factor α; ZO-1, zonulaoccludens 1; MCP-1, monocyte chemoattractant protein 1; NLRP3, NOD-like receptor thermal protein domain associated protein 3; GSH-Px, glutathtone peroxtdase; SIgA, secretory immunoglobulin A; PPAR, peroxisome proliferator-activated receptor; NF-κB, nuclear factor kappa B; IκK, inhibitor of NF-κB kinase α; TLR, toll-like receptor; Muc, mucin; FABP, fatty acid binding protein; CD, leukocyte differentiation antigen; BA, bile acid; TCA, taurocholic acid; SCFA, short-chain fatty acid; CYP7A1, cholesterol 7α-hydroxylase; CYP7B1, oxysterol 7α-hydroxylase; FXR, farnesoid X receptor; MAPK, mitogen-activated protein kinase; TDCA, taurodeoxycholic acid; DCA, deoxycholic acid; UDCA, ursodeoxycholic acid; CDCA, chenodeoxycholic acid; TUDCA, tauroursodeoxycholic acid; TCDCA, taurochenodeoxycholic acid; T-β-MCA, tauro-β-muricholic acid; TDCA, taurodeoxycholic acid; TLCA, Taurolithocholic acid; SIRT1, sirtuin 1; MyD88, myeloid differentiation primary response gene 88; AA, amino acid; IFN, interferon; C/EBP, CCAAT/enhancer-binding protein; CCR1, chemokine receptor; BSEP, bile salt export pump; HNF-4α, hepatocyte nuclear factor 4α; SREBP, sterol regulatory element-binding protein; FASN, fatty acid synthase; SCD, stearoyl-CoA desaturase; ACC, acetyl-CoA carboxylse; GHCA, glycohyocholic acid; T-ω-MCA, tauro-ω-muricholic acid; GDCA, glycodeoxycholic acid; ω-MCA, ω-muricholic acid; GCA, glycocholic acid; FGF, fibroblast growth factor; Acly, ATP citrate lyase; CPT, carnitine palmitoyl transferase; CCL3, C-C motif chemokine ligand 3; LBP, lipopolysaccharide binding protein.

**Supplementary Table S5 Effect of herbal medicine on gut microbiota and its potential mechanism in the treatment of gout.**

| Herbal medicine | Subject | Outcome | Potential mechanism | Changes in gut microbiota | Reference |
| --- | --- | --- | --- | --- | --- |
| Simiao decoction | C57BL/6J mice | XOD ↓, MPO ↓, SUA ↓, BW ↓ | 1. Inflammation: IL-1β ↓, IL-9 ↓, IFN-γ ↓, MIP-1α ↓, MIP-1β ↓, IL-1α ↓, IL-6 ↓, IL-12 ↓, MCP-1 ↓, NLRP3 ↓, TNF-α↓, Caspase-8 ↓  2. Lipid metabolism: p-STAT3 ↓, APOB ↓, PPARα ↓, FN1 ↓, LPL ↓ | Genus level: *Prevotella* ↓, *Escherichia-Shigella* ↓, *Klebsiella* ↓, *Megamonas* ↓, *Enterococcus* ↓, *Phascolarctobacterium* ↓, *Prevotellaceae* Nk3b31 ↓ | (Lin et al., 2020) |
| Quzhuo Tongbi decoction | C57BL/6J mice | SUA ↓ | 1. Gut barrier: ZO-1 ↑, occludin ↑  2. Inflammation: NLRP3 ↑, IL-1β ↓, TNF-α ↓  3. Signaling pathway: GPR43 ↓, GLP-1 ↓ | 1. Phylum level: Bacteroidetes ↑, Epsilonbacteraeota ↓, Proteobacteria ↓  2. Butyrate producers: *Lachnospiraceae* A2 ↓, *Muribaculum* ↑, *Butyricicoccus* ↑ | (Wen et al., 2020) |
| *Astragalus membranaceus* (Fisch.) Bge. (Leguminosae; *Astragali radix*) | ICR mice | SUA ↓, BUN ↓, XOD ↓ | 1. Urate transporters: GLUT9 ↑, URAT1 ↑  2. Kidney inflammation: IL-1β ↓, IL-6 ↓, LPS ↓  3. Signaling pathway: TLR4 ↓, NF-кB ↓ | 1. Phylum level: Firmicutes ↓, Bacteroidetes ↑  2. Species level: *Lactobacillus intestinalis* ↑, *Bacillus mycoides* ↑  3. SCFA-producing bacteria: *Butyricimonas* ↑, *Collinsella* ↑ | (Wang et al., 2023) |
| *Carthamus tinctorius* L. (Compositae; *Carthami flos*) | SD rats | SUA ↓, BUN ↓, XOD ↓, MDA ↓, TG ↓, LDL-C ↓, SOD ↑, GSH-PX ↑ | 1. SCFAs ↑  2. AAs: decanoylcarnitine ↑, L-aspartic acid ↑, betaine ↑, L-glutamine ↓, L-leucine ↓, L-asparagine ↓, L-isoleucine ↓, L-tyrosine ↓ | Genus level: *Holdemania* ↓, *Parabacteroides-goldsteinii* ↓ | (Chen et al., 2022) |
| *Astragalus membranaceus* (Fisch.) Bge. (Leguminosae; *Astragali radix*) | SD rats | SUA ↓, ALT ↓, AST ↓, IBIL ↓, TG ↓, BW ↓ | Cholic acid ↓ | 1. Phylum level: Firmicutes ↓, F/B ↓  2. Genus level: *Blautia* ↑  3. Species level: *Lactobacillus johnsonii* ↓  4. SCFA-producing bacteria: *Lachnospiraceae* ↑ | (Zhang et al., 2022c) |
| *Thlaspi arvense* L. (Cruciferae; *Thlaspi herba*) | Kunming mice | —— | —— | 1. Family level: Lactobacillaceae ↑, Muribaculaceae ↓  2. Genus level: *Helicobacter* ↑, *Prevotellaceae* UCG 001 ↓, *Parabacteroides* ↓ | (Kang et al., 2021) |
| *Phellinus igniarius* (L. ex Fr.) Quel. (Polyporaceae; *Phellinus*) | SD rats | SUA ↓, XOD ↓ | Renal function: creatinine level | Genus level: *Lactobacillus* ↑, *Turicibacter* ↑, *Lachnospiraceae* NK4A136 group ↑, *Prevotella* 9 ↓, *Romboutsia* ↓ | (Li et al., 2021e) |
| *Cichorium intybus* L. (Compositae; *Cichorii herba cichorii radix*) | SD rats | SUA ↓ | 1. Inflammation: LPS ↓  2. Gut barrier: SIgA ↑ | 1. Genus level: *Bifidobacterium* ↑  2. Species level: *Escherichia coli* ↓, *Enterococcus faecalis* ↓ | (Wang et al., 2018) |

**Abbreviations:** XOD, xanthine oxidase; MPO, myeloperoxidase; SUA, serum uric acid; BW, body weight; BUN, blood urea nitrogen; MDA, malondialdehyde; TG, triglyceride; TC, total cholesterol; LDL-C, low-density lipoprotein cholesterol; GSH-Px, glutathtone peroxtdase; ALT, glutathione aminotransferase; AST, glutathione transaminase; IBIL, indirect bilirubin; IL, interleukin; TNF-α, tumor necrosis factor α; IFN, interferon; MIP, macrophage inflammatory protein; MCP-1, monocyte chemoattractant protein 1; NLRP3, NOD-like receptor thermal protein domain associated protein 3; STAT, signal transducerand activator of transcription; APOB, apolipoprotein B; PPAR, peroxisome proliferator-activated receptor; IFN, interferon; LPL, lipoprotein lipase; ZO-1, zonula occludens 1; GPR, G protein-coupled receptor; GLP-1, glucagon-like peptide-1; GLUT, glucose transporter; URAT1, urate transporter 1; LPS, lipopolysaccharide; TLR, toll-like receptor; NF-κB, nuclear factor kappa B; SCFA, short-chain fatty acid; SIgA, secretory immunoglobulin A.

**Supplementary Table S6 Effect of herbal medicine on gut microbiota and its potential mechanism in the treatment of hyperlipidemia.**

| Herbal medicine | Subject | Outcome | Potential mechanism | Changes in gut microbiota | Reference |
| --- | --- | --- | --- | --- | --- |
| Shanmei capsule | C57BL/6J mice | TG ↓, TC ↓, LDL-C ↓, FINS ↑ | Glycerophospholipid metabolism  Linolenic acid metabolism  Arachidonic acid metabolism | 1. Phylum level: F/B ↓  2. Genus level: *Lachnospiraceae* NK4B4 group ↓, *Olsenella* ↓, *Robinsoniella* ↓, *Ruminococcus* 1 ↓, *Tyzzerella* ↓ | (Du et al., 2022) |
| *Lindera aggregate* (Sims) Kos-term. (Lauraceae; *Linderae radix*) | SD rats | TC ↓, TG ↓, LDL-C ↓, ALT ↓, AST ↓ | 1. BAs: TUDCA ↑, TCA ↑, TUDCA ↑, THDCA ↓  2. BA receptors: CYP7A1 ↑ | Phylum level: F/B ↓ | (Jiang et al., 2021a) |
| Shener Jiangzhi formula | SD rats | TC ↓, TG ↓, LDL-C ↓, HDL-C ↑ | 1. AAs: tryptophan, arginine  2. NLRP3 | 1. Phylum level: F/B ↓, Proteobacteria ↓  2. Genus level: *Akkermansia* ↑, *Roseburia* ↑, *Helicobacter* ↑ | (Zhang et al., 2022b) |
| *Alisma orientale* (Sam.) Juz. (Alismataceae; *Alismatis rhizoma*) | SD rats | TC ↓, LDL-C ↓ | Liver tissue damage ↓ | 1. Phylum level: Verrucomicrobia ↑, Bacteroidetes ↑, Tenericutes ↑, Firmicutes ↓  2. Genus level: *Bacteroides* ↑, *Akkermansia* ↑ | (Li et al., 2019c) |
| Jieyu Qutan Huazhuo formula | Wistar mice | TC ↓, TG ↓, LDL-C ↓, HDL-C ↑ | Improve liver cell and ileal mucosal tissue damage | 1. Class level: Bacteroidia ↑  2. Order level: Erysipelotrichales ↓  3. Family level: Ruminococcaceae ↑  4. Genus level: *Bacteroides* S24-7 ↑,  *Rumencoccus* UCG 005 ↑ | (Li et al., 2021c) |
| Tianhuang formula | C57BL/6J mice | TC ↓, TG ↓ | 1. BA receptors: FXR ↓, FGF15 ↓, CYP7A1 ↑  2. BAs: T-β-MCA ↑  3. BSH activity ↓ | 1. Phylum level: Proteobacteria ↓  2. Family level: Enterobacteriaceae ↓  3. Genus level: *Escherichia* ↓, *Bacteroides* ↑, *Akkermansia* ↑ | (Yang et al., 2022b) |
| Wuwei Qingzhuo powder | ApoE^-/-^ mice | TC ↓, TG ↓, LDL-C ↓ | 1. BAs: biosynthesis ↑  2. BA receptor: CYP7A1 | 1. Phylum level: Firmicutes ↓, Bacteroides ↑  2. Genus level: *Bilophila* ↑, *Roseburia* ↑, *Dubosiella* ↑ | (Ge et al., 2022) |
| Yinian Kangbao tea | ApoE^-/-^ mice | LW ↓, TG ↓, ALT ↓, TG ↓, TC ↓, AST ↑, HDL-C ↑ | —— | 1. Phylum level: Firmicutes ↓, Actinobacteria ↑, Verrucomicrobia ↑, Bacteroidetes ↑  2. Genus level: *Turicibacter* ↑, *Lactobacillus* ↑, *Bifidobacterium* ↑, *Akkermansia* ↑, *Romboutsia* ↓ | (Li et al., 2019b) |
| *Polyporus frondosus* (Dicks.) Fr. (Polyporaceae; *Fructificatio polypori frondosi*) | Wistar rats | TG ↓, TC ↓, LDL-C ↓, FFA ↓, MDA ↓, ALT ↓, AST ↓, GSH-PX ↑, SOD ↑, HDL-C ↑ | Lipid metabolism: ACAT2 ↓, PPARγ ↑, CYP7A1 ↑, CAT ↑ | Genus level: *Helicobater* ↑, *Intestinimonas* ↑, *Barnesiella* ↑, *Parasutterella* ↑, *Ruminococcus* ↑, *Butyricicoccus* ↓ | (Li et al., 2019a) |
| *Coptis chinensis* Franch. (Ranunculaceae; *Coptidis rhizoma*) | C57BL/6J mice | BW ↓, TG ↓, TC ↓, LDL-C ↓, LW ↓ | 1. Inflammation: TNF-α ↓, IL-1β ↓, IL-6 ↓  2. Gut barrier: occludin ↑, ZO-1 ↑, claudin-2 ↑, claudin-3 ↑, claudin-4 ↑ | 1. Phylum level: Firmicutes ↓, Actinobacteri ↓, Candidatus-Melainabacteria ↓, *Verrucomicrobia* ↑  2. Species levels: *Akkermansia muciniphila* ↑, *Lactobacillus johnsonii* ↑, *Klebsiella variicola* ↑, *Muribaculum intestinale* ↑ | (Yang et al., 2022c) |
| Congxin Lunzhi formula | ApoE^-/-^ mice | BW ↓, TC ↓, TG ↓, LDL-C ↓, HDL-C ↑ | Liver steatosis ↓ | 1. Phylum level: Bacteroidetes ↑, Firmicutes ↓  2. Class level: Bacteroidia ↓, Erysipelotrichia ↓  3. Family level: Erysipelotrichaceae ↓, Lachnospiraceae ↑, Ruminococcaceae ↑ | (Zhang et al., 2020) |
| *Bletilla striata* (Thunb.) Reichb.f. (Orchidaceae; *Bletillae rhizoma*) | C57BL/6J mice | HOMA-IR ↓, FINS ↓, TC ↓, TG ↓, HDL-C ↑ | 1. Lipid metabolic: MCP-1 ↓  2. Gut barrier: Muc-3 ↑  3. BAs: DCA ↓, TUDCA ↑, TDCA ↑, TCDCA ↑, TCA ↑, T-α-MCA ↑, T-β-MCA ↑, CA ↑  4. SCFA: acetic acid ↑  5. AA: tryptamine ↓ | Genus level: *Lachnospiraceae* NC2004 group ↓, *Enterococcus* ↓, *Ruminococcus* 1 ↓, *Eubacterium nodatum* group ↑, *Butyricicoccus* ↑, *Coriobacteriaceae* UCG 002 ↑, *Blautia* ↑, *Erysipelatoclostridium* ↑, *Faecalibaculum* ↑ | (Hu et al., 2020) |

**Abbreviations:** TC, total cholesterol; TG, triglyceride; LDL-C, low-density lipoprotein cholesterol; FINS, fasting insulin; ALT, glutathione aminotransferase; AST, glutathione transaminase; HDL-C, high-density lipoprotein cholesterol; FFA, free fatty acid; MDA, malondialdehyde; GSH-Px, glutathtone peroxtdase; SOD, superoxide dismutase; LW, liver weight; CYP7A1, cholesterol 7α-hydroxylase; NLRP3, NOD-like receptor thermal protein domain associated protein 3; FXR, farnesoid X receptor; FGF, fibroblast growth factor; ACAT2, acetyl-coenzyme A acetyltransferase 2; PPAR, peroxisome proliferator-activated receptor; IL, interleukin; ZO-1, zonulaoccludens 1; MCP-1, monocyte chemoattractant protein 1; TDCA, taurodeoxycholic acid; DCA, deoxycholic acid; GCA, glycocholic acid; CDCA, chenodeoxycholic acid; THDCA, taurohyodeoxycholic acid; TUDCA, tauroursodeoxycholic acid; TCDCA, taurochenodeoxycholic acid; TCA, taurocholic acid; T-ω-MCA, tauro-ω-muricholic acid; T-β-MCA, tauro-β-muricholic acid; CAT, catalase.

**References**

Ansari, A., Bose, S., Yadav, M.K., Wang, J.H., Song, Y.K., Ko, S.G., Kim, H., (2016). CST, an herbal formula, exerts anti-obesity effects through brain-gut-adipose tissue axis modulation in high-fat diet fed mice. *Molecules*. 21 (11), 1522. doi: 10.3390/molecules21111522

Bai, J., Zhu, Y., Dong, Y., (2016). Response of gut microbiota and inflammatory status to bitter melon (*Momordica charantia* L.) in high fat diet induced obese rats. *Journal of Ethnopharmacology*. 194, 717-726. doi: 10.1016/j.jep.2016.10.043

Bai, Y.F., Wang, S. W., Wang, X.X., Weng, Y.Y., Fan, X.Y., Sheng, H., Zhang, F., (2019). The flavonoid-rich Quzhou Fructus Aurantii extract modulates gut microbiota and prevents obesity in high-fat diet-fed mice. *Nutr Diabetes.* 9 (1), 30. doi: 10.1038/s41387-019-0097-6

Cao, Y., Yao, G., Sheng, Y., Yang, L., Wang, Z., Yang, Z., Zhang, Y., (2019). JinQi Jiangtang tablet regulates gut microbiota and improve insulin sensitivity in type 2 diabetes mice. *J Diabetes Res.* 2019, 1872134. doi: 10.1155/2019/1872134

Chang, C.J., Lin, C.S., Lu, C.C., Martel, J., Ko, Y.F., Ojcius, D.M., Lai, H.C., (2017). Corrigendum: *Ganoderma lucidum* reduces obesity in mice by modulating the composition of the gut microbiota. *Nat Commun*. 8, 16130. doi: 10.1038/ncomms16130

Chen, H., Yao, Y., Wang, W., Wang, D., (2021a). Ge-Gen-Jiao-Tai-Wan affects type 2 diabetic rats by regulating gut microbiota and primary bile acids. *Evid Based Complement Alternat Med*. 2021, 5585952. doi: 10.1155/2021/5585952

Chen, L., Kan, J., Zheng, N., Li, B., Hong, Y., Yan, J., Li, H., (2021b). A botanical dietary supplement from white peony and licorice attenuates nonalcoholic fatty liver disease by modulating gut microbiota and reducing inflammation. *Phytomedicine*. 91, 153693. doi: 10.1016/j.phymed.2021.153693

Chen, M., Liao, Z., Lu, B., Wang, M., Lin, L., Zhang, S., Xie, Z., (2018). Huang-Lian-Jie-Du-Decoction ameliorates hyperglycemia and insulin resistant in association with gut microbiota modulation. *Front Microbiol*. 9, 2380. doi: 10.3389/fmicb.2018.02380

Chen, N., Wang, R., Li, H., Wang, W., Wang, L., Yin, X., Yang, B., (2022). Flavonoid extract of saffron by‐product alleviates hyperuricemia via inhibiting xanthine oxidase and modulating gut microbiota. *Phytotherapy Research*. 36 (12), 4604-4619. doi: 10.1002/ptr.7579

Cui, H., Li, Y., Wang, Y., Jin, L., Yang, L., Wang, L., Liu, X., (2020). Da-chai-hu decoction ameliorates high fat diet-induced nonalcoholic fatty liver disease through remodeling the gut microbiota and modulating the serum metabolism. *Frontiers in Pharmacology*. 11. doi: 10.3389/fphar.2020.584090

Cui, H.X., Zhang, L.S., Luo, Y., Yuan, K., Huang, Z.Y., Guo, Y., (2019). A purified anthraquinone-glycoside preparation from Rhubarb ameliorates type 2 diabetes mellitus by modulating the gut microbiota and reducing inflammation. *Front Microbiol*. 10, 1423. doi: 10.3389/fmicb.2019.01423

Dong, W., Mao, Y., Xiang, Z., Zhu, J., Wang, H., Wang, A., Gu, Y., (2022). Traditional Chinese medicine formula Jian Pi Tiao Gan Yin reduces obesity in mice by modulating the gut microbiota and fecal metabolism. *Evid Based Complement Alternat Med*. 2022, 9727889. doi: 10.1155/2022/9727889

Du, L., Wang, Q., Ji, S., Sun, Y., Huang, W., Zhang, Y., Jin, H., (2022). Metabolomic and microbial remodeling by Shanmei capsule improves hyperlipidemia in high fat food-induced mice. *Front Cell Infect Microbiol*. 12, 729940. doi: 10.3389/fcimb.2022.729940

Fu, M., Yu, H., Bao, T., (2022). Anti-obesity and lipid-lowering mechanism of *Corydalis Bungeanae* herba: Based on intestinal microflora and metabolomics. *China Journal of Chinese Materia Medica*. 47 (11), 3049-3058. doi: 10.19540/j.cnki.cjcmm.20211125.701.

Gao, K., Yang, R., Zhang, J., Wang, Z., Jia, C., Zhang, F., Li, S., Wang, J., Murtaza, G., Xie, H., Zhao, H., Wang, W., Chen, J., (2018). Effects of Qijian mixture on type 2 diabetes assessed by metabonomics, gut microbiota and network pharmacology. *Pharmacol Res* 130, 93-109. doi: 10.1016/j.phrs.2018.01.011

Ge, S., Liao, C., Su, D., Li, Z., Tu, Y., (2022). Wuwei Qingzhuo San ameliorates hyperlipidemia in mice fed with HFD by regulating metabolomics and intestinal flora composition. *Front Pharmacol*. 13, 842671. doi: 10.3389/fphar.2022.842671

Gong, S., Ye, T., Wang, M., Wang, M., Li, Y., Ma, L., Qian, J., (2020). Traditional Chinese medicine formula Kang Shuai Lao Pian improves obesity, gut dysbiosis, and fecal metabolic disorders in high-fat diet-fed mice. *Front Pharmacol*. 11, 297. doi: 10.3389/fphar.2020.00297

Han, R., Qiu, H., Zhong, J., Zheng, N., Li, B., Hong, Y., Li, H., (2021). Si Miao formula attenuates non-alcoholic fatty liver disease by modulating hepatic lipid metabolism and gut microbiota. *Phytomedicine*. 85, 153544. doi: 10.1016/j.phymed.2021.153544

Hu, B., Ye, C., Leung, E.L., Zhu, L., Hu, H., Zhang, Z., Liu, H., (2020). Bletilla striata oligosaccharides improve metabolic syndrome through modulation of gut microbiota and intestinal metabolites in high fat diet-fed mice. *Pharmacol Res*. 159, 104942. doi: 10.1016/j.phrs.2020.104942

Hussain, A., Yadav, M.K., Bose, S., Wang, J.H., Lim, D., Song, Y.K., Kim, H., (2016). Daesiho-Tang Is an effective herbal formulation in attenuation of obesity in mice through alteration of gene expression and modulation of intestinal microbiota. *PloS One*. 11 (11), e0165483. doi: 10.1371/journal.pone.0165483

Jia, N., Lin, X., Ma, S., Ge, S., Mu, S., Yang, C., Zhao, J., (2018). Amelioration of hepatic steatosis is associated with modulation of gut microbiota and suppression of hepatic miR-34a in *Gynostemma pentaphylla* (Thunb.) Makino treated mice. *Nutrition & Metabolism*. 15. doi: 10.1186/s12986-018-0323-6

Jiang, T., Xu, C., Liu, H., Liu, M., Wang, M., Jiang, J., Lou, Z., (2021a). *Linderae Radix* ethanol extract alleviates diet-induced hyperlipidemia by regulating bile acid metabolism through gut microbiota. *Frontiers in Pharmacology*. 12. doi: 10.3389/fphar.2021.627920

Jiang, Y., Zhang, N., Zhou, Y., Zhou, Z., Bai, Y., Strappe, P., Blanchard, C., (2021b). Manipulations of glucose/lipid metabolism and gut microbiota of resistant starch encapsulated *Ganoderma lucidum* spores in T2DM rats. *Food Sci Biotechnol*. 30 (5), 755-764. doi: 10.1007/s10068-021-00908-w

Kang, X., Hao, H., Mu, C., Lu, T., Wang, X., Tang, L., (2021). Effect of different extracts of thlaspi herba on gut microbiota of hyperuricemia mice. *Chinese Journal of Experimental Traditional Medical Formulae*. 27 (17), 132-138. doi: 10.13422/j.cnki.syfjx.20211249

Lei, S.S., Li, B., Chen, Y.H., He, X., Wang, Y.Z., Yu, H.H., Chen, S.H., (2019). *Dendrobii Officinalis*, a traditional Chinese edible and officinal plant, accelerates liver recovery by regulating the gut-liver axis in NAFLD mice. *Journal of Functional Foods*. 61. doi: 10.1016/j.jff.2019.103458

Leng, J., Huang, F., Hai, Y., Tian, H., Liu, W., Fang, Y., Peng, J., (2020). Amelioration of non-alcoholic steatohepatitis by Qushi Huayu decoction is associated with inhibition of the intestinal mitogen-activated protein kinase pathway. *Phytomedicine*. 66. doi: 10.1016/j.phymed.2019.153135

Li, C., Zhou, W., Li, M., Shu, X., Zhang, L., Ji, G., (2021a). *Salvia-Nelumbinis naturalis* extract protects mice against MCD diet-induced steatohepatitis via activation of colonic FXR-FGF15 pathway. *Biomed Pharmacother*. 139, 111587. doi: 10.1016/j.biopha.2021.111587

Li, L., Guo, W.L., Zhang, W., Xu, J.X., Qian, M., Bai, W.D., Lv, X.C., (2019a). *Grifola frondosa* polysaccharides ameliorate lipid metabolic disorders and gut microbiota dysbiosis in high-fat diet fed rats. *Food Funct*. 10 (5), 2560-2572. doi: 10.1039/c9fo00075e

Li, L., Shi, M., Salerno, S., Tang, M., Guo, F., Liu, J., Fu, P., (2019b). Microbial and metabolomic remodeling by a formula of Sichuan dark tea improves hyperlipidemia in apoE-deficient mice. *PloS One*. 14 (7), e0219010. doi: 10.1371/journal.pone.0219010

Li, L., Xu, X., Lu, X., Zhang, Y., Lin, W., Xu, R., (2019c). Effects of *Alisma orientale* on the diversity of gut microbiota in rats fed on high-fat and high-sugar diet. *Front Chinese Journal of Microecology*. 31 (4), 396-401. doi: 10.13381/j.cnki.cjm.201904005

Li, M., Ding, L., Hu, Y. L., Qin, L. L., Wu, Y., Liu, W., Liu, T. H., (2021b). Herbal formula LLKL ameliorates hyperglycaemia, modulates the gut microbiota and regulates the gut-liver axis in Zucker diabetic fatty rats. *J Cell Mol Med*. 25 (1), 367-382. doi: 10.1111/jcmm.16084

Li, N., Wu, Y., Duan, J., Zheng, X., Yao, K., (2021c). Explore effect of Jieyu Qutan Huazhuo prescription on gut-liver axis of rats with high-fat diet based on 16S rDNA sequencing. *Chinese Journal of Experimental Traditional Medical Formulae*. 27 (9), 77-85. doi: 10.13422/j.cnki.syfjx.20210327

Li, Q., Hu, J., Nie, Q., Chang, X., Fang, Q., Xie, J., Nie, S., (2021d). Hypoglycemic mechanism of polysaccharide from *Cyclocarya paliurus* leaves in type 2 diabetic rats by gut microbiota and host metabolism alteration. *Sci China Life Sci*. 64 (1), 117-132. doi: 10.1007/s11427-019-1647-6

Li, Q., Li, M., Li, F., Zhou, W., Dang, Y., Zhang, L., Ji, G., (2020). Qiang-Gan formula extract improves non-alcoholic steatohepatitis via regulating bile acid metabolism and gut microbiota in mice. *J Ethnopharmacol*. 258, 112896. doi: 10.1016/j.jep.2020.112896

Li, X., Chu, F., Jiang, S., Jin, X., (2021e). Preliminary study on effect of *Phellinus igniarius* ethanol extract on serumuric acid metabolism and gut microbiome in rats. *China Journal of Chinese Materia Medica*. 46 (1), 177-182. doi: 10

Li, X., Zhao, W., Xiao, M., Yu, L., Chen, Q., Hu, X., Wu, X., (2022). *Penthorum chinense* Pursh. extract attenuates non-alcholic fatty liver disease by regulating gut microbiota and bile acid metabolism in mice. *J Ethnopharmacol*. 294, 115333. doi: 10.1016/j.jep.2022.115333

Liang, S., Zhang, Y., Deng, Y., He, Y., Liang, Y., Liang, Z., Yang, Q., (2018a). The potential effect of Chinese herbal formula Hongqijiangzhi Fang in improving NAFLD: focusing on NLRP3 inflammasome and gut microbiota. *Evid Based Complement Alternat Med*. 2018, 5378961. doi: 10.1155/2018/5378961

Liang, Y., Zhang, Y., Deng, Y., Liang, S., He, Y., Chen, Y., Yang, Q., (2018b). Chaihu-Shugan-San decoction modulates intestinal microbe dysbiosis and alleviates chronic metabolic inflammation in NAFLD rats via the NLRP3 inflammasome pathway. *Evid Based Complement Alternat Med*. 2018, 9390786. doi: 10.1155/2018/9390786

Liao, J., Xie, X., Gao, J., Zhang, Z., Qv, F., Cui, H., Wang, H., (2020). Jian-Gan-Xiao-Zhi decoction ameliorates nonalcoholic fatty liver disease through modulating gut microbiota, decreasing gut permeability, and alleviating liver inflammation. *Research Square*. doi: 10.21203/rs.3.rs-122886/v1

Lin, D., Ding, Y., Cheng, Y., Chen, Y., Tang, Y., Wu, X., Cheng, Y., (2022). Efficacy and mechanism of *Mallotus furetianus* Mull. Arg. extract on nonalcoholic fatty liver disease. *Evid Based Complement Alternat Med*. 2022, 4897463. doi: 10.1155/2022/4897463

Lin, X., Shao, T., Huang, L., Wen, X., Wang, M., Wen, C., He, Z., (2020). Simiao decoction alleviates gouty arthritis by modulating proinflammatory cytokines and the gut ecosystem. *Front Pharmacol*. 11, 955. doi: 10.3389/fphar.2020.00955

Luo, H., Wu, H., Wang, L., Xiao, S., Lu, Y., Liu, C., Tang, L., (2021). Hepatoprotective effects of *Cassiae Semen* on mice with non-alcoholic fatty liver disease based on gut microbiota. *Commun Biol*. 4 (1), 1357. doi: 10.1038/s42003-021-02883-8

Miao, J., Guo, L., Cui, H., Wang, L., Zhu, B., Lei, J., Zhang, Z., (2022). Er-Chen decoction alleviates high-fat diet-induced nonalcoholic fatty liver disease in rats through remodeling gut microbiota and regulating the serum metabolism. *Evidence-Based Complementary and Alternative Medicine*. doi: 10.1155/2022/6221340

Nie, Q., Hu, J., Gao, H., Fan, L., Chen, H., Nie, S., (2019). Polysaccharide from *Plantago asiatica* L. attenuates hyperglycemia, hyperlipidemia and affects colon microbiota in type 2 diabetic rats. *Food Hydrocolloids*. 86, 34-42. doi: 10.1016/j.foodhyd.2017.12.026

Quan, L.H., Zhang, C., Dong, M., Jiang, J., Xu, H., Yan, C., Jin, W., (2020). Myristoleic acid produced by enterococci reduces obesity through brown adipose tissue activation. Gut. 69 (7), 1239-1247. doi: 10.1136/gutjnl-2019-319114

Ren, S. M., Zhang, Q.Z., Chen, M.L., Jiang, M., Zhou, Y., Xu, X.J., Liu, X.Q., (2021). Anti-NAFLD effect of defatted walnut powder extract in high fat diet-induced C57BL/6 mice by modulating the gut microbiota. *J Ethnopharmacol*. 270, 113814. doi: 10.1016/j.jep.2021.113814

Shao, J., Liu, Y., Wang, H., Luo, Y., Chen, L., (2020). An integrated fecal microbiome and metabolomics in T2DM rats reveal antidiabetes effects from host-microbial metabolic axis of EtOAc extract from *Sophora flavescens*. *Oxid Med Cell Longev.* 2020, 1805418. doi: 10.1155/2020/1805418

Sun, R. X., Huang, W. J., Xiao, Y., Wang, D. D., Mu, G. H., Nan, H., Zhao, J. X., (2022a). Shenlian (SL) decoction, a traditional Chinese medicine compound, may ameliorate blood glucose via mediating the gut microbiota in db/db mice. *J Diabetes Res.* 2022, 7802107. doi: 10.1155/2022/7802107

Sun, Y., Qu, W., Liao, J., Chen, L., Cao, Y., Li, H., (2022b). Jiangtangjing ameliorates type 2 diabetes through effects on the gut microbiota and cAMP/PKA pathway. *Traditional Medicine Research.* 7 (1). doi: 10.53388/tmr20211112251

Tan, Y.Y., Yue, S.R., Lu, A.P., Zhang, L., Ji, G., Liu, B.C., Wang, R.R., (2022). The improvement of nonalcoholic steatohepatitis by *Poria cocos* polysaccharides associated with gut microbiota and NF-kappaB/CCL3/CCR1 axis. *Phytomedicine*. 103, 154208. doi: 10.1016/j.phymed.2022.154208

Wang, H.Y., Guo, L.X., Hu, W.H., Peng, Z.T., Wang, C., Chen, Z.C., Tsim, K. W. K., (2019a). Polysaccharide from tuberous roots of *Ophiopogon japonicus* regulates gut microbiota and its metabolites during alleviation of high-fat diet-induced type-2 diabetes in mice. *Journal of Functional Foods*. 63. doi: 10.1016/j.jff.2019.103593

Wang, R., Lin, F., Ye, C., Aihemaitijiang, S., Halimulati, M., Huang, X., Zhang, Z., (2023). Multi-omics analysis reveals therapeutic effects of Bacillus subtilis-fermented *Astragalus membranaceus* in hyperuricemia via modulation of gut microbiota. *Food Chem*. 399, 133993. doi: 10.1016/j.foodchem.2022.133993

Wang, R. R., Zhang, L. F., Chen, L. P., Wang, J. Y., Zhang, L., Xu, Y. S., Liu, B. C., (2021). Structural and functional modulation of gut microbiota by Jiangzhi granules during the amelioration of nonalcoholic fatty liver disease. *Oxid Med Cell Longev.* 2021, 2234695. doi: 10.1155/2021/2234695

Wang, X., Chen, D., Li, Y., Zhao, S., Chen, C., Ning, D., (2019b). Alleviating effects of walnut green husk extract on disorders of lipid levels and gut bacteria flora in high fat diet-induced obesity rats. *Journal of Functional Foods*. 52, 576-586. doi: 10.1016/j.jff.2018.11.022

Wang, Y., Lin, Z., Bian, M., Zhang, B., (2018). Effects on intervention of intestinal barrier with uyghur medicine *Cichorium intybus* Linn in hyperuricemia. *China Journal of Traditional Chinese Medicine and Pharmacy.* 33 (5), 1718-1723.

Wei, X., Tao, J., Xiao, S., Jiang, S., Shang, E., Zhu, Z., Duan, J., (2018). Xiexin Tang improves the symptom of type 2 diabetic rats by modulation of the gut microbiota. *Sci Rep*. 8 (1), 3685. doi: 10.1038/s41598-018-22094-2

Wen, X., Lou, Y., Song, S., He, Z., Chen, J., Xie, Z., Shao, T., (2020). Qu-Zhuo-Tong-Bi decoction alleviates gouty arthritis by regulating butyrate-producing bacteria in mice. *Front Pharmacol*. 11, 610556. doi: 10.3389/fphar.2020.610556

Wu, L., Yan, Q., Chen, F., Cao, C., Wang, S., (2021a). *Bupleuri radix* extract ameliorates impaired lipid metabolism in high-fat diet-induced obese mice via gut microbia-mediated regulation of FGF21 signaling pathway. *Biomed Pharmacother*. 135, 111187. doi: 10.1016/j.biopha.2020.111187

Wu, R., Zhao, D., An, R., Wang, Z., Li, Y., Shi, B., Ni, Q., (2019). Linggui Zhugan formula improves glucose and lipid levels and alters gut microbiota in high-fat diet-induced diabetic mice. *Front Physiol.* 10, 918. doi: 10.3389/fphys.2019.00918

Wu, S., Zuo, J., Cheng, Y., Zhang, Y., Zhang, Z., Wu, M., Tong, H., (2021b). Ethanol extract of *Sargarsum fusiforme* alleviates HFD/STZ-induced hyperglycemia in association with modulation of gut microbiota and intestinal metabolites in type 2 diabetic mice. *Food Res Int.* 147, 110550. doi: 10.1016/j.foodres.2021.110550

Xiao, S., Liu, C., Chen, M., Zou, J., Zhang, Z., Cui, X., Duan, J., (2020). *Scutellariae radix* and *Coptidis rhizoma* ameliorate glycolipid metabolism of type 2 diabetic rats by modulating gut microbiota and its metabolites. *Appl Microbiol Biotechnol.* 104 (1), 303-317. doi: 10.1007/s00253-019-10174-w

Xu, T., Ge, Y., Du, H., Li, Q., Xu, X., Yi, H., Zhang, Y., (2021). *Berberis kansuensis* extract alleviates type 2 diabetes in rats by regulating gut microbiota composition. *J Ethnopharmacol*. 273, 113995. doi: 10.1016/j.jep.2021.113995

Xu, X., Gao, Z., Yang, F., Yang, Y., Chen, L., Han, L., Wang, J., (2020). Antidiabetic effects of Gegen Qinlian decoction via the gut microbiota are attributable to its key ingredient berberine. *Genomics Proteomics Bioinformatics*. 18 (6), 721-736. doi: 10.1016/j.gpb.2019.09.007

Yan, Z., Wu, H., Zhou, H., Chen, S., He, Y., Zhang, W., Su, W., (2020). Integrated metabolomics and gut microbiome to the effects and mechanisms of naoxintong capsule on type 2 diabetes in rats. *Sci Rep.* 10 (1), 10829. doi: 10.1038/s41598-020-67362-2

Yang, H., Feng, L., Xu, L., Jiang, D., Zhai, F., Tong, G., Xing, Y., (2022a). Intervention of Shugan Xiaozhi decoction on nonalcoholic fatty liver disease via mediating gut-liver axis. *Biomed Res Int*. 2022, 4801695. doi: 10.1155/2022/4801695

Yang, L., Chen, K., Luo, D., Guo, J., (2022b). Efficacy and mechanism of Tianhuang formula in regulating lipid metabolism disorders in senile mice based on gut microbiota-T-β-MCA-FXR axis. *Pharmacology and Clinics of Chinese Materia Medica*. 1-8.

Yang, Y., Cao, S., Xu, W., Zang, C., Zhang, F., Xie, Y., Wu, C., (2022c). Dual modulation of gut bacteria and fungi manifests the gut-based anti-hyperlipidemic effect of *Coptidis Rhizoma*. *Biomed Pharmacother.* 153, 113542. doi: 10.1016/j.biopha.2022.113542

Yi, Z.Y., Chen, L., Wang, Y., He, D., Zhao, D., Zhang, S.H., Huang, J.H., (2022). The potential mechanism of Liu-Wei-Di-Huang Pills in treatment of type 2 diabetic mellitus: from gut microbiota to short-chain fatty acids metabolism. *Acta Diabetol.* 59 (10), 1295-1308. doi: 10.1007/s00592-022-01922-y

Yin, X., Peng, J., Zhao, L., Yu, Y., Zhang, X., Liu, P., Pang, X., (2013). Structural changes of gut microbiota in a rat non-alcoholic fatty liver disease model treated with a Chinese herbal formula. *Syst Appl Microbiol.* 36 (3), 188-196. doi: 10.1016/j.syapm.2012.12.009

Zhang, J.W., Chen Y.H., Luo S.F., Rao W.T., Liu, X., (2020). Effects of Congxinlunzhifang on blood lipid and intestinal flora in Apo^E-/-^ mice. *Traditional Chinese Drug Research and Clinical Pharmacology.* 31 (892-899). doi: doi:10.19378/j.issn.1003-9783.2020.08.003

Zhang, C., Liu, J., He, X., Sheng, Y., Yang, C., Li, H., Huang, K., (2019). *Caulis Spatholobi* ameliorates obesity through activating brown adipose tissue and modulating the composition of gut microbiota. *International Journal of Molecular Sciences.* 20 (20). doi: 10.3390/ijms20205150

Zhang, L., Wang, Y. J., Wu, F., Wang, X., Yi, F., Yuan, W., (2022a). MDG, an *Ophiopogon japonicus* polysaccharide, inhibits non-alcoholic fatty liver disease by regulating the abundance of *Akkermansia muciniphila*. *Int J Biol Macromol.* 196, 23-34. doi: 10.1016/j.ijbiomac.2021.12.036

Zhang, S., Wang, Y., Lu, F., Mohammed, S. A. D., Liu, H., Ding, S., Liu, S. M., (2022b). Mechanism of action of shenerjiangzhi formulation on hyperlipidemia induced by consumption of a high-fat diet in rats using network pharmacology and analyses of the gut microbiota. *Front Pharmacol.* 13, 745074. doi: 10.3389/fphar.2022.745074

Zhang, W., Cui, Y., Liu, Z., Wang, S., Yang, A., Li, X., Zhang, J., (2022c). *Astragalus membranaceus* ultrafine powder alleviates hyperuricemia by regulating the gut microbiome and reversing bile acid and adrenal hormone biosynthesis dysregulation. *Arabian Journal of Chemistry.* 15 (9). doi: 10.1016/j.arabjc.2022.103970

Zhang, X., Wang, H., Xie, C., Hu, Z., Zhang, Y., Peng, S., Fan, G., (2022d). Shenqi compound ameliorates type-2 diabetes mellitus by modulating the gut microbiota and metabolites. *J Chromatogr B Analyt Technol Biomed Life Sci.* 1194, 123189. doi: 10.1016/j.jchromb.2022.123189

Zhang, Y., Tang, K., Deng, Y., Chen, R., Liang, S., Xie, H., Yang, Q., (2018). Effects of shenling baizhu powder herbal formula on intestinal microbiota in high-fat diet-induced NAFLD rats. *Biomed Pharmacother*. 102, 1025-1036. doi: 10.1016/j.biopha.2018.03.158

Zhao, L., Ma, P., Peng, Y., Wang, M., Peng, C., Zhang, Y., Li, X., (2021a). Amelioration of hyperglycaemia and hyperlipidaemia by adjusting the interplay between gut microbiota and bile acid metabolism: *Radix Scutellariae* as a case. *Phytomedicine*. 83, 153477. doi: 10.1016/j.phymed.2021.153477

Zhao, T., Zhan, L., Zhou, W., Chen, W., Luo, J., Zhang, L., Liu, S., (2021b). The effects of Erchen decoction on gut microbiota and lipid metabolism disorders in Zucker diabetic fatty rats. *Front Pharmacol*. 12, 647529. doi: 10.3389/fphar.2021.647529

Zhao, X.Q., Guo, S., Lu, Y.Y., Hua, Y., Zhang, F., Yan, H., Duan, J. A., (2020). *Lycium barbarum* L. leaves ameliorate type 2 diabetes in rats by modulating metabolic profiles and gut microbiota composition. *Biomed Pharmacother*. 121, 109559. doi: 10.1016/j.biopha.2019.109559

Zhao, Z., Wang, J., Ren, W., Bian, Y., Wang, Y., Wang, L., Miao, J., (2022). Effect of Jiangan-Jiangzhi pill on gut microbiota and chronic inflammatory response in rats with non-alcoholic fatty liver. *Chem Biodivers*. 19 (5), e202100987. doi: 10.1002/cbdv.202100987

Zheng, J., Zhang, J., Guo, Y., Cui, H., Lin, A., Hu, B., Liu, H., (2020). Improvement on metabolic syndrome in high fat diet-induced obese mice through modulation of gut microbiota by sangguayin decoction. *Journal of Ethnopharmacology*. 246. doi: 10.1016/j.jep.2019.112225

Zhong, L.J., Xie, Z.S., Yang, H., Li, P., Xu, X.J., (2017). *Moutan Cortex* and *Paeoniae Radix* Rubra reverse high-fat-diet-induced metabolic disorder and restore gut microbiota homeostasis. *Chin J Nat Med*. 15 (3), 210-219. doi: 10.1016/S1875-5364(17)30037-7
